# Supplementary material for: Strategies for synergistic reduction of plastic leakage and greenhouse gas emissions in China
Source: Nat Commun. 2026 Feb 25;17:3178. doi: 10.1038/s41467-026-69893-0 (PMC13046867; doi:10.1038/s41467-026-69893-0)
Supplement: Supplementary file 1 — Supplementary Information [file 41467_2026_69893_MOESM1_ESM.pdf]

Supplementary information for

Strategies for synergistic reduction of plastic leakage and greenhouse gas emissions in China

Jingjing Bai[a], Zichun Huang[a], Xuewei Liu[b], Yuxin Liu[a], Lingyu Tai[a], Ziyang Lou[c], Johann Fellner[d], Wei Liu[e], Wenchao Ma[a, b, *]

[a] Key Laboratory of Agro-Forestry Environmental Processes and Ecological Regulation of Hainan Province, School of Environmental Science and Engineering, Hainan University, Haikou, 570228, China

[b] School of Environmental Science and Engineering, Tianjin University, Tianjin, 300072, China

[c] School of Environmental Science and Engineering, Shanghai Engineering Research Center of Solid Waste Treatment and Resource Recovery/ China Institute for Urban Governance, Shanghai Jiao Tong University, Shanghai, 200240, China

[d] Institute for Water Quality and Resource Management, TU Wien, Vienna, 1040, Austria

[e] Department of Environmental Science and Engineering, Nankai University Binhai College, Tianjin, 300072, China

*Corresponding author. Tel: 086 18602272358

E-mail address: mawc916@tju.edu.cn

Supplementary information:

Supplementary Notes: 1-2.

Supplementary Methods: 1-8.

Supplementary Tables: 1-45.

Supplementary Figures: 1-24.

Supplementary References.

Supplementary Note 1. System boundary

The systematic model developed in this work is centred on the Chinese plastics industry, subdivided into High Density Polyethylene (HDPE), Low Density Polyethylene (LDPE), Polypropylene (PP), Expanded Polystyrene (EPS), General Purpose Polystyrene (GPPS), Polyvinyl Chloride (PVC), Acrylonitrile Butadiene Styrene (ABS), Polyethylene Terephthalate (PET), Polyamide (PA), Polycarbonate (PC), Polyoxymethylene (POM), Polybutylene Terephthalate (PBT), Polyphenylene Sulfide (PPS), Others, a total of 14 categories. Substance metabolism, environmental leakage, and associated GHG emissions of plastics in China were also quantified for the period 1992-2021. We also projected the changes and synergies in consumption demand and environmental leakage of plastics and related GHG emissions under different measures for 2022-2060 based on a scenario analysis approach.

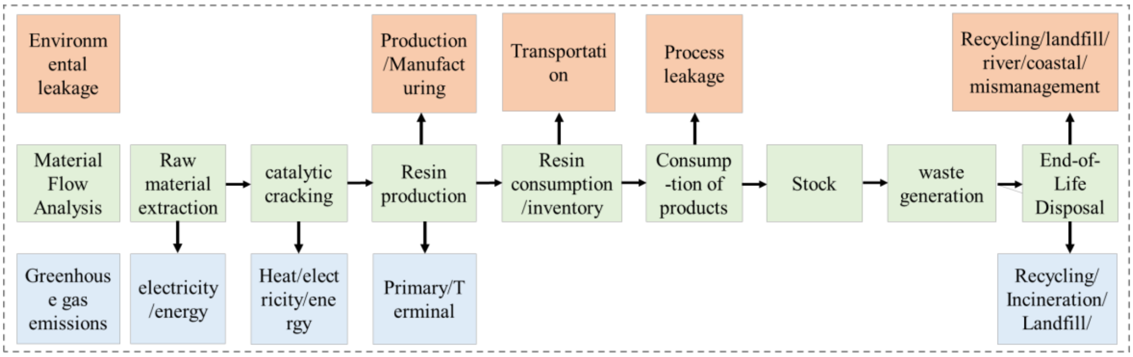

Supplementary Figure 1. System boundaries for research in the Chinese plastics industry.

Supplementary Note 2. Main data sources

China Plastics Industry Yearbook 2001-2021 details China's primary plastics production, import and export of HDPE, LDPE, PP, EPS, GPPS, PVC, ABS, PA, PC, POM, PBT, PPS, Others from 1992-2021. In addition, we supplemented and corroborated with data from other literature.

Factors such as inconsistent classification of plastic products and wastes, missing data, conflicting data and unclear data constitute major barriers to uncertainty and variability in the results of substance flow analyses and assessments. In this study, the data used were derived from official statistics (National Statistical Yearbook), the China Plastics Industry Processing Association (China Plastics Industry Yearbook) and peer-reviewed academic literature with sufficient resolution. The results of this study were obtained by integrating plastics-related data from all stages of the supply chain, covering different granularities and reporting frequencies. In addition, this study systematically explores for the first time the transformation pathways in the metabolism of plastic substances from fossil resources to chemical feedstocks to primary plastics (Supplementary Figure 2), taking into account the contents of primary plastics stocks, imported and exported finished plastics, and the consumption inputs and waste outputs of plastics types by industry, thus providing insights into complementing and refining the plastics substance metabolism mapping. Based on this, this study obtained more accurate data on the metabolism of plastic substances in China, which provides a solid data basis for the quantitative assessment of environmental leakage and GHG emissions.

Supplementary Table 1. Literature findings and characteristics of plastic environmental leakage and GHG emissions in global and Chinese contexts

Study area / Year of study	Value	Modeling & Methodology
Plastic leakage		
Plastic waste inputs from land into the ocean / 2010	9.6 / China: 2.425[1]	Global terrestrial plastic marine leakage quantified by integrating solid waste, population density, and economic status datasets.
River plastic emissions to the world’s oceans / 2015	1.78[2]	Developed a global riverine plastic emission model incorporating waste management, population density, and hydrology data.

Plastic waste leakage to the environment / 2016	30[3]	Assessed macroplastics from municipal sources and four terrestrial microplastic categories, focusing on environmental leakage pathways.
River plastic emissions to the world's oceans / 2017	0.4[4]	Constructed a global inventory of aquatic plastic debris across diverse river scales.
River plastic emissions to the world's oceans / 2018	0.13[5]	Developed a Human Development Index (HDI)-based predictive model for riverine plastic fluxes, calibrated and validated with field observations.
River plastic emissions to the world's oceans / 2020	1.0 / China: 0.071[6]	Integrated geospatial plastic waste, land use, wind, precipitation, and river data to quantify river-to-ocean transport probability.
Plastic waste leakage to the environment /2020	52.1/ China: 2.8[7]	Established a global macroplastic emissions inventory integrating emission mechanism modeling with empirical activity data.
Plastic waste inputs from land into the ocean / 2020	14.5[8]	Analysis of waste management data from 239 countries reveals significant infrastructure disparities between Global North/South, differentially impacting GHG emissions and marine plastic pollution.
Plastic waste leakage to the environment in China / 2020	China: 13.05[9]	Conducted dynamic MFA to quantify environmental plastic leakage in China.
River plastic emissions to the world's oceans / 2023	0.5[10]	Simulated riverine macro- and microplastic fluxes to coastal seas across >10,000 basins to identify dominant sources.
Plastic GHG emissions		
Global plastic GHG emissions /2015	1700[11]	Developed a dataset encompassing ten conventional and five bio-based plastics with lifecycle GHG emissions across multiple mitigation scenarios.
Global plastic GHG emissions / 2015	2000 / China: 820 (production perspective); 580 (consumption perspective)[12]	Applied enhanced MRIO analysis to: quantify the global plastics carbon footprint (1995–2030) and fossil resource footprint across production lifecycles; map interregional trade linkages; project emissions under the IEA 2°C/6°C

		scenario; evaluate PM health impacts, employment, and value-added socioeconomic dimensions.
Global plastic GHG emissions / 2020	2200[13]	Models the complete plastic lifecycle: upstream chemical production, polymer synthesis, product manufacturing, sectoral applications, end-of-life management.
China plastics GHG emissions / 2020	496.8[14]	Assessed material flows of China's primary synthetic resins via MFA across production, manufacturing, and end-of-life stages; quantified stage-specific GHG emissions using standardized accounting.
Global plastic GHG emissions / 2050 (project)	3350[15]	Developed a machine learning integrated model projecting global plastic production, use, and end-of-life trajectories to 2050.
synergistic reduction of plastic leakage and GHG emissions		
**This study** /2021	Leakage: 8.2 (Ter. 7.8, Aq. 0.4) / GHG emissions:679.1	Quantifies the synergy potential of plastics in reducing environmental leakage and GHG emissions. Developed a systematic assessment framework analyzing historical leakage/carbon emissions for 14 polymers across 10 Chinese sectors. Pioneered co-benefit potential and cost-effectiveness quantification under 14 scenarios (2021-2060).

*Environmental leakage in million metric tons per year; GHG emissions in million metric tons of CO_2-equivalent (Mt CO_2-eq).

Supplementary Table 2. China primary plastics production, import and export volume and consumption

Resin production, import and export volume and consumption	Data Literature Sources
HDPE	China Plastics Industry Yearbook[16]; Luan, X. et al. (2021)[17]; Chen, L. et al. (2023)[18]; An, J. et al. (2022)[19]; Chu, J. et al. (2023)[20]; Jiang, X. et al. (2020)[21]
LDPE	China Plastics Industry Yearbook[16]; Luan, X. et al. (2021)[17]; Chen, L. et al. (2023)[18]; Jiang, X. et al. (2020)[21]
PP	China Plastics Industry Yearbook[16]; Luan, X. et al. (2021)[17]; Chen, L. et al. (2023)[18]; Jiang, X. et al. (2020)[21]

EPS	China Plastics Industry Yearbook[16]; Luan, X. et al. (2021)[17]; Chen, L. et al. (2023)[18]; Chu, J. et al. (2022)[22]; Jiang, X. et al. (2020)[21]
GPPS	China Plastics Industry Yearbook[16]; Luan, X. et al. (2021)[17]; Chen, L. et al. (2023)[18]; Chu, J. et al. (2022)[22]; Jiang, X. et al. (2020)[21]
PVC	China Plastics Industry Yearbook[16]； Luan, X. et al. (2021)[17]; Chen, L. et al. (2023)[18]; Zhou, Y. et al. (2013)[23]; Liu, Y. et al. (2020)[24]; Chu, J. et al. (2022)[22]; Jiang, X. et al. (2020)[21]
ABS	China Plastics Industry Yearbook[16]; Luan, X. et al. (2021)[17]; Chen, L. et al. (2023)[18]; Chu, J. et al. (2022)[22]; Jiang, X. et al. (2020)[21]
PET	China Plastics Industry Yearbook[16]; Luan, X. et al. (2021)[17]; Chen, L. et al. (2023)[18]; Chu, J. et al. (2023) [25]
PA	China Plastics Industry Yearbook[16]; Luan, X. et al. (2021)[17]
PC	China Plastics Industry Yearbook[16]; Luan, X. et al. (2021)[17]
POM	China Plastics Industry Yearbook[16]; Luan, X. et al. (2021)[17]
PBT	China Plastics Industry Yearbook[16]; Luan, X. et al. (2021)[17]
PPS	China Plastics Industry Yearbook[16]; Luan, X. et al. (2021)[17]
Others	China Plastics Industry Yearbook[16]; Luan, X. et al. (2021)[17]

Supplementary Method 1. Material flow analyses

From chemical raw materials to primary plastics

Petrochemicals

The petrochemical industry chain is a chain that takes petroleum as the starting raw material, carries out cracking, generates basic chemical materials represented by ethylene, propylene, butadiene, benzene, toluene, xylene, and then produces a variety of organic chemical raw materials and synthetic materials from these basic chemical materials, and finally produces the end products.

Ethylene is a basic raw material for organic chemicals. In synthetic materials, it is used in large quantities for the production of polyethylene, vinyl chloride and polyvinyl chloride, ethylbenzene, styrene and polystyrene, and ethylene-propylene rubber.

Propylene can be used to produce a variety of important organic chemicals, synthetic resins, synthetic rubber and a variety of fine chemicals, etc., of which the largest amount is the production of polypropylene, in addition to acrylonitrile, isopropyl alcohol, phenol and acetone, butanol and octanol, acrylic acid and its lipids, as well as the production of propylene oxide and propylene glycol, epichlorohydrin, and synthetic glycerol and so on.

C4 fraction is mainly a mixture of various alkanes, olefins, diolefins and alkynes containing four carbon atoms.C4 fraction is a flammable gas, but is usually stored and transported in liquid form. It can be used as a fuel, or separated as a basic organic chemical raw material. The main components of C4 hydrocarbons of industrial significance are n-butane, isobutane, 1-butene, isobutene, 1,3-butadiene, C4 alkynes, etc., of which 1,3-butadiene is the most important.

About 10 per cent of benzene is used as a basic raw material for the manufacture of benzene intermediates. Benzene and ethylene produce ethylbenzene, which can be used to produce styrene for plastics. Benzene and propylene produce isopropylbenzene, which can be used in the isopropylbenzene process to produce acetone and phenol for resins and adhesives.

Natural gas chemicals

The natural gas chemical industry chain is an industry that produces chemical products from natural gas as a raw material, and is an integral part of the fuel chemical industry.

Coal chemicals

Coal chemical industry takes coal as raw material, through chemical processing to make coal into gas, liquid and solid products or semi-products, and then further processed into chemical and energy products industry. Coal chemical industry according to different process routes can be divided into coal coking (pyrolysis), coal calcium carbide, coal gasification and coal liquefaction. Coal to natural gas, coal to oil, coal to olefin and coal to alcohol and ether belong to the new type of modern coal chemical industry, which is currently a field of concern. China is generally a "coal-rich, oil-poor, gas-poor" country, and the structure of energy consumption is also obviously affected by it, and the development of coal to olefin is a better solution according to local conditions. In addition, the release of a number of coal chemical industry-related plans and programmes in 2017 also reflects China's determination to promote the development of the coal chemical industry. Supplementary Figure 2 shows the material flow from feedstock to primary plastics and primary plastics to end-of-pipe disposal.

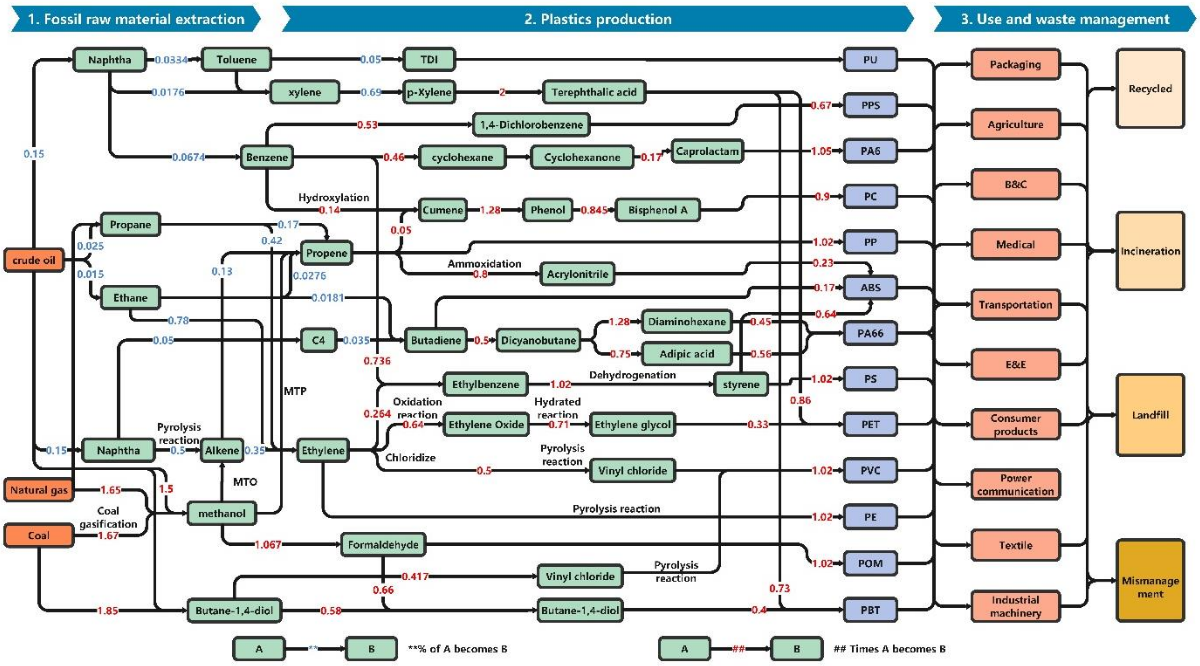

Supplementary Figure 2. Detailed flows of plastics from raw materials to primary plastics in China.

Primary plastics consumption

The consumption of primary plastics is modeled based on the mass balance of domestic production, international trade, and stock changes. This relationship is

expressed according to supplementary equation (1):

$$C_{k,t} = PR_{k,t} - S_{k,t} + I_{k,t} - E_{k,t} \quad (1)$$

$C_{k,t}$ refers to the consumption of primary plastics k in year t, $PR_{k,t}$、$I_{k,t}$ and $E_{k,t}$ are the domestic production, import and export of primary plastics k in year t, respectively, $S_{k,t}$ refers to the stock of primary plastics k in year t.

"Primary plastics inventory" refers to primary plastics raw materials or primary plastics products stored in warehouses. Inventory is an important concept that involves material management, cost control, production planning and other aspects of the enterprise. Proper inventory management can help enterprises to ensure the continuity of production and meet market demand in a timely manner, but also need to balance the cost of inventory and the risk of insufficient inventory.

Manufacture of plastic products

Each polymer flowing into different products can be calculated using a top-down approach. This calculation is performed according to supplementary equation (2):

$$C'_{k,p,t} = C_{k,t} * \alpha_{k,p,t} * (1 - \gamma) + I'_{k,p,t} - E'_{k,p,t} \quad (2)$$

$C'_{k,p,t}$ is the consumption of product p produced by primary plastic k in year t, $\alpha_{k,p,t}$ is the product diversion ratio, which indicates the proportion of primary plastic k flowing into product p in year t, and γ is the scrap rate in the manufacturing process, $I'_{k,p,t}$ and $E'_{k,p,t}$refer to the imports and exports of product p produced by primary plastic k in year t.

In addition to the import and export data on primary plastics mentioned, we also obtained import and export data on primary processed plastics and manufactured plastics for the period 1992-2021 from the online statistical data service platform of the General Administration of Customs of the People's Republic of China (customs.gov.cn) and the UN Comtrade database(UN Comtrade). These data will be based on the HS code (HS Code). These data are classified according to HS codes (i.e. customs codes) so that we can accurately analyse and understand the dynamics of our international trade in primary processed plastics and finished plastic products.

The consumption structure of plastics reflects the wide range of applications of various types of plastics in production and daily life. These plastics are moulded into a variety of forms and products through different processing methods to suit various needs. This diversified consumption structure not only meets market demands, but also promotes the continued development of the plastics industry. Supplementary Table 3 delineates allocation ratios from plastic polymer types to primary plastic products, this data is from China Plastics Industry Yearbook[16].

Supplementary Table 4 delineates allocation ratios from primary plastic products to industrial sectors[17,20–24,26–29].

Supplementary Table 3. Consumption structure by type of plastic.

HDPE								
	Films	Injection Moulding	Pipes	Drawing	Blow Moulding	Wire & Cable	Sheets	Others
2021	0.180	0.190	0.150	0.130	0.210	0.035	0.040	0.065
2020	0.180	0.190	0.150	0.130	0.210	0.035	0.040	0.065
2019	0.180	0.190	0.150	0.130	0.210	0.035	0.040	0.065
2018	0.180	0.190	0.150	0.130	0.210	0.035	0.040	0.065
2017	0.180	0.190	0.150	0.130	0.210	0.035	0.040	0.065
2016	0.180	0.190	0.150	0.130	0.210	0.035	0.040	0.065
2015	0.180	0.190	0.150	0.130	0.210	0.035	0.040	0.065
2014	0.177	0.190	0.146	0.133	0.210	0.035	0.043	0.066
2013	0.178	0.190	0.145	0.133	0.210	0.035	0.043	0.066
2012	0.178	0.190	0.145	0.133	0.210	0.035	0.043	0.066
2011	0.178	0.190	0.145	0.133	0.210	0.035	0.043	0.066
2010	0.178	0.190	0.145	0.133	0.210	0.035	0.043	0.066
2009	0.178	0.190	0.145	0.133	0.210	0.035	0.043	0.066
2008	0.178	0.190	0.145	0.133	0.210	0.035	0.043	0.066
2007	0.178	0.190	0.145	0.133	0.210	0.035	0.043	0.066
2006	0.178	0.190	0.145	0.133	0.210	0.035	0.043	0.066
2005	0.178	0.190	0.145	0.133	0.210	0.035	0.043	0.066
2004	0.178	0.190	0.145	0.133	0.210	0.035	0.043	0.066
2003	0.178	0.190	0.145	0.133	0.210	0.035	0.043	0.066

2002	0.178	0.190	0.145	0.133	0.210	0.035	0.043	0.066
2001	0.178	0.190	0.145	0.133	0.210	0.035	0.043	0.066
2000	0.178	0.190	0.145	0.133	0.210	0.035	0.043	0.066
1999	0.178	0.190	0.145	0.133	0.210	0.035	0.043	0.066
1998	0.178	0.190	0.145	0.133	0.210	0.035	0.043	0.066
1997	0.178	0.190	0.145	0.133	0.210	0.035	0.043	0.066
1996	0.178	0.190	0.145	0.133	0.210	0.035	0.043	0.066
1995	0.178	0.190	0.145	0.133	0.210	0.035	0.043	0.066
1994	0.178	0.190	0.145	0.133	0.210	0.035	0.043	0.066
1993	0.178	0.190	0.145	0.133	0.210	0.035	0.043	0.066
1992	0.178	0.190	0.145	0.133	0.210	0.035	0.043	0.066
LDPE								
	Packaging Films	Agricultural Films	Injection Moulding	Special Packaging Films	Coating	Wire & Cable	Tubing	Others
2021	0.515	0.195	0.090	0.080	0.040	0.030	0.030	0.020
2020	0.515	0.195	0.090	0.080	0.040	0.030	0.030	0.020
2019	0.515	0.195	0.090	0.080	0.040	0.030	0.030	0.020
2018	0.515	0.195	0.090	0.080	0.040	0.030	0.030	0.020
2017	0.515	0.195	0.090	0.080	0.040	0.030	0.030	0.020
2016	0.515	0.195	0.090	0.080	0.040	0.030	0.030	0.020
2015	0.515	0.195	0.090	0.080	0.040	0.030	0.030	0.020
2014	0.515	0.192	0.090	0.078	0.047	0.033	0.028	0.017
2013	0.512	0.195	0.092	0.076	0.046	0.033	0.028	0.016

2012	0.512	0.195	0.092	0.076	0.046	0.033	0.028	0.016
2011	0.512	0.195	0.092	0.076	0.046	0.033	0.028	0.016
2010	0.512	0.195	0.092	0.076	0.046	0.033	0.028	0.016
2009	0.512	0.195	0.092	0.076	0.046	0.033	0.028	0.016
2008	0.512	0.195	0.092	0.076	0.046	0.033	0.028	0.016
2007	0.512	0.195	0.092	0.076	0.046	0.033	0.028	0.016
2006	0.512	0.195	0.092	0.076	0.046	0.033	0.028	0.016
2005	0.512	0.195	0.092	0.076	0.046	0.033	0.028	0.016
2004	0.512	0.195	0.092	0.076	0.046	0.033	0.028	0.016
2003	0.512	0.195	0.092	0.076	0.046	0.033	0.028	0.016
2002	0.512	0.195	0.092	0.076	0.046	0.033	0.028	0.016
2001	0.512	0.195	0.092	0.076	0.046	0.033	0.028	0.016
2000	0.512	0.195	0.092	0.076	0.046	0.033	0.028	0.016
1999	0.512	0.195	0.092	0.076	0.046	0.033	0.028	0.016
1998	0.512	0.195	0.092	0.076	0.046	0.033	0.028	0.016
1997	0.512	0.195	0.092	0.076	0.046	0.033	0.028	0.016
1996	0.512	0.195	0.092	0.076	0.046	0.033	0.028	0.016
1995	0.512	0.195	0.092	0.076	0.046	0.033	0.028	0.016
1994	0.512	0.195	0.092	0.076	0.046	0.033	0.028	0.016
1993	0.512	0.195	0.092	0.076	0.046	0.033	0.028	0.016
1992	0.512	0.195	0.092	0.076	0.046	0.033	0.028	0.016
PP								
	Fabricated Products	Injection Moulding	BOPP	Fibre	Tube	CPP	Others	

2021	0.280	0.170	0.230	0.150	0.050	0.030	0.090	
2020	0.280	0.170	0.230	0.150	0.050	0.030	0.090	
2019	0.300	0.170	0.240	0.100	0.070	0.030	0.100	
2018	0.280	0.170	0.240	0.120	0.080	0.020	0.090	
2017	0.320	0.280	0.180	0.090	0.060	0.040	0.030	
2016	0.320	0.280	0.180	0.090	0.060	0.040	0.030	
2015	0.320	0.280	0.180	0.090	0.060	0.040	0.030	
2014	0.320	0.280	0.190	0.100	0.060	0.040	0.030	
2013	0.310	0.290	0.180	0.090	0.070	0.050	0.030	
2012	0.360	0.250	0.170	0.100	0.060	0.040	0.020	
2011	0.370	0.240	0.160	0.100	0.050	0.040	0.020	
2010	0.390	0.230	0.150	0.110	0.050	0.040	0.020	
2009	0.410	0.220	0.150	0.110	0.050	0.040	0.030	
2008	0.440	0.200	0.140	0.110	0.050	0.040	0.030	
2007	0.470	0.170	0.130	0.120	0.040	0.040	0.020	
2006	0.490	0.170	0.120	0.110	0.040	0.040	0.030	
2005	0.490	0.170	0.200	0.110	0.040	0.040	0.030	
2004	0.530	0.170	0.100	0.110	0.020	0.040	0.030	
2003	0.540	0.170	0.090	0.110	0.020	0.040	0.030	
2002	0.550	0.170	0.080	0.110	0.020	0.040	0.030	
2001	0.560	0.170	0.070	0.110	0.020	0.040	0.030	
2000	0.570	0.170	0.060	0.110	0.020	0.040	0.030	
1999	0.570	0.170	0.060	0.110	0.020	0.040	0.030	
1998	0.570	0.170	0.060	0.110	0.020	0.040	0.030	

1997	0.590	0.170	0.050	0.110	0.010	0.040	0.030	
1996	0.590	0.170	0.050	0.110	0.010	0.040	0.030	
1995	0.600	0.170	0.040	0.110	0.010	0.040	0.030	
1994	0.610	0.170	0.030	0.110	0.010	0.040	0.030	
1993	0.620	0.170	0.020	0.110	0.010	0.040	0.030	
1992	0.630	0.170	0.010	0.110	0.010	0.040	0.030	
EPS								
	Packaging Materials	Building insulation materials	Tableware	Others				
	0.450	0.480	0.010	0.060				
GPPS								
	Electronic appliances	Office Supplies	Daily necessities	Packaging Materials	Decoration Materials	Others		
	0.380	0.120	0.310	0.110	0.040	0.040		
PVC								
	Tubes	Profiles	Rigid Film, Sheet	Blow Moulding	Flexible Rods, Tubes			
2021	0.420	0.080	0.050	0.050	0.070			
2020	0.420	0.080	0.050	0.050	0.070			
2019	0.420	0.080	0.050	0.050	0.070			
2018	0.420	0.080	0.050	0.050	0.070			
2017	0.420	0.080	0.050	0.050	0.070			
2016	0.420	0.080	0.050	0.050	0.070			

2015	0.420	0.080	0.050	0.050	0.070			
2014	0.400	0.080	0.060	0.040	0.060			
2013	0.190	0.240	0.090	0.050	0.060			
2012	0.220	0.220	0.070	0.030	0.050			
2011	0.250	0.210	0.060	0.020	0.030			
2010	0.220	0.200	0.060	0.040	0.020			
2009	0.200	0.190	0.070	0.020	0.020			
2008	0.200	0.190	0.070	0.020	0.020			
2007	0.200	0.190	0.070	0.020	0.020			
2006	0.200	0.190	0.070	0.020	0.020			
2005	0.200	0.190	0.070	0.020	0.020			
2004	0.200	0.190	0.070	0.020	0.020			
2003	0.200	0.190	0.070	0.020	0.020			
2002	0.200	0.190	0.070	0.020	0.020			
2001	0.200	0.190	0.070	0.020	0.020			
2000	0.200	0.190	0.070	0.020	0.020			
1999	0.200	0.190	0.070	0.020	0.020			
1998	0.200	0.190	0.070	0.020	0.020			
1997	0.200	0.190	0.070	0.020	0.020			
1996	0.200	0.190	0.070	0.020	0.020			
1995	0.200	0.190	0.070	0.020	0.020			
1994	0.200	0.190	0.070	0.020	0.020			
1993	0.200	0.190	0.070	0.020	0.020			
1992	0.200	0.190	0.070	0.020	0.020			

PVC								
	Flexible Film, Sheet	Wire & Cable	Coating	Paving Materials	Other			
2021	0.160	0.060	0.030	0.030	0.050			
2020	0.160	0.060	0.030	0.030	0.050			
2019	0.160	0.060	0.030	0.030	0.050			
2018	0.160	0.060	0.030	0.030	0.050			
2017	0.160	0.060	0.030	0.030	0.050			
2016	0.160	0.060	0.030	0.030	0.050			
2015	0.160	0.060	0.030	0.030	0.050			
2014	0.150	0.060	0.040	0.030	0.090			
2013	0.130	0.070	0.040	0.030	0.120			
2012	0.140	0.100	0.040	0.030	0.110			
2011	0.150	0.120	0.040	0.030	0.100			
2010	0.150	0.110	0.020	0.020	0.160			
2009	0.150	0.110	0.030	0.030	0.180			
2008	0.150	0.110	0.030	0.030	0.180			
2007	0.150	0.110	0.030	0.030	0.180			
2006	0.150	0.110	0.030	0.030	0.180			
2005	0.150	0.110	0.030	0.030	0.180			
2004	0.150	0.110	0.030	0.030	0.180			
2003	0.150	0.110	0.030	0.030	0.180			
2002	0.150	0.110	0.030	0.030	0.180			
2001	0.150	0.110	0.030	0.030	0.180			

2000	0.150	0.110	0.030	0.030	0.180			
1999	0.150	0.110	0.030	0.030	0.180			
1998	0.150	0.110	0.030	0.030	0.180			
1997	0.150	0.110	0.030	0.030	0.180			
1996	0.150	0.110	0.030	0.030	0.180			
1995	0.150	0.110	0.030	0.030	0.180			
1994	0.150	0.110	0.030	0.030	0.180			
1993	0.150	0.110	0.030	0.030	0.180			
1992	0.150	0.110	0.030	0.030	0.180			
ABS								
	Household appliances	Office equipment	Transport	Light Industry	Building materials and others			
2021	0.550	0.190	0.150	0.090	0.020			
2020	0.550	0.190	0.150	0.090	0.020			
2019	0.550	0.190	0.150	0.090	0.020			
2018	0.550	0.190	0.150	0.090	0.020			
2017	0.550	0.190	0.150	0.090	0.020			
2016	0.550	0.190	0.150	0.090	0.020			
2015	0.550	0.190	0.150	0.090	0.020			
2014	0.550	0.195	0.150	0.085	0.020			
2013	0.530	0.215	0.150	0.085	0.020			
2012	0.530	0.215	0.150	0.085	0.020			
2011	0.530	0.215	0.150	0.085	0.020			

2010	0.530	0.215	0.150	0.085	0.020			
2009	0.530	0.215	0.150	0.085	0.020			
2008	0.530	0.215	0.150	0.085	0.020			
2007	0.530	0.215	0.150	0.085	0.020			
2006	0.530	0.215	0.150	0.085	0.020			
2005	0.530	0.215	0.150	0.085	0.020			
2004	0.530	0.215	0.150	0.085	0.020			
2003	0.530	0.215	0.150	0.085	0.020			
2002	0.530	0.215	0.150	0.085	0.020			
2001	0.530	0.215	0.150	0.085	0.020			
2000	0.530	0.215	0.150	0.085	0.020			
1999	0.530	0.215	0.150	0.085	0.020			
1998	0.530	0.215	0.150	0.085	0.020			
1997	0.530	0.215	0.150	0.085	0.020			
1996	0.530	0.215	0.150	0.085	0.020			
1995	0.530	0.215	0.150	0.085	0.020			
1994	0.530	0.215	0.150	0.085	0.020			
1993	0.530	0.215	0.150	0.085	0.020			
1992	0.530	0.215	0.150	0.085	0.020			
PET								
	PET fibers	PET bottles	PET film	PET engineering plastics				
2021	0.809	0.153	0.028	0.010				

2020	0.809	0.153	0.028	0.010				
2019	0.809	0.153	0.028	0.010				
2018	0.809	0.153	0.028	0.010				
2017	0.809	0.153	0.028	0.010				
2016	0.809	0.153	0.028	0.010				
2015	0.809	0.153	0.028	0.010				
2014	0.809	0.153	0.028	0.010				
2013	0.809	0.153	0.028	0.010				
2012	0.809	0.153	0.028	0.010				
2011	0.809	0.153	0.028	0.010				
2010	0.823	0.141	0.027	0.010				
2009	0.823	0.141	0.027	0.010				
2008	0.823	0.141	0.027	0.010				
2007	0.823	0.141	0.027	0.010				
2006	0.823	0.141	0.027	0.010				
2005	0.894	0.084	0.017	0.005				
2004	0.894	0.084	0.017	0.005				
2003	0.894	0.084	0.017	0.005				
2002	0.894	0.084	0.017	0.005				
2001	0.894	0.084	0.017	0.005				
2000	0.894	0.084	0.017	0.005				
1999	0.894	0.084	0.017	0.005				
1998	0.894	0.084	0.017	0.005				
1997	0.894	0.084	0.017	0.005				

1996	0.894	0.084	0.017	0.005				
1995	0.894	0.084	0.017	0.005				
1994	0.894	0.084	0.017	0.005				
1993	0.894	0.084	0.017	0.005				
1992	0.894	0.084	0.017	0.005				
PA								
	Electrical & Electronics	Automotive	Machinery Industry	Daily-use Hardware	Monofilament, brown silk	Nylon Powder	Film	Others
2021	0.380	0.250	0.130	0.070	0.040	0.030	0.040	0.070
2020	0.350	0.250	0.140	0.070	0.040	0.030	0.030	0.080
2019	0.350	0.250	0.140	0.070	0.040	0.030	0.030	0.080
2018	0.350	0.250	0.140	0.070	0.040	0.030	0.030	0.080
2017	0.320	0.260	0.150	0.080	0.040	0.030	0.030	0.100
2016	0.320	0.260	0.150	0.080	0.040	0.030	0.030	0.100
2015	0.320	0.260	0.150	0.080	0.040	0.030	0.030	0.100
2014	0.310	0.240	0.130	0.070	0.040	0.030	0.020	0.160
2013	0.310	0.240	0.130	0.070	0.040	0.030	0.020	0.160
2012	0.310	0.240	0.130	0.070	0.040	0.030	0.020	0.160
2011	0.300	0.250	0.130	0.070	0.030	0.030	0.040	0.150
2010	0.300	0.250	0.130	0.070	0.030	0.030	0.040	0.150
2009	0.300	0.250	0.130	0.070	0.030	0.030	0.040	0.150
2008	0.310	0.260	0.130	0.070	0.030	0.030	0.040	0.140
2007	0.320	0.270	0.130	0.070	0.030	0.030	0.050	0.120
2006	0.340	0.250	0.130	0.070	0.040	0.030	0.040	0.110

2005	0.340	0.250	0.130	0.070	0.040	0.030	0.040	0.110
2004	0.340	0.250	0.130	0.070	0.040	0.030	0.040	0.110
2003	0.330	0.240	0.130	0.070	0.040	0.030	0.060	0.110
2002	0.320	0.230	0.130	0.070	0.040	0.030	0.080	0.110
2001	0.310	0.210	0.130	0.070	0.040	0.030	0.100	0.110
2000	0.300	0.200	0.130	0.070	0.040	0.030	0.120	0.120
1999	0.300	0.200	0.130	0.070	0.040	0.030	0.120	0.120
1998	0.300	0.200	0.130	0.070	0.040	0.030	0.120	0.120
1997	0.300	0.200	0.130	0.070	0.040	0.030	0.120	0.120
1996	0.300	0.200	0.130	0.070	0.040	0.030	0.120	0.120
1995	0.300	0.200	0.130	0.070	0.040	0.030	0.120	0.120
1994	0.300	0.200	0.130	0.070	0.040	0.030	0.120	0.120
1993	0.300	0.200	0.130	0.070	0.040	0.030	0.120	0.120
1992	0.300	0.200	0.130	0.070	0.040	0.030	0.120	0.120
PC								
	Discs and lenses	Hollow sheets for sunlight panels, barriers, etc.	Packaging for drinking water barrels, bottles, etc.	Plates, tubes	Automotive			
2021	0.318	0.268	0.195	0.055	0.039			
2020	0.318	0.268	0.195	0.055	0.039			
2019	0.318	0.268	0.195	0.055	0.039			
2018	0.317	0.267	0.195	0.054	0.039			

2017	0.327	0.265	0.192	0.055	0.040			
2016	0.338	0.263	0.190	0.056	0.041			
2015	0.316	0.266	0.194	0.054	0.039			
2014	0.358	0.259	0.185	0.057	0.043			
2013	0.361	0.262	0.182	0.059	0.043			
2012	0.364	0.264	0.179	0.061	0.043			
2011	0.295	0.249	0.181	0.063	0.039			
2010	0.225	0.233	0.183	0.065	0.035			
2009	0.379	0.216	0.179	0.068	0.037			
2008	0.383	0.217	0.180	0.068	0.035			
2007	0.386	0.218	0.182	0.068	0.034			
2006	0.395	0.214	0.188	0.074	0.037			
2005	0.403	0.210	0.194	0.081	0.040			
2004	0.381	0.192	0.163	0.083	0.042			
2003	0.358	0.175	0.132	0.085	0.044			
2002	0.335	0.157	0.101	0.088	0.045			
2001	0.313	0.140	0.070	0.090	0.047			
2000	0.180	0.140	0.090	0.090	0.040			
1999	0.180	0.140	0.090	0.090	0.040			
1998	0.180	0.140	0.090	0.090	0.040			
1997	0.180	0.140	0.090	0.090	0.040			
1996	0.180	0.140	0.090	0.090	0.040			
1995	0.180	0.140	0.090	0.090	0.040			
1994	0.180	0.140	0.090	0.090	0.040			

1993	0.180	0.140	0.090	0.090	0.040			
1992	0.180	0.140	0.090	0.090	0.040			
PC								
	Safety glass	Instruments, electronic appliances	Industrial and mining accessories	Lighting Fixtures	Medical Instruments	Others		
2021	0.028	0.018	0.015	0.014	0.009	0.041		
2020	0.028	0.018	0.015	0.014	0.009	0.041		
2019	0.028	0.018	0.014	0.014	0.009	0.043		
2018	0.028	0.018	0.014	0.014	0.009	0.045		
2017	0.028	0.018	0.014	0.014	0.009	0.038		
2016	0.028	0.018	0.014	0.013	0.009	0.030		
2015	0.028	0.018	0.014	0.014	0.009	0.048		
2014	0.028	0.018	0.013	0.012	0.010	0.016		
2013	0.028	0.019	0.013	0.012	0.010	0.012		
2012	0.029	0.019	0.013	0.011	0.011	0.008		
2011	0.027	0.093	0.011	0.010	0.010	0.024		
2010	0.025	0.167	0.010	0.009	0.008	0.039		
2009	0.023	0.021	0.013	0.011	0.007	0.046		
2008	0.022	0.019	0.012	0.010	0.007	0.046		
2007	0.021	0.017	0.011	0.009	0.007	0.047		
2006	0.020	0.017	0.010	0.009	0.007	0.030		
2005	0.019	0.016	0.008	0.008	0.008	0.013		
2004	0.018	0.064	0.008	0.008	0.008	0.035		

2003	0.017	0.111	0.007	0.007	0.007	0.056		
2002	0.015	0.159	0.007	0.007	0.007	0.078		
2001	0.014	0.206	0.007	0.007	0.007	0.100		
2000	0.014	0.360	0.007	0.007	0.007	0.066		
1999	0.014	0.360	0.007	0.007	0.007	0.066		
1998	0.014	0.360	0.007	0.007	0.007	0.066		
1997	0.014	0.360	0.007	0.007	0.007	0.066		
1996	0.014	0.360	0.007	0.007	0.007	0.066		
1995	0.014	0.360	0.007	0.007	0.007	0.066		
1994	0.014	0.360	0.007	0.007	0.007	0.066		
1993	0.014	0.360	0.007	0.007	0.007	0.066		
1992	0.014	0.360	0.007	0.007	0.007	0.066		
POM								
	Automotive	Electrical & Electronics	Machinery	Consumer Goods	Others			
2021	0.220	0.270	0.190	0.200	0.120			
2020	0.211	0.271	0.183	0.206	0.128			
2019	0.203	0.273	0.176	0.213	0.135			
2018	0.194	0.274	0.169	0.219	0.143			
2017	0.186	0.275	0.163	0.225	0.150			
2016	0.181	0.276	0.159	0.228	0.154			
2015	0.177	0.276	0.156	0.231	0.158			
2014	0.173	0.277	0.152	0.234	0.161			
2013	0.169	0.278	0.149	0.238	0.165			

2012	0.164	0.278	0.145	0.241	0.169			
2011	0.160	0.279	0.142	0.244	0.173			
2010	0.156	0.279	0.138	0.247	0.176			
2009	0.152	0.280	0.135	0.250	0.180			
2008	0.143	0.281	0.128	0.256	0.188			
2007	0.134	0.283	0.121	0.263	0.195			
2006	0.126	0.284	0.114	0.269	0.203			
2005	0.117	0.285	0.108	0.275	0.210			
2004	0.109	0.286	0.101	0.281	0.218			
2003	0.100	0.288	0.094	0.288	0.225			
2002	0.092	0.289	0.087	0.294	0.233			
2001	0.083	0.290	0.080	0.300	0.240			
2000	0.096	0.320	0.155	0.295	0.144			
1999	0.096	0.320	0.155	0.295	0.144			
1998	0.096	0.320	0.155	0.295	0.144			
1997	0.096	0.320	0.155	0.295	0.144			
1996	0.096	0.320	0.155	0.295	0.144			
1995	0.096	0.320	0.155	0.295	0.144			
1994	0.096	0.320	0.155	0.295	0.144			
1993	0.096	0.320	0.155	0.295	0.144			
1992	0.096	0.320	0.155	0.295	0.144			
PBT								
	Automotive	Electrical & Electronics	Machinery	Consumer Goods	Others			

	0.410	0.260	0.160	0.080	0.090			
PPS								
	Electrical & Electronics	Automotive	Mechanical	Chemical	Others			
	0.720	0.110	0.080	0.030	0.060			
Others								
	Packaging	Agriculture	Construction	Medical	Transport			
	0.350	0.100	0.200	0.050	0.100			
Others								
	Electronic and electrical appliances	Daily necessities	Electricity Communication	Textile	Industrial Machinery			
	0.100	0.050	0.020	0.020	0.010			

Supplementary Table 4. Proportion of primary products of each plastic type entering each industry.

HDPE										
	Pack-age	Agricu-lture	B&C	Med-ical	Transpo rtation	E&E	Consu-mer	Electric power communi-cation	Textile	Industry Machinery
Films	0.5	0.3	0.1	0.1						
Injection Moulding	0.4		0.3	0.1	0.1	0.1				
Pipes			0.7				0.3			
Drawing	0.4		0.2				0.4			
Blow Moulding	0.4		0.15	0.1	0.1		0.25			
Wire & Cable								1		
Sheets			0.85				0.15			
Others	0.4		0.3	0.1	0.1		0.1			
LDPE										
	Pack-age	Agricu-lture	B&C	Med-ical	Transpo rtation	E&E	Consu-mer	Electric power communi-cation	Textile	Industry Machinery
Packaging Films	1									
Agricu-ltural Films		1								
Injection Moulding	0.5	0.2	0.2			0.1				
Special Packaging Films	1									

Coating	0.8		0.2							
Wire & Cable								1		
Tubing			0.4				0.6			
Others	0.25			0.1	0.1	0.1	0.25		0.2	
PP										
	Pack-age	Agricu-lture	B&C	Med-ical	Transportation	E&E	Consu-mer	Electric power communi-cation	Textile	Industry Machinery
Fabricated Products	0.6		0.4							
Injection Moulding	0.35		0.1		0.2	0.15	0.2			
BOPP	1									
Fibre			0.15		0.25		0.1		0.5	
Tube			0.8				0.2			
CPP	1									
Others	0.25		0.1		0.2	0.15	0.15		0.15	
EPS										
	Pack-age	Agricu-lture	B&C	Med-ical	Transportation	E&E	Consu-mer	Electric power communi-cation	Textile	Industry Machinery
Packaging Materials	1									

Building insulation materials			1							
Tableware	1									
Others	0.45				0.2	0.15	0.2			
GPPS										
Electronic appliances						1				
Office Supplies							1			
Daily necessities							1			
Packaging Materials	1									
Decoration Materials			1							
Others	0.1		0.15	0.1	0.1		0.55			
PVC										
	Pack-age	Agricu-lture	B&C	Med-ical	Transportation	E&E	Consu-mer	Electric power communi-cation	Textile	Industry Machinery
Tubes			0.65				0.2	0.15		
Profiles			0.6		0.15	0.1	0.15			
Rigid Film, Sheet	0.25		0.35		0.1		0.3			
Blow Moulding	0.35		0.15	0.1	0.15		0.25			
Flexible Rods, Tubes			0.45	0.1	0.1		0.35			
Flexible Film, Sheet	0.45		0.25		0.1	0.1	0.1			
Wire & Cable								1		

Coating			0.45		0.25	0.15	0.15			
Paving Materials			1							
Other				0.15			0.85			
ABS										
	Pack-age	Agricu-lture	B&C	Med-ical	Transpo rtation	E&E	Consu-mer	Electric power communi-cation	Textile	Industry Machinery
Household appliances						1				
Office equipment							1			
Transport					1					
Light Industry							1			
Building materials and others			1							
PET										
	Pack-age	Agricu-lture	B&C	Med-ical	Transpo rtation	E&E	Consu-mer	Electric power communi-cation	Textile	Industry Machinery
PET fibers	0.3		0.03		0.05		0.02		0.6	
PET bottles	1									
PET film	0.45				0.1	0.25	0.2			
PET engineering plastics	0.45		0.1		0.25	0.2				

PA										
	Pack-age	Agricu-lture	B&C	Med-ical	Transpo rtation	E&E	Consu-mer	Electric power communi-cation	Textile	Industry Machinery
Electrical & Electronics						1				
Automotive					1					
Machinery Industry										1
Daily-use Hardware										1
Monofi-lament, brown silk							0.3		0.45	0.25
PC										
	Pack-age	Agricu-lture	B&C	Med-ical	Transpo rtation	E&E	Consu-mer	Electric power communi-cation	Textile	Industry Machinery
Discs and lenses							1			
Hollow sheets for sunlight panels, barriers, etc.			1							
Packaging for drinking water barrels, bottles, etc.	1									
Plates, tubes			0.55		0.15	0.1	0.2			
Automotive					1					
Safety glass					1					

Instruments, electronic appliances						1				
Industrial and mining accessories										1
Lighting Fixtures						1				
Medical Instruments				1						
Others							1			
POM										
	Pack-age	Agricu-lture	B&C	Med-ical	Transpo rtation	E&E	Consu-mer	Electric power communi-cation	Textile	Industry Machinery
Automotive					1					
Electrical & Electronics						1				
Machinery										1
Consumer Goods							1			
Others							1			
PBT										
	Pack-age	Agricu-lture	B&C	Med-ical	Transpo rtation	E&E	Consu-mer	Electric power communi-cation	Textile	Industry Machinery
Automotive									1	
Electrical & Electronics					1					

Machinery						1				
Consumer Goods								1		
Others							1			
PPS										
	Pack-age	Agricu-lture	B&C	Med-ical	Transportation	E&E	Consu-mer	Electric power communi-cation	Textile	Industry Machinery
Electrical & Electronics						1				
Automotive					1					
Mechanical										1
Chemical										1
Others							1			
Others										
	Pack-age	Agricu-lture	B&C	Med-ical	Transportation	E&E	Consu-mer	Electric power communi-cation	Textile	Industry Machinery
Packaging	1									
Agriculture		1								
Construction			1							
Medical				1						
Transport					1					

Electronic and electrical appliances						1				
Daily necessities							1			
Electricity Communication								1		
Textile									1	
Industrial Machinery										1

Use of plastic products

During the use phase, plastics are distributed across various economic sectors in different functional forms. The consumption of plastics within a specific sector is determined by the allocation of products into that sector, adjusted for the international trade of plastic-containing goods. This sectoral consumption is calculated according to supplementary equation (3):

$$C''_{k,p,t} = \sum_{p=1}^{n}(C'_{k,p,t} * \beta_{k,p,q,t}) + I''_{k,p,t} - E''_{k,p,t} \quad (3)$$

$C''_{k,p,t}$ refers to the domestic consumption of plastic k in sector q in year t, $\beta_{k,p,q,t}$ is the sectoral occupancy rate, which represents the rate of plastic k flowing into the product p produced by sector q in year t, $I''_{k,p,t}$ and $E''_{k,p,t}$ are the imports and exports of plastic k into sector q in year t, where it is produced.

Supplementary Table 5. Plastics in electrical and electronic products.

Species	Range of application
ABS/HIPS	Refrigerator, Washing Machine, Air-conditioner, Television, Computer, Vacuum Cleaner, Electric Fan, Small Appliances
PP	Washing machines, hoovers, electric fans, small electrical appliances
Polyester	Electric motor
PTFE	Electric iron, electric heater
PU	Refrigerators, freezers, packaging materials
POM	Various plastic transmission parts

Supplementary Table 6. Percentage by weight of allied materials contained in major home appliances, electronic and electrical products.

Species	Televisions	Refrigerator	Air Conditioner
percentages /%	25	40	11
Species	Washing machine	Desktop computer	Vacuum Cleaner
percentages /%	36	22.9	60

Supplementary Table 7. Composition ratios by type of plastic in four types of appliances.

	Televisions	Refrigerator	Washing machine	Air Conditioner
PP	8.9	24.7	76.5	21.2
PVC	3.2	7.9	5.7	10.6
PS	84.5	26.3	6.2	31.9
AS	0	0	0	1.7
ABS	1.7	16.3	3.0	10.8
ASA	0	0	0	2.5

Polyester	0	0	2.0	3.7
Glassfiber reinforced plastics	0	0	0	8.4
PU	0	21.4	0	0
Others	1.7	3.4	6.6	9.2
Total	100%	100%	100%	100%

Supplementary Table 8. Proportion of various plastics used in the automotive industry.

Species	PP	PE	PU	PVC	ABS	PA	POM	FRP & Others
Passenger cars/%	27.6	10.5	14.5	13.2	13.2	3.9	1.3	15.8
Goods vehicles (including buses)/%	12.0	18.0	24.0	16.0	6.0	4.0	1.0	19.0

Supplementary Table 9. Analysis of Plastic Consumption in General Medium-sized Cars in China.

Species	PP	PE	EVA	PVC	ABS&alloy	PC
Medium-sized sedan average air consumption/(kg/vehicle)	63.0	8	8	6	15	2.5
Species	PBT	POM	PA	PU	Others	Total
Average empty consumption of medium-sized cars/(kg/vehicle)	8	3.5	3	7.5	10	141.5

Plastic products stock

Plastic products remain in use within society for varying durations, eventually accumulating in specific sectors as in-use inventory. The in-use stock represents the total mass of plastics currently serving their intended functions, and its dynamic change is a critical indicator for predicting future waste generation. The inventory level is determined by the balance between sectoral inflows and outflows according to supplementary equation (4):

$$S_{k,p,t} = \sum_{t_j}^{t} (C''_{k,p,t} - \sum_{t_j=1992}^{t} OF_{k,p,t}) \tag{4}$$

$S_{k,p,t}$ refers to the stock of plastic k in sector q in year t, $OF_{k,p,t}$ is the outflow of plastic k from sector q in year t. The outflow is the waste generated by the sector.

Plastic waste generation

We used data from Geyer, R. et al. (2017)[30] and Drewniok, M. et al. (2023)[31] estimating the mean μ and standard deviation σ of the lifetimes of various types of products. The evolution of plastic products in use is modeled using a log-normal distribution function, as specified in supplementary equation (5). The f(x,S,M) in the lognormal probability distribution function is a function of time (lifetime of the plastic product in use) and two parameters M and S. The f(x,S,M) in the lognormal probability distribution function is a function of time. are estimated from the mean value μ and standard deviation σ of the product lifetime according to supplementary equation (6) and (7).

The risk rate for a lognormal distribution can be interpreted as the instantaneous failure probability for age t. This risk function is then used in the inventory model to calculate the share of each category of plastic products of age t disposed of in any given year. where φ is the cumulative distribution function for the standard normal N (μ= 0, σ^2= 1). The mass of plastic waste discarded for each product category of age t is determined according to supplementary equation (8).

$$f(x,S,M) = \frac{1}{\sqrt{2\pi}Sx} e^{-\frac{(lnx-M)^2}{2S^2}} \tag{5}$$

$$\mu = e^{M+\frac{S^2}{2}} \tag{6}$$

$$\sigma = \sqrt{e^{S^2+2m}(e^{S^2}-1)} \tag{7}$$

$$OF_{k,p,t} = \sum_P \sum_{t=1992}^{\infty} S_{n-1,t} \frac{\frac{1}{\sqrt{2\pi}Sx} e^{-\frac{(lnx-M)^2}{2S^2}}}{1-\varphi(\frac{lnx-M}{S})} \tag{8}$$

Plastic waste disposal

The intertwined use of various plastic types in different industries results in the same type of plastic having different lifespans in different industries, resulting in different levels of plastic waste. This variation makes the management and recycling of plastic waste a complex challenge that requires waste treatment and resource recovery strategies tailored to the specific uses and waste stream characteristics of each industry.

The definition and technical characteristics of the "end-of-life" (EoL) path for plastic waste in China. Mechanical Recycling: Transforming single-type plastics (e.g., PET bottles, HDPE) back into recycled pellets or new products through physical processes like sorting, cleaning, shredding, and melt reprocessing. Relies on strict sorting and high cleanliness. Suitable for high-purity plastic waste. Incineration: Co-incineration of plastics with municipal solid waste to generate electricity or heat using thermal energy. Offers significant volume reduction. Landfilling: Co-disposal of plastics with other municipal solid waste in centralized sanitary landfills. Simple to operate but occupies land resources and offers no resource recovery. Mismanagement / Dumping: Plastic waste discarded irregularly into the natural environment (land, water bodies, or illegal dumpsites), not integrated into formal management systems.

Completely uncontrolled, directly causing ecological pollution. Chemical Recycling: Converts plastics into fuel oil, carbon black, or combustible syngas. Can handle mixed/low-value plastics but is not yet commercially scalable.

Recycle

Recovery rate

At present, the main domestic waste plastics from waste PET, waste packaging film and electrical and electronic products, etc., the data show that in 2021 China's waste PET recycling volume is the highest, amounting to 5.5 million tonnes, accounting for 28.9%, of which the waste PET bottles amounted to 4.0 million tonnes, as the main component, waste packaging film and and electrical and electronic products waste plastics were 3.6 million tonnes and 1.6 million tonnes, respectively, accounting for 18.9% and 8.4% respectively. In terms of the structure of waste plastic types, PET, PE and PP are still the main components, accounting for 31.9% (5.5 million tonnes), 20.6% (4 million tonnes) and 19.4% (3.8 million tonnes) of PET, PE and PP respectively in the first three years of 2021. Based on data from the National Bureau of Statistics and the Recycled Plastics Branch of the China Materials Recycling Association and related literature, we have refined the recycling rate of each type of plastic in different industries, taking 2021 as an example, as shown in the following chart.

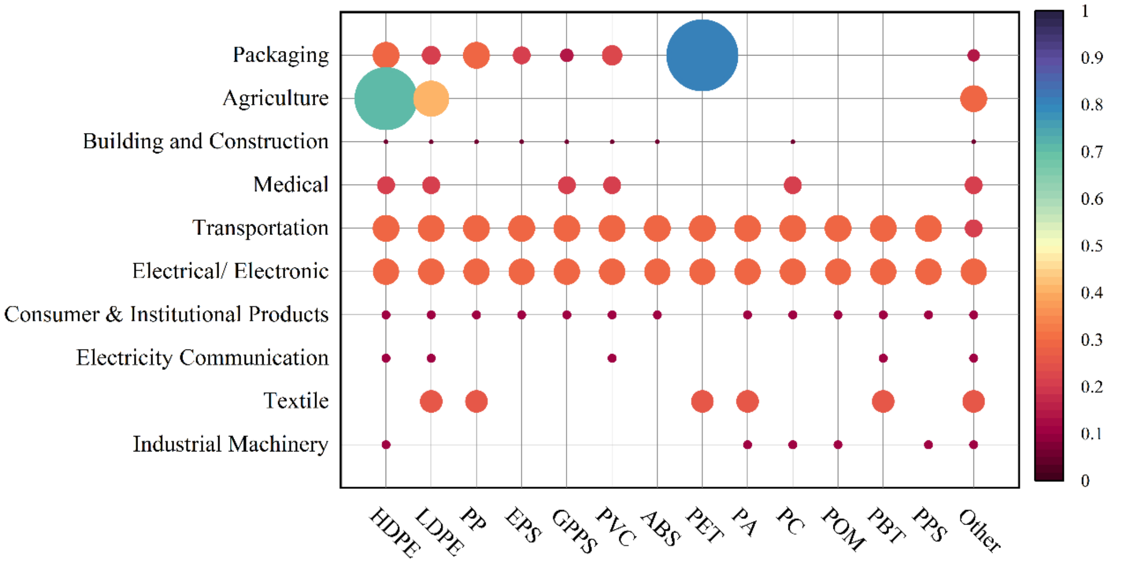

Supplementary Figure 3. Recycling Rates of Various Plastic Types by Industry in China, 2021

Recovery of lost production

In plastics recycling, there is a large loss of quality in the physical recycling of plastics due to the difficulty of separating mixed plastics, contamination and impurities, ageing and degradation of plastics, mechanical wear and tear during recycling and economic factors. According to the research (Haili Recycling Industrial Park, Jiaxing City, Zhejiang Province): in PET bottle recycling, excluding entrained

debris, removing packaging (1%), moisture (7%), caps (7-8%), labels (5-6%), crushed, rinsed, powdered, and defective flakes (3%), the rate of bottles gained is around 75%; the rate of valuable gained is at 95%; silt and sand, and entrained from other materials are also present. In addition, the quality losses of various types of plastics recycling given in the article by Drewniok, M. et al. (2023)[31] are supplementary table 10-11.

Supplementary Table 10. Loss of quality in plastics recycling.

Species	HDPE	LDPE	PP	PVC	Others
Recovery loss rate	34.6%	19.8%	12.9%	4%	50%

Incineration, landfill and mismanagement

Supplementary Table 11. End of life of plastic waste in China[17].

Years	Recycle	Incineration	Landfill	Mismanagement	Years	Recycle	Incineration	Landfill	Mismanagement
1992	0.04	0.02	0.26	0.68	2007	0.22	0.08	0.39	0.32
1993	0.03	0.02	0.37	0.58	2008	0.25	0.08	0.40	0.27
1994	0.04	0.03	0.39	0.54	2009	0.24	0.10	0.43	0.24
1995	0.05	0.03	0.45	0.47	2010	0.25	0.11	0.44	0.19
1996	0.06	0.03	0.41	0.50	2011	0.26	0.12	0.46	0.17
1997	0.07	0.03	0.45	0.45	2012	0.29	0.14	0.44	0.14
1998	0.08	0.03	0.47	0.43	2013	0.30	0.18	0.43	0.09
1999	0.09	0.03	0.49	0.39	2014	0.30	0.21	0.43	0.07
2000	0.10	0.03	0.47	0.40	2015	0.27	0.23	0.44	0.06
2001	0.12	0.03	0.44	0.42	2016	0.28	0.26	0.42	0.04
2002	0.14	0.02	0.40	0.44	2017	0.28	0.27	0.41	0.04
2003	0.17	0.02	0.36	0.45	2018	0.28	0.28	0.38	0.06
2004	0.20	0.03	0.36	0.42	2019	0.27	0.31	0.37	0.05
2005	0.23	0.04	0.34	0.40	2020	0.28	0.32	0.37	0.04
2006	0.21	0.06	0.34	0.39	2021	0.31	0.41	0.25	0.04

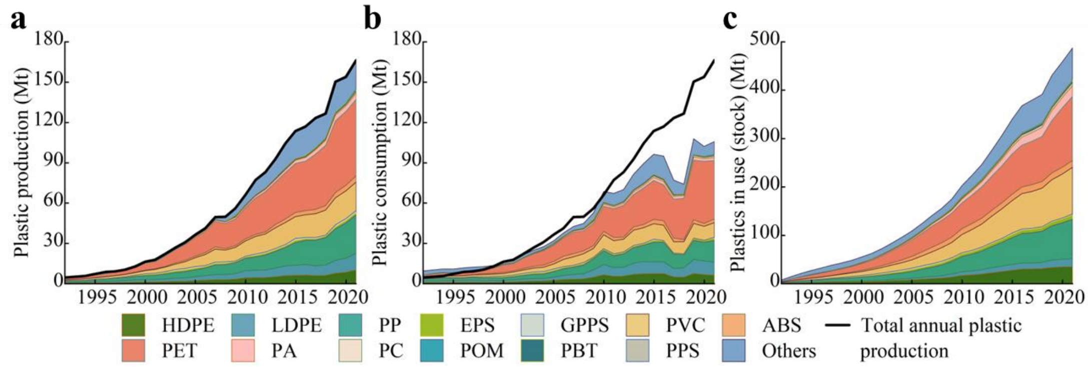

Supplementary Figure 4. Typological analysis of plastic production, consumption and in-use plastic stocks in China, 1992-2021. a, Annual production of plastics. b, Annual consumption of plastics. c, Annual amount of plastics in use.

Supplementary Method 2. Plastic environmental leakage

As an important basic material, plastics have been widely used in all aspects of the economy and society, bringing many conveniences to people's production and life, but also bringing great environmental challenges. However, plastic itself is not a pollutant, plastic waste leakage into the natural environment to form the plastic pollution. The essence of plastic pollution is that plastic waste leaks into the natural environment such as soil and water and is difficult to degrade, bringing visual pollution, soil damage, microplastics and other environmental hazards. Through literature review, this study determines the main pathways and stages of plastic environmental leakage.

Total amount of environmental leakage

The total amount of environmental leakage represents the cumulative mass of plastic polymers that are lost to natural ecosystems—including both terrestrial and aquatic environments—during the production, manufacturing, use, and disposal phases. This metric is a sum of the leakage categorized by the physical size of the plastic debris. The leakage is calculated according to supplementary equation (9):

$$Leakage_{total,k,t} = Leakage_{ma,k,t} + Leakage_{mi,k,t} \quad (9)$$

$Leakage_{total,k,t}$ refers to the total environmental leakage of type k plastics in year t, $Leakage_{ma,k,t}$ refers to the environmental leakage of macro-plastics of type k plastics in year t, $Leakage_{mj,k,t}$ refers to the environmental leakage of micro-plastics of type k plastics in year t.

Macroplastics environmental leakage

Macroplastic leakage refers to the mass of mismanaged plastic debris, typically with a diameter greater than 5 mm, that is lost to natural ecosystems through pathways such as open dumping or insufficient collection systems. This metric is categorized by the specific environmental compartment it enters, namely the terrestrial or aquatic environment. The leakage is calculated according to supplementary equation (10):

$$Leakage_{ma,k,t} = Terr_{ma,k,t} + Aqu_{ma,k,t} \quad (10)$$

$Terr_{ma,k,t}$ refers to the macroplastic leakage of type k plastics into the terrestrial environment in year t, $Aqu_{ma,k,t}$ refers to the macroplastic leakage of type k plastics into the aquatic environment in year t.

Microplastic environmental leakage

Microplastic environmental leakage represents the annual cumulative mass of polymers, generally defined as debris with a diameter smaller than 5 mm, that is released into natural systems. Within this study's framework, microplastics are categorized based on their entry into distinct environmental compartments, specifically land-based (terrestrial) and water-based (aquatic) systems. These emissions originate from diverse sources such as personal care products, textile laundering, and road dust. The total microplastic leakage is calculated according to

supplementary equation (11):

$$Leakage_{mi,k,t} = Terr_{mi,k,t} + Aqu_{mi,k,t} \tag{11}$$

$Terr_{mi,k,t}$ refers to the microplastic leakage of type k plastics to the terrestrial environment in year t, $Aqu_{mi,k,t}$ refers to the microplastic leakage of type k plastics to the aquatic environment in year t.

Macroplastic leakage to the terrestrial environment

Macroplastic leakage to the terrestrial environment refers to the total mass of mismanaged plastic debris entering land-based ecosystems due to technical losses or poor management across all life-cycle stages. This includes leakage occurring during production, manufacturing, transportation, and final disposal processes. The leakage is calculated according to supplementary equation (12):

$$Terr_{ma,k,t} = Prod_{k,t} \times Prod_{ma,\alpha} + Manuf_{k,t} \times Manuf_{ma,\alpha} + Transp_{k,t} \times Transp_{ma,\alpha} + Rec_{k,t} \times Rec_{ma,\alpha} + Landfill_{k,t} \times Landfill_{ma,\alpha} + Misman_{ma,k,t} \tag{12}$$

$Prod_{k,t}$, $Manuf_{k,t}$, $Transp_{k,t}$, $Rec_{k,t}$ and $Landfill_{k,t}$ refer to the amount of plastic of type k that was produced, manufactured, transported, recycled and landfilled in year t, $Misman_{ma,k,t}$ refers to the amount of plastic of type k that leaked into the land environment due to poor management of macroplastics in year t, $Prod_{ma,\alpha}$, $Manuf_{ma,\alpha}$, $Transp_{ma,\alpha}$, $Rec_{ma,\alpha}$ and $Landfill_{ma,\alpha}$ refers to the leakage rate of plastics to the land environment in the stages of production, manufacture, transportation, recycling, and landfill. Leakage rate of macroplastics to the landfill.

Macroplastic leakage to the aquatic environment

The aquatic leakage of macroplastics accounts for the mass of mismanaged plastic waste (MPW) that enters river systems, coastal regions, and oceanic environments. In this modeling framework, the total aquatic leakage is defined as the sum of contributions from four distinct pathways: aquaculture, fisheries, riverine transport, and coastal zones. This relationship is characterized by supplementary equation (13):

$$Aqu_{ma,k,t} = Aquac.ind_{k,t} + Fish.ind_{k,t} + River_{k,t} + Coastal_{k,t} \tag{13}$$

$Aquac.ind_{k,t}$, $Fish.ind_{k,t}$, $River_{k,t}$, $Rec_{k,t}$ and $Coastal_{k,t}$ refer to the amount of environmental leakage of type k plastics to the aquatic environment from aquaculture, fisheries, riverine areas, and coastal zones for the year t.

For riverine leakage, the accounting considers the proportion of the national territory covered by watersheds and a specific leakage rate for mismanaged plastics. The calculation is performed according to supplementary equation (14):

$$River_{k,t} = Misman_{k,t} \times \frac{Area_{land,river}}{Area_{land,total}} \times River_{ma,\alpha} \tag{14}$$

$Misman_{k,t}$ refers to the amount of mismanagement of type k plastics in year t, $Area_{land,river}$ refers to the total area covered by China's watersheds, $Area_{land,total}$

refers to the total area of China's national territory, $River_{ma,\alpha}$ refers to the rate of leakage of macroscopic plastics mismanagement from the river to the aquatic environment.

The aquatic leakage of macroplastics is modeled by considering the geographical distribution of mismanaged plastic waste (MPW) and the probability of its transport into water bodies. Coastal macroplastic leakage is estimated as a function of the total mismanaged plastic waste and the proportion of the population situated in coastal regions, which represents the risk of direct entry into marine systems. This pathway is calculated according to supplementary equation (15):

$$Coastal_{k,t} = \frac{Misman_{k,t}}{Pop_{land,total}} \times Area_{land,coastal} \times Coastal_{ma,\alpha} \quad (15)$$

$Pop_{land,total}$ refers to the total population of China in year t, $Area_{land,coastal}$ refers to the total coastal area of China, $Coastal_{ma,\alpha}$ refers to the leakage rate of poorly managed macroplastics from the coast to the aquatic environment.

The mass of mismanaged plastic waste on land is calculated according to supplementary equation (16):

$$Misman_{ma,k,t} = Misman_{k,t} - River_{k,t} - Coastal_{k,t} \quad (16)$$

Not all plastics that enter the environment enter the river system. mPW can be intercepted through clean-up efforts or can remain in the terrestrial environment through retention in the soil. In previous studies, Jambeck, J. R. et al[1] assumed that the leakage rate of plastics into marine systems is between 15-40%. However, Tramoy, R. et al[32], Mai, L. et al[5] argued that this leakage rate was taken to be too large and did not differentiate between riverine and coastal zone export. Based on field observations by Tramoy, R. et al[32] and van Emmerik, T. et al[33], Strokal, M. et al[10] used a leakage rate range of 1-5 per cent and only a small amount of MPW is exported from the river to coastal waters. Based on the Human Development Index (HDI) [5] of the watershed, the leakage rate for the watershed was set at 1-5%. The relevant correspondence is shown in Supplementary Table 12. Lau, W. W. Y. et al[3] state that 20% of poorly managed plastics enter water bodies at sites smaller than 1 km from the water body.

Supplementary Table 12. HDI corresponds to the relevant river leakage rate[32].

HDI range	HDl Classification	Leakage rate
0.000-0.499	Low	5%
0.500-0.799	Medium	4%
0.800-0.899	High	2%
0.900-1.000	Very High	1%

Leakage of microplastics to the terrestrial environment

The total annual mass of microplastics entering the terrestrial environment is quantified according to supplementary equation (17):

$$Terr_{mi,k,t} = Transp_{k,t} \times Transp_{mi,\alpha} + Indoor_{k,t} + Public_{k,t} + Landfill_{k,t} \times Landfill_{mi,\alpha} \quad (17)$$

$Indoor_{k,t}$ and $Public_{k,t}$ refer to the amount of residential indoor dust, public indoor dust, and mismanagement of type k plastics in year t, $Transp_{mi,\alpha}$ and $Landfill_{mi,\alpha}$ refer to the leakage rate of microplastics into the terrestrial environment during the transport phase and landfill phase.

Tyre microplastics are one of the main types of microplastics in the environment, mainly originating from rubber, which may be released into the environment either intentionally or unintentionally[34–36]. As this paper focuses on 14 types of plastic types and does not include rubber, the raw material for tyres, tyre microplastics are not considered in the accounting.

Microplastic leakage to the aquatic environment

The total mass of microplastics entering the aquatic environment is determined according to supplementary equation (18):

$$Aqu_{mi,k,t} = Prod_{k,t} \times Prod_{Mi,\alpha} + Manuf_{k,t} \times Manuf_{mi,\alpha} + PCP_{k,t} + laundry_{k,t} + Rec_{k,t} \times Rec_{mi,\alpha} \quad (18)$$

$PCP_{k,t}$ and $laundry_{k,t}$ refer to the microplastic generation of type k plastics from personal-care supply and laundry in year t, $Prod_{mi,\alpha}$, $Manuf_{mi,\alpha}$ and $Rec_{mi,\alpha}$ refer to the microplastic leakage rate to the aquatic environment from the production stage, the manufacturing stage and the recycling stage.

Supplementary Table 13. Sources of the data for model inputs.

<table>
<tr><th></th><th colspan="2">Plastic Life Stages</th><th>Leakage direction</th><th>Leakage Sources</th><th>Leakage Parameters</th><th>Sources</th></tr>
<tr><td rowspan="7">Macro-plastic</td><td colspan="2">$Prod_{Ma,\alpha}$</td><td>Terrestrial</td><td>Total</td><td>1.000%</td><td>Luan, X. et al[9]</td></tr>
<tr><td colspan="2">$Manuf_{Ma,\alpha}$</td><td>Terrestrial</td><td>Total</td><td>0.750%</td><td>Luan, X. et al[9]</td></tr>
<tr><td colspan="2">$Transp_{Ma,\alpha}$</td><td>Terrestrial</td><td>Total</td><td>0.750%</td><td>Luan, X. et al[9]</td></tr>
<tr><td rowspan="2">Use</td><td>$Aquac.ind_{k,t}$</td><td>Aquatic</td><td>-</td><td>-</td><td>Bai, M. et al[37], Luan, X. et al[9]</td></tr>
<tr><td>$Fish.ind_{k,t}$</td><td>Aquatic</td><td>-</td><td>-</td><td>Bai, M. et al[37], Luan, X. et al[9]</td></tr>
<tr><td colspan="2">$Rec_{Ma,\alpha}$</td><td>Terrestrial</td><td>Recycling</td><td>0.400%</td><td>Luan, X. et al[9]</td></tr>
<tr><td colspan="2">$Landfill_{Ma,\alpha}$</td><td>Terrestrial</td><td>Landfill</td><td>0.100%</td><td>Luan, X. et al[9]</td></tr>
</table>

	$Coastal_{ma,\alpha}$		Aquatic	Mismanagement	1-5%	Strokal, M. et al[10], Lau, W. W. Y. et al[3]
	$River_{ma,\alpha}$		Aquatic	Mismanagement	-	Strokal, M. et al[10]
	$Misman_{Ma,k,t}$		Terrestrial	-	-	-
Micro-plastic	$Prod_{Mi,\alpha}$		Aquatic	Total	0.050%	Luan, X. et al[9]
	$Manuf_{Mi,\alpha}$		Aquatic	Total	0.005%	Luan, X. et al[9]
	$Transp_{Mi,\alpha}$		Terrestrial	Total	0.002%	Luan, X. et al[9]
	Use	$PCP_{k,t}$	Aquatic	-	-	Strokal, M. et al[10], Lau, W. W. Y. et al[3]
		Laundry Products	Aquatic	Textiles consumption	0.120%	Strokal, M. et al[10], Lau, W. W. Y. et al[3]
		Indoor Dust	Terrestrial	-	0.080%	Strokal, M. et al[10], Luan, X. et al[9]
		Public Dust	Terrestrial	-	0.080%	Luan, X. et al[9]
	$Rec_{Mi,\alpha}$		Aquatic	Recycling	0.030%	Lau, W. W. Y. et al[3], Luan, X. et al[9]
	$Landfill_{Mi,\alpha}$		Terrestrial	Landfill	0.120%	Lau, W. W. Y. et al[3], Luan, X. et al[9]

Supplementary Method 3. Plastics-related GHG emissions

Total relevant GHG emissions

The GHG emission accounting framework for the entire life cycle of plastics is established by integrating ten distinct segments that span from upstream production to downstream disposal. This LCA approach quantifies the carbon footprint associated with fossil raw material extraction, monomer processing, polymerization, primary manufacturing, terminal manufacturing, recycling, incineration, and landfilling. Furthermore, the system incorporates carbon offsets derived from circular economy practices, specifically the displacement of virgin plastic production through material recycling and the substitution of fossil-based energy through incineration with energy recovery. The net total GHG emissions for each plastic type are calculated according to supplementary equation (19):

$$GHG_{total,g,k,t} = ME_{g,k,t} + MP_{g,k,t} + Poly_{g,k,t} + PM_{g,k,t} + TM_{g,k,t} + Rec_{g,k,t} + Inc_{g,k,t} + Landfill_{g,k,t} - RS_{g,k,t} - IS_{g,k,t} \quad (19)$$

$GHG_{total,g,k,t}$ is the total amount of GHG emissions associated with type k plastics in year t, $ME_{g,k,t}$ is the GHG emissions associated with the extraction of feedstock for type k plastics in year t, $MP_{g,k,t}$ is the GHG emissions associated with the processing of a single plastic of type k in year t, $Poly_{g,k,t}$ is the polymerisation-related GHG emissions of type k plastics in year t, $PM_{g,k,t}$ is the primary processing-related GHG emissions of type k plastics in year t, $TM_{g,k,t}$ is the end-process-related GHG emissions of type k plastics in year t, $Rec_{g,k,t}$ is the GHG emissions associated with the recycling of type k plastics in year t, $Inc_{g,k,t}$ is the GHG emissions associated with the incineration of type k plastics in year t, $Landfill_{g,k,t}$ is the landfill-related GHG emissions of type k plastics in year t, $RS_{g,k,t}$ is the GHG emissions reductions associated with the recycling substitution of type k plastics in year t, $IS_{g,k,t}$ is the GHG emissions reductions from incineration substitution of type k plastics in year t.

Raw material extraction

Oil, coal and natural gas are the main raw materials for the production of plastics. China is generally a ‘coal-rich, oil-poor, gas-poor’ country, a feature that significantly affects the structure of China's energy consumption. Therefore, we have determined the amount of raw material extraction and import demand related to the plastics industry based on the ratio of domestic raw material production to total consumption. The GHG emissions associated with fossil feedstock extraction for a specific polymer are calculated according to supplementary equation (20):

$$ME_{g,k,t} = Oil_{k,t} \times Oil_{p,t} \times Oil_{\beta,t} + Coal_{k,t} \times Coal_{p,t} \times Coal_{\beta,t} + NG_{k,t} \times NG_{p,t} \times NG_{\beta,t} \quad (20)$$

$Oil_{k,t}$, $Coal_{k,t}$, $NG_{k,t}$ refers to the amount of crude oil, coal and natural gas feedstock required for type k plastics in year t, $Oil_{p,t}$, $Coal_{p,t}$, $NG_{p,t}$ refers to the share of domestic production of crude oil, coal, and natural gas in total consumption

in year t (Supplementary Table 14), $Oil_{\beta,t}$, $Coal_{\beta,t}$, $NG_{\beta,t}$ Refers to GHG emissions per unit of product for crude oil, coal, and natural gas in year t (Supplementary Table 15).

Supplementary Table 14. Domestic self-production rate of three major fossil raw materials(%)[38].

Year	Oil	Coal	Natural Gas
1992	0.606	0.972	1.000
1993	0.606	0.972	1.000
1994	0.606	0.972	1.000
1995	0.606	0.972	1.000
1996	0.606	0.972	1.000
1997	0.606	0.972	1.000
1998	0.606	0.972	1.000
1999	0.606	0.972	1.000
2000	0.606	0.972	1.000
2001	0.606	0.972	1.000
2002	0.606	0.972	1.000
2003	0.606	0.972	1.000
2004	0.606	0.972	1.000
2005	0.603	0.972	1.000
2006	0.573	0.950	1.000
2007	0.547	0.950	0.982
2008	0.536	0.966	0.988
2009	0.497	0.959	0.953
2010	0.474	0.982	0.887
2011	0.461	0.968	0.785
2012	0.444	0.958	0.739
2013	0.431	0.936	0.709
2014	0.410	0.937	0.696
2015	0.392	0.937	0.697
2016	0.350	0.877	0.659
2017	0.322	0.900	0.618
2018	0.300	0.930	0.569
2019	0.284	0.957	0.576
2020	0.280	0.964	0.597
2021	0.275	0.960	0.571

Supplementary Table 15. Domestic self-production rate of three major fossil raw materials.

Fossil material	GHG emissions per unit of product
Oil	0.44[39]
Coal	2.29[39]
Natural Gas	17.4[39]

Chemical monomer processing

The total emissions for this segment are calculated as the sum of the emissions from each individual monomer utilized in the production of a given plastic type, according to supplementary equation (21):

$$MP_{g,k,t} = \sum MPF_{k,i,t} \times MPF_{\beta,i,t} \tag{21}$$

$MPF_{k,i,t}$ refers to the consumption of monomer I in year t for type k plastics, $MPF_{\beta,i,t}$ refers to the GHG emission parameter of monomer I in year t.

Supplementary Table 16. China's power system energy consumption ration (%)[38].

	Hydropower	Coal Power	Nuclear Power	Wind Power	Photovoltaic
1980	0.19	0.81	0	0	0
1981	0.19	0.81	0	0	0
1982	0.19	0.81	0	0	0
1983	0.19	0.81	0	0	0
1984	0.19	0.81	0	0	0
1985	0.22	0.78	0	0	0
1986	0.22	0.78	0	0	0
1987	0.22	0.78	0	0	0
1988	0.22	0.78	0	0	0
1989	0.22	0.78	0	0	0
1990	0.2	0.8	0	0	0
1991	0.2	0.8	0	0	0
1992	0.2	0.8	0	0	0
1993	0.2	0.8	0	0	0
1994	0.2	0.8	0	0	0
1995	0.19	0.8	0.01	0	0
1996	0.19	0.8	0.01	0	0
1997	0.19	0.8	0.01	0	0
1998	0.19	0.8	0.01	0	0
1999	0.19	0.8	0.01	0	0
2000	0.18	0.81	0.01	0	0
2001	0.18	0.81	0.01	0	0

2002	0.17	0.82	0.01	0	0
2003	0.15	0.83	0.02	0	0
2004	0.15	0.83	0.02	0	0
2005	0.16	0.82	0.02	0	0
2006	0.15	0.83	0.02	0	0
2007	0.14	0.83	0.03	0	0
2008	0.16	0.81	0.03	0	0
2009	0.16	0.82	0.01	0.01	0
2010	0.16	0.81	0.02	0.01	0
2011	0.14	0.82	0.02	0.02	0
2012	0.17	0.79	0.02	0.02	0
2013	0.17	0.79	0.01	0.03	0
2014	0.19	0.76	0.02	0.03	0
2015	0.19	0.74	0.03	0.03	0.01
2016	0.2	0.72	0.03	0.04	0.01
2017	0.19	0.71	0.03	0.05	0.02
2018	0.18	0.7	0.04	0.05	0.03
2019	0.18	0.69	0.04	0.06	0.03
2020	0.18	0.68	0.05	0.06	0.03
2021	0.18	0.68	0.05	0.06	0.03

Supplementary Table 17. Energy consumption ration for industrial steam production in China (%)[38].

	Coal	Natural gas	Solid waste
1980	0.9	0.1	0
1981	0.9	0.1	0
1982	0.9	0.1	0
1983	0.9	0.1	0
1984	0.9	0.1	0
1985	0.9	0.1	0
1986	0.9	0.1	0
1987	0.9	0.1	0
1988	0.9	0.1	0
1989	0.9	0.1	0
1990	0.9	0.1	0
1991	0.9	0.1	0
1992	0.9	0.1	0
1993	0.9	0.1	0
1994	0.9	0.1	0
1995	0.9	0.1	0

1996	0.9	0.1	0
1997	0.9	0.1	0
1998	0.9	0.1	0
1999	0.9	0.1	0
2000	0.9	0.1	0
2001	0.9	0.1	0
2002	0.9	0.1	0
2003	0.9	0.1	0
2004	0.9	0.1	0
2005	0.9	0.1	0
2006	0.9	0.1	0
2007	0.9	0.1	0
2008	0.9	0.1	0
2009	0.9	0.1	0
2010	0.9	0.1	0
2011	0.9	0.1	0
2012	0.9	0.1	0
2013	0.9	0.1	0
2014	0.9	0.1	0
2015	0.9	0.1	0
2016	0.9	0.1	0
2017	0.7	0.3	0
2018	0.7	0.3	0
2019	0.7	0.3	0
2020	0.7	0.3	0
2021	0.7	0.3	0

Supplementary Table 18. Fuel combustion energy consumption ratios in China (%)[38].

	Coal	Natural gas	Solid waste	Primary power and others
1992	0.757	0.175	0.019	0.049
1993	0.747	0.182	0.019	0.052
1994	0.75	0.174	0.019	0.057
1995	0.746	0.175	0.018	0.061
1996	0.735	0.187	0.018	0.06
1997	0.714	0.204	0.018	0.064
1998	0.709	0.208	0.018	0.065
1999	0.706	0.215	0.02	0.059
2000	0.685	0.22	0.022	0.073
2001	0.68	0.212	0.024	0.084

2002	0.685	0.21	0.023	0.082
2003	0.702	0.201	0.023	0.074
2004	0.702	0.199	0.023	0.076
2005	0.724	0.178	0.024	0.074
2006	0.724	0.175	0.027	0.074
2007	0.725	0.17	0.03	0.075
2008	0.715	0.167	0.034	0.084
2009	0.716	0.164	0.035	0.085
2010	0.692	0.174	0.04	0.094
2011	0.702	0.168	0.046	0.084
2012	0.685	0.17	0.048	0.097
2013	0.674	0.171	0.053	0.102
2014	0.658	0.173	0.056	0.113
2015	0.658	0.164	0.058	0.12
2016	0.622	0.187	0.061	0.13
2017	0.606	0.189	0.069	0.136
2018	0.59	0.189	0.076	0.145
2019	0.577	0.19	0.08	0.153
2020	0.569	0.188	0.084	0.159
2021	0.56	0.19	0.09	0.17

Supplementary Table 19. GHG emission parameters per unit of monomer production in China (t CO_2 -eq per t).

Type of monomer	Electricity consumption	Heat consumption	Fuel combustion	Waste disposal
Ethylene[11,13,14,40]	0.109	0.073	0.646	0.015
Propylene[11,13,14,40]	0.109	0.073	0.646	0.015
Styrene[11,13,14,40]	0.208	0.818	1.166	0.202
Vinyl chloride[11,13,14,40]	0.116	0.629	0.617	0.036
Butadiene[11,13,14,40]	0.267	0.923	0.659	0.013
Acrylonitrile[11,13,14,40]	0.516	0.214	0.692	0.147
Terephthalic Acid[11,13,14,40]	0.709	1.116	1.183	0.000
Ethylene Glycol[11,13,14,40]	0.237	0.782	0.630	0.000
Caprolactam[41]	1.699	4.075	5.989	0.502
Hexanediamine[42]	0.257	0.596	0.899	0.073
Adipic acid[42]	0.257	0.596	0.899	0.073
Liquid Chlorine[43]	0.203	0.000	0.123	0.009
Bisphenol A[43]	1.721	0.000	1.041	0.078
Sodium hydroxide[43]	0.000	0.000	0.354	0.027

Dimethyl carbonate[43]	0.164	0.000	0.735	0.020
Phenol[43]	0.060	0.000	0.024	0.002
Formaldehyde[11]	0.147	0.352	0.517	0.043
Butylene Glycol[11]	0.257	0.596	0.899	0.009
Dichlorobenzene[11]	0.257	0.596	0.899	0.009
Sodium Sulphide[11]	0.257	0.596	0.899	0.009

Monomer polymerization

The emissions from monomer polymerization are calculated according to supplementary equation (22):

$$Poly_{g,k,t} = \sum PR_{k,t} \times Poly_{\beta,k,t} \quad (22)$$

$Poly_{\beta,k,t}$ refers to the GHG emission parameter of 52olymerization production of type k plastic in year t.

Supplementary Table 20. GHG emission parameters for plastics polymerization in China (t CO_2 -eq per t)[14,40–43].

PE	PP	PS	PVC	ABS	PET	PA	PC	POM	PBT	PPS	Others
0.655	0.647	0.420	0.652	0.749	1.117	0.705	1.370	0.555	0.705	0.705	0.705

Primary production and processing

Primary production and processing represent the transformative stage where polymers are converted into intermediate plastic materials or semi-finished products. This phase involves various energy-intensive industrial processes, such as extrusion or pelletization, which contribute to the overall carbon footprint of the plastic life cycle. The GHG emissions associated with this stage are determined by the domestic production volume and the specific processing emission intensity for each polymer type. These emissions are calculated according to supplementary equation (23):

$$PM_{g,k,t} = C_{k,t} \times PM_{\beta,k,t} \quad (23)$$

$PM_{\beta,k,t}$refers to the GHG emission parameter for the primary processing of type k plastics in year t.

Supplementary Table 21. GHG emission parameters for primary processing of plastics in China (t CO_2 -eq per t)[14].

Film	Pipe	IMP	BMP	Fiber	Cable	Foam	Leather	Others
0.47	0.34	1.28	0.54	0.91	0.44	0.10	0.91	0.91

Terminal production and processing

The total emissions for this stage are calculated by multiplying the mass of each plastic type flowing into the manufacturing of specific products by their corresponding terminal processing emission intensities. These emissions are

determined according to supplementary equation (24):

$$TM_{g,k,t} = C'_{k,p,t} \times TM_{\beta,k,t} \tag{24}$$

$TM_{\beta,k,t}$ Refers to the GHG emission parameters of type k plastics for end-use manufacturing in year t.

Supplementary Table 22. Parameters of GHG emissions from Terminal production and processing of plastics in China (t CO_2 -eq per t)[14].

Package	Construction	Transportation	Electronics	Agriculture	Textiles	Others
0.04	0.33	0.43	0.15	0.25	0.17	0.23

Recycling

The recycling process involves the collection, sorting, and mechanical or chemical reprocessing of plastic waste into secondary materials. This stage incurs GHG emissions primarily through the energy consumed during transport and reprocessing operations. To quantify these impacts, the total emissions are calculated based on the mass of each plastic type successfully diverted to recycling streams and their corresponding emission intensities. The emissions associated with the recycling stage are determined according to supplementary equation (25):

$$Rec_{g,k,t} = Rec_{k,t} \times Rec_{\beta,k,t} \tag{25}$$

$Rec_{\beta,k,t}$ refers to the GHG emissions parameter of type k plastic at the recycling stage in year t.

Supplementary Table 23. Parameters for GHG emissions from plastic waste recycling (t CO_2 -eq per t)[11,29].

disposal technology	Recycling
GHG emissions	0.906

Incineration

GHG emissions from the incineration stage originate from the thermal combustion of the carbon content within plastic polymers. The total emissions for this disposal pathway are determined by the mass of plastic waste diverted to incineration and its corresponding emission intensity, as expressed in supplementary equation (26):

$$Inc_{g,k,t} = Inc_{k,t} \times Inc_{\beta,k,t} \tag{26}$$

$Inc_{\beta,k,t}$ refers to the GHG emission parameters of type k plastics at the incineration stage in year t.

Supplementary Table 24. Parameters for GHG emissions from plastic waste incineration (t CO_2 -eq per t)[11,29].

disposal technology	Incineration
GHG emissions	2.351

Landfill

GHG emissions from the landfill stage are associated with the long-term sequestration of carbon within plastic polymers and the potential fugitive emissions occurring during waste handling and containment. The total emissions for this disposal pathway are calculated based on the mass of plastic waste directed to sanitary landfills and its corresponding emission intensity, as expressed in supplementary equation (27):

$$Landfill_{g,k,t} = Landfill_{k,t} \times Landfill_{\beta,k,t} \quad (27)$$

$Landfill_{\beta,k,t}$ refers to the GHG emission parameters of type k plastics at the landfill stage in year t.

Supplementary Table 25. Parameters for GHG emissions from plastic waste Landfill (t CO_2 -eq per t)[11,29].

disposal technology	Landfill
GHG emissions	0.089

Supplementary Method 4. Synergistic analysis

Supplementary Table 26. Environmental leakage and GHG emissions at various stages of plastics.

	Environmental leakage	GHG
Production	Production	Raw material extraction
		Monomer Production
		Polymer production
Manufacturing and transport	Manufacturing	Primary Processing
	Transport	Terminal Manufacturing
Use	Personal care products	-
	Textiles	
	Indoor dust	
	Public dust	
	Aquaculture	
	Fisheries	
Disposal	Recycling	Recycling
	Landfill	Incineration
	Coastal areas	Landfill
	Riverine areas	Recycling Alternatives
	Mismanagement	Incineration Alternative

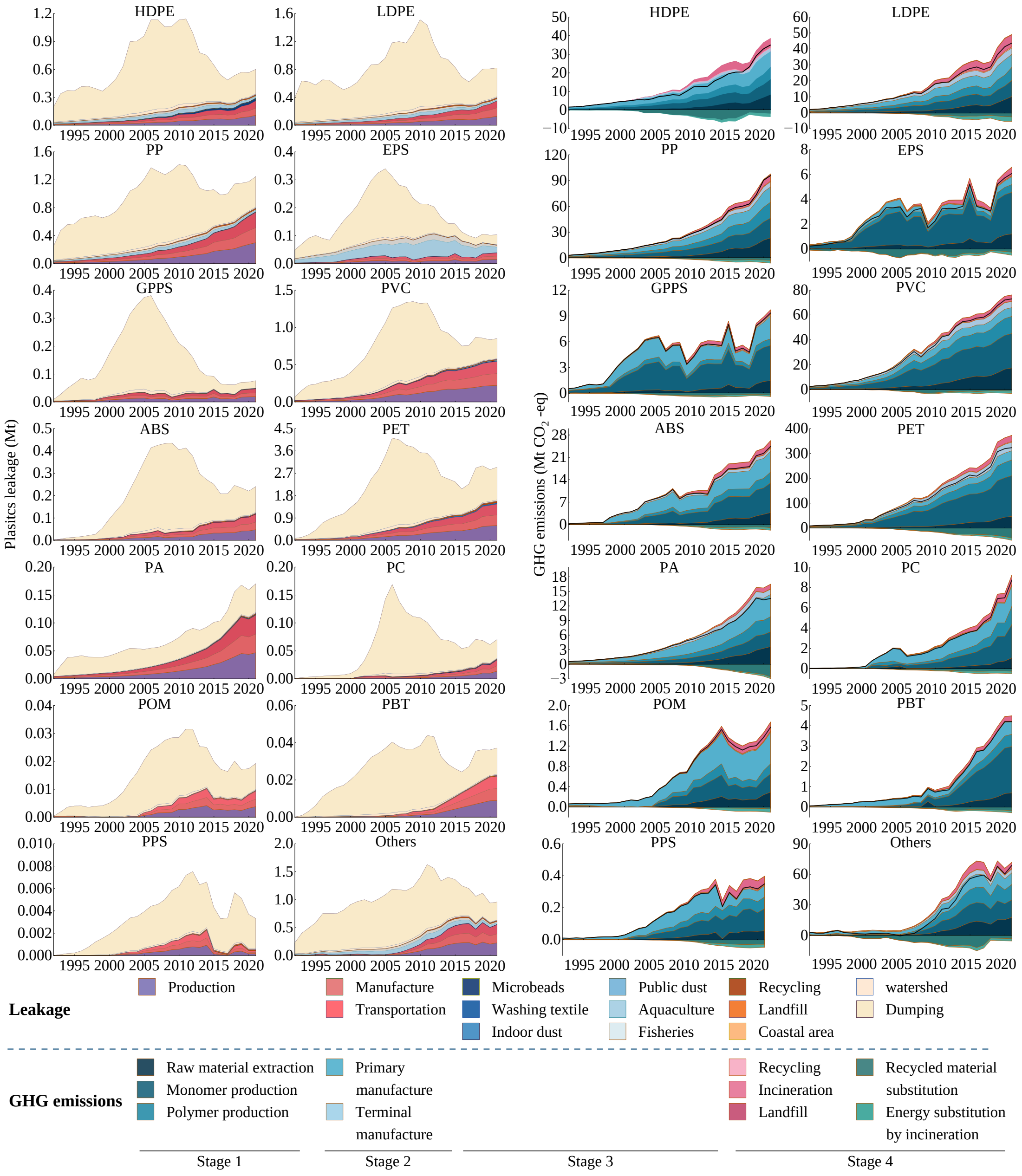

Supplementary Figure 5. Environmental leakage and associated GHG emissions from 14 plastics in China from 1992 to 2021. The four groups of colors represent the four stages, each group representing a different process within that stage. The black line indicates net GHG emissions.

a

			HDPE	LDPE	PP	EPS	GPPS	PVC	ABS	PET	PA	PC	POM	PBT	PPS	Others
Environmental leakage	Stage 1	Production	0.11	0.13	0.30	0.02	0.02	0.22	0.05	0.60	0.05	0.01	0.00	0.01	0.00	0.23
	Stage 2	Manufacture	0.08	0.12	0.21	0.01	0.01	0.16	0.03	0.43	0.03	0.01	0.00	0.01	0.00	0.16
		Transportation	0.08	0.10	0.22	0.01	0.01	0.16	0.03	0.43	0.03	0.01	0.00	0.01	0.00	0.16
	Stage 3	Personal-care supply	0.03	0.00	0.00	0.00	0.00	0.00	0.00	0.00	0.00	0.00	0.00	0.00	0.00	0.00
		Textile washing	0.00	0.00	0.00	0.00	0.00	0.00	0.00	0.04	0.00	0.00	0.00	0.00	0.00	0.00
		Indoor dust	0.00	0.00	0.01	0.00	0.00	0.01	0.00	0.00	0.00	0.00	0.00	0.00	0.00	0.00
		Public dust	0.00	0.00	0.01	0.00	0.00	0.01	0.00	0.00	0.00	0.00	0.00	0.00	0.00	0.00
		Aquaculture	0.02	0.02	0.02	0.02	0.00	0.00	0.00	0.00	0.00	0.00	0.00	0.00	0.00	0.03
		Fisheries	0.01	0.01	0.01	0.01	0.00	0.00	0.00	0.00	0.00	0.00	0.00	0.00	0.00	0.02
	Stage 4	Recovery	0.01	0.01	0.01	0.00	0.00	0.01	0.00	0.06	0.00	0.00	0.00	0.00	0.00	0.01
		Landfill	0.00	0.01	0.01	0.00	0.00	0.01	0.00	0.02	0.00	0.00	0.00	0.00	0.00	0.01
		Coastal	0.00	0.00	0.00	0.00	0.00	0.00	0.00	0.00	0.00	0.00	0.00	0.00	0.00	0.00
		River	0.01	0.01	0.01	0.00	0.00	0.01	0.00	0.04	0.00	0.00	0.00	0.00	0.00	0.01
		Land-based	0.26	0.41	0.44	0.04	0.03	0.27	0.12	1.32	0.05	0.03	0.01	0.01	0.00	0.32
GHG emission	Stage 1	Raw material extraction	8.35	10.21	23.36	1.21	1.48	17.32	3.70	46.57	3.64	1.06	0.29	0.70	0.06	17.53
		monomer production	8.23	10.06	23.03	3.35	4.10	27.74	9.29	163.71	3.04	3.28	0.36	2.28	0.14	17.28
		Polymer production	6.69	8.18	18.50	0.62	0.76	13.83	3.40	63.69	3.14	1.78	0.20	0.60	0.07	15.14
	Stage 2	Primary manufacture	8.30	8.16	17.80	0.63	2.68	7.02	6.51	36.98	4.56	2.04	0.62	0.61	0.07	11.51
		Terminal manufacture	1.63	4.00	5.97	0.06	0.17	4.85	1.00	34.21	1.07	0.07	0.02	0.01	0.00	3.85
	Stage 4	Recovery	0.52	0.73	0.87	0.04	0.03	0.32	0.17	3.12	0.09	0.03	0.01	0.02	0.00	0.45
		Incineration	4.79	7.50	7.98	0.65	0.49	4.93	2.11	24.06	0.93	0.93	0.17	0.26	0.05	5.85
		Landfill	0.19	0.30	0.32	0.03	0.02	0.21	0.09	0.90	0.04	0.03	0.01	0.01	0.00	0.24
		Material substitution	-1.84	-2.58	-2.96	-0.27	-0.20	-1.37	-1.03	-40.80	-2.61	-0.26	-0.04	-0.19	-0.03	-3.48
		Energy substitution	-1.84	-2.87	-3.06	-0.25	-0.19	-1.89	-0.81	-9.22	-0.36	-0.23	-0.07	-0.10	-0.02	-2.24

Leakage: 1.4, 1.2, 1.0, 0.8, 0.6, 0.4, 0.2, 0.0

GHG: 180.0, 150.0, 120.0, 90.0, 60.0, 30.0, 0.0, −30.0, −60.0

b

	HDPE	LDPE	PP	EPS	GPPS	PVC	ABS	PET	PA	PC	POM	PBT	PPS	Others
Packaging	0.14	0.28	0.30	0.03	0.01	0.06	0.00	0.21	0.00	0.02	0.00	0.00	0.00	0.09
Agriculture	0.01	0.10	0.00	0.00	0.00	0.00	0.00	0.00	0.00	0.00	0.00	0.00	0.00	0.06
B&C	0.00	0.00	0.01	0.00	0.00	0.00	0.00	0.00	0.00	0.00	0.00	0.00	0.00	0.00
Medical	0.03	0.00	0.00	0.00	0.00	0.01	0.00	0.00	0.00	0.00	0.00	0.00	0.00	0.04
Transportation	0.01	0.00	0.03	0.00	0.00	0.03	0.00	0.00	0.01	0.00	0.00	0.00	0.00	0.03
E&E	0.01	0.00	0.03	0.00	0.01	0.02	0.06	0.01	0.03	0.00	0.00	0.00	0.00	0.07
Consumer	0.05	0.01	0.03	0.00	0.01	0.12	0.05	0.00	0.00	0.01	0.01	0.00	0.00	0.01
Electric power communication	0.00	0.00	0.00	0.00	0.00	0.03	0.00	0.00	0.00	0.00	0.00	0.00	0.00	0.00
Textile	0.00	0.00	0.04	0.00	0.00	0.00	0.00	1.10	0.00	0.00	0.00	0.01	0.00	0.01
Industry Machinery	0.00	0.00	0.00	0.00	0.00	0.00	0.00	0.00	0.01	0.00	0.00	0.00	0.00	0.01

Leakage (Mt): 1.2, 1.0, 0.8, 0.6, 0.4, 0.2, 0.0

Supplementary Figure 6. Detailed environmental leakage and GHG emissions by type. a, Environmental leakage and GHG emissions by type of plastic in four stages in 2021. b, Mismanagement of different types of plastic in various industries in 2021.

Supplementary Method 5. Scenario analysis

Population and GDP

We predict the per capita plastic inventory based on per capita GDP[44], combining population forecasts[45] to obtain historical inventory volumes by plastic type and industry. Then, we reverse-calculate the annual production, consumption, disposal, and inventory volumes under the business-as-usual scenario. The historical per capita GDP and population predictions for China are shown in the table below.

Supplementary Table 27. Historical and projected data on China's population and GDP.

Year	Population/ (10,000 people)	GDP/ dollars (billion yuan)	Per capita GDP/ (10,000 yuan per person)	GDP growth rate(middle)
1992	117761.08	4269.2	362.5	
1993	119001.59	4447.3	373.7	4.17%
1994	120169.52	5643.2	469.6	26.89%
1995	121287.82	7344.8	605.6	30.15%
1996	122341.07	8637.5	706.0	17.60%
1997	123318.70	9616.0	779.8	11.33%
1998	124241.59	10290.6	828.3	7.02%
1999	125125.63	10940.1	874.3	6.31%
2000	125961.02	12113.3	961.7	10.72%
2001	126858.80	13394.0	1055.8	10.57%
2002	127689.12	14705.6	1151.7	9.79%
2003	128496.11	16602.8	1292.1	12.90%
2004	129278.57	19553.5	1512.5	17.77%
2005	130084.78	22859.6	1757.3	16.91%
2006	130892.74	27521.2	2102.6	20.39%
2007	131724.58	35503.3	2695.3	29.00%
2008	132578.07	45943.4	3465.4	29.41%
2009	133455.36	51016.9	3822.8	11.04%
2010	134369.76	60871.9	4530.2	19.32%
2011	135268.52	75515.5	5582.6	24.06%
2012	136150.58	85321.9	6266.7	12.99%
2013	137161.58	95704.7	6977.5	12.17%
2014	138058.48	104756.2	7587.8	9.46%
2015	138979.46	110615.7	7959.1	5.59%
2016	139763.63	112333.1	8037.4	1.55%
2017	140614.30	123104.9	8754.8	9.59%
2018	141440.89	138949.1	9823.8	12.87%
2019	141973.00	142799.7	10058.2	2.77%

2020	142399.80	146877.4	10314.4	2.86%
2021	142586.15	178204.6	12498.0	21.33%
2022	142592.54	179631.7	12597.6	0.80%
2023	142584.93	190679.1	13373.0	6.15%
2024	142549.34	202167.5	14182.3	6.03%
2025	142486.41	214115.6	15027.1	5.91%
2026	142389.97	226502.2	15907.2	5.79%
2027	142261.07	239197.6	16814.0	5.61%
2028	142100.74	252174.1	17746.1	5.43%
2029	141908.37	265400.6	18702.3	5.25%
2030	141686.55	278843.1	19680.3	5.07%
2031	141434.63	292464.6	20678.4	4.89%
2032	141154.57	306473.7	21711.9	4.79%
2033	140848.00	320862.6	22780.8	4.70%
2034	140514.08	335622.3	23885.3	4.60%
2035	140148.89	350742.1	25026.4	4.51%
2036	139760.61	366227.3	26203.9	4.42%
2037	139348.73	382030.1	27415.4	4.32%
2038	138918.95	398151.7	28660.7	4.22%
2039	138471.45	414595.4	29940.9	4.13%
2040	138001.98	431324.3	31254.9	4.04%
2041	137509.41	448296.9	32601.2	3.94%
2042	136995.07	465511.5	33980.2	3.84%
2043	136458.35	482968.2	35393.1	3.75%
2044	135889.10	500620.7	36840.4	3.66%
2045	135293.47	518417.8	38318.0	3.56%
2046	134657.91	536380.9	39832.9	3.47%
2047	133984.13	554457.0	41382.3	3.37%
2048	133268.45	572615.4	42967.1	3.28%
2049	132506.27	590824.6	44588.4	3.18%
2050	131694.57	609051.6	46247.3	3.09%
2051	130832.70	627541.4	47965.2	3.04%
2052	129918.62	646288.9	49745.7	2.99%
2053	128957.60	665288.9	51589.7	2.94%
2054	127950.38	684535.7	53500.1	2.89%
2055	126898.57	704023.6	55479.2	2.85%
2056	125803.94	723747.1	57529.8	2.80%
2057	124670.46	743699.9	59653.3	2.76%
2058	123505.54	763876.1	61849.5	2.71%
2059	122319.69	784269.4	64116.4	2.67%

2060	121111.90	804873.5	66457.0	2.63%

Inventory volume forecast

Drewniok, M. P. Et al.[31] estimated the demand and waste generation for plastics in the United Kingdom based on the saturation of plastic stocks in the country. We employed the Gompertz model to simulate the per capita inventory volume in different industries according to per capita GDP.

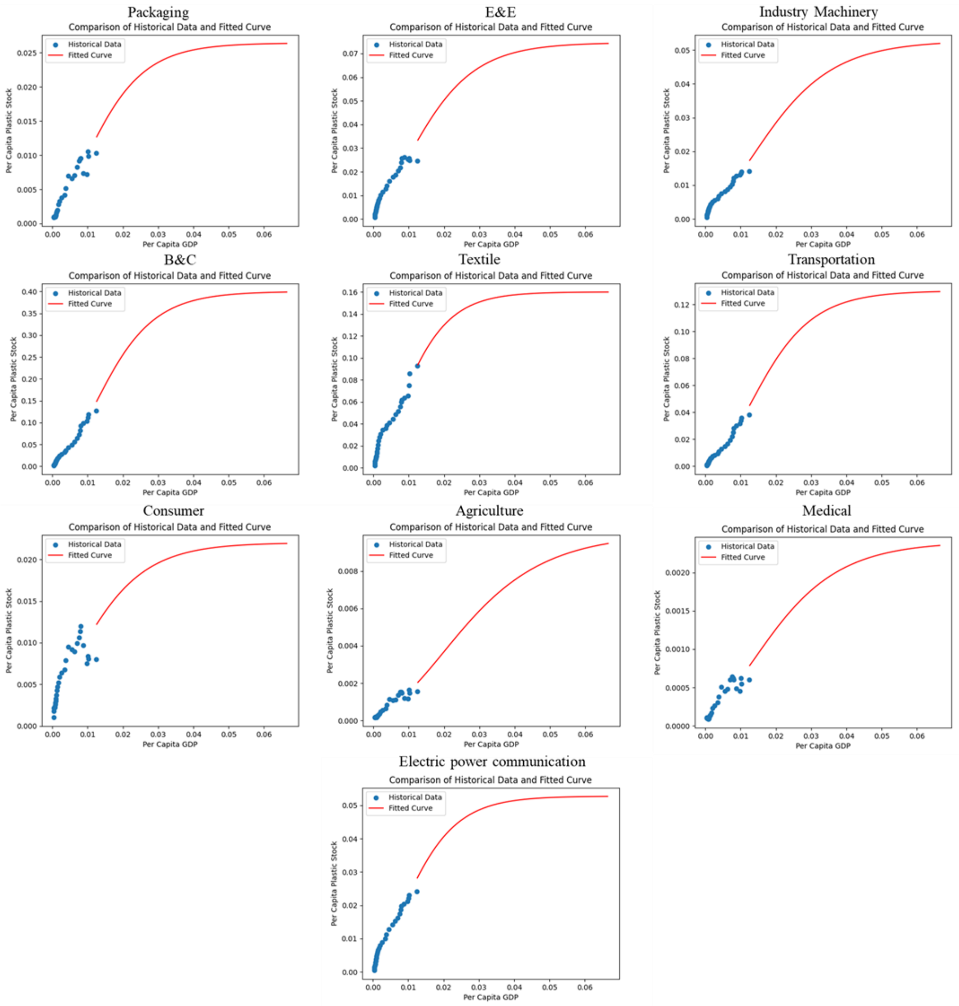

Supplementary Figure 7. Comparison of historical data and fitted curves across various industries.

Prediction of production, consumption, and stocks.

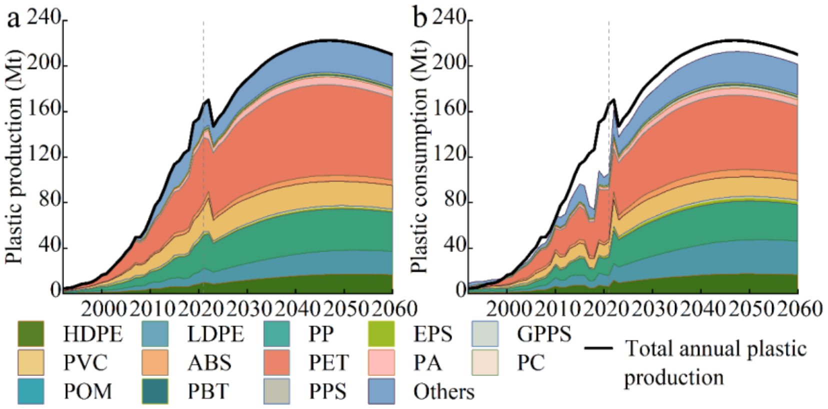

Supplementary Figure 8. Production (a) and Consumption (b) from 1992 to 2060.

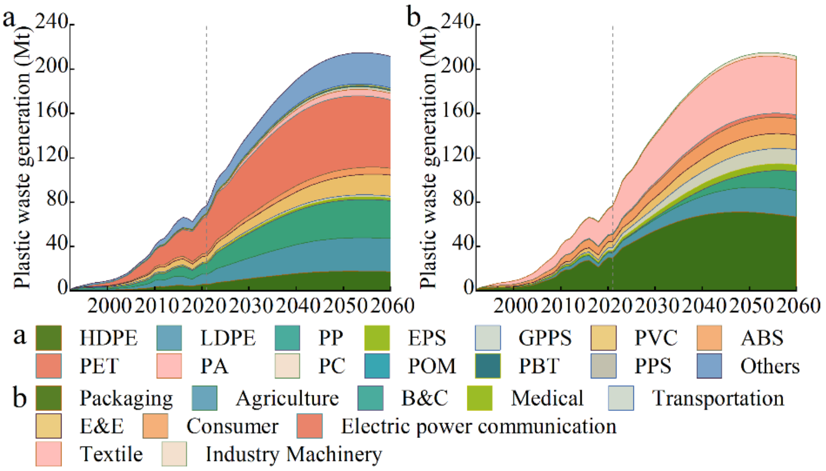

Supplementary Figure 9. Plastic waste generated by plastic type (a) and plastic waste generated by consumption sectors (b) from 1992 to 2060.

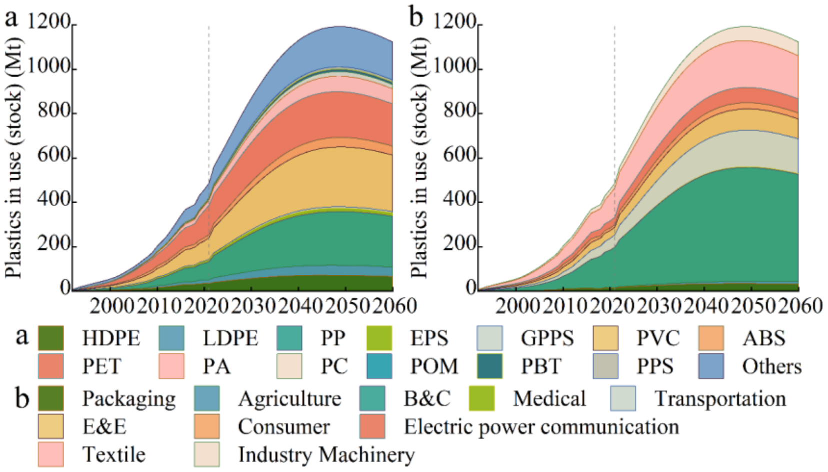

Supplementary Figure 10. Plastic inventory in use generated by plastic type (a) and plastic inventory in use generated by consumption sectors (b) from 1992 to

2060.

Supplementary Method 6. Scenario construction

Supplementary Table 28. Scenario construction.

Categories of system interventions conceptualized for scenario building.			
	Environmental leakage assumptions	GHG emissions assumptions	Description/Example
Baseline scenario	Business-as-usual (BAU)	BAU	None
Single measure	-	Reform of the energy structure	1. Renewable energy (energy substitution).
	Design optimization for reduction	Design optimization for reduction	2. Promotion of green design in plastic products.
	Reduction in demand	Reduction in demand	3. Diminishing the overall plastic in the system (curbing the growth of demand).
	Reduction through substitution	Reduction through substitution	4. Replacement of plastic with alternative materials, such as bio-based plastics.
	Collection		5. Enhancement of collection capacity to reduce post-consumer leakage.
	Mechanical recycling	Mechanical recycling	6. Scaling of sorting and mechanical recycling capabilities (for recycling and reuse).
	Chemical recycling	Chemical recycling	7. Expansion of chemical conversion capabilities (for recycling and repurposing).
	Incineration	Incineration	8. Mitigation of post-collection leakage (for recycling and reuse).
	Landfilling	Landfilling	9. Mitigation of post-collection leakage (for recycling and reuse).
Composite measures	Reduction and substitution	Reduction and substitution	1-4.
	Recycling	Recycling	6-7.
	Collect and dispose	Collect and dispose	5, 8-9.
	System change	System change	Integration of strategies 1-9.

BAU

When the production, consumption, and inventory levels are set at a constant value of 7.3, all other parameters will remain unchanged. Specifically, factors including the energy structure, leakage parameters, and plastic treatment rates will continue to be based on the benchmark data from 2021.

	S1	S2	S3	S4	Total	Aq.	Ter.	S1	S2	S3	S4	Net.	Emiss.	Offset
**HDPE**	0.18	0.30	0.34	0.78	1.60	0.38	1.22	36	13	0	4	53	64	-11
**LDPE**	0.22	0.37	0.04	1.36	1.99	0.10	1.89	44	26	0	9	78	97	-19
**PP**	0.36	0.52	0.05	1.62	2.55	0.12	2.43	72	28	0	12	113	133	-20
**EPS**	0.01	0.02	0.01	0.12	0.16	0.01	0.15	4	1	0	1	6	7	-2
**GPPS**	0.02	0.03	0.01	0.09	0.15	0.01	0.13	5	2	0	1	8	9	-1
**PVC**	0.22	0.30	0.01	1.00	1.53	0.05	1.48	54	11	0	9	74	84	-10
**ABS**	0.05	0.09	0.00	0.30	0.44	0.01	0.42	17	8	0	1	26	31	-5
**PET**	0.76	1.05	0.05	2.54	4.41	0.18	4.23	332	82	0	-34	381	468	-87
**PA**	0.06	0.09	0.01	0.28	0.45	0.02	0.42	55	8	0	-8	54	66	-12
**PC**	0.01	0.03	0.00	0.13	0.17	0.01	0.17	6	2	0	1	9	10	-1
**POM**	0.00	0.01	0.00	0.03	0.04	0.00	0.04	1	1	0	0	2	2	-0
**PBT**	0.01	0.01	0.00	0.04	0.07	0.00	0.06	4	1	0	0	5	6	-1
**PPS**	0.01	0.02	0.00	0.05	0.08	0.00	0.08	3	1	0	0	4	5	-1
**Others**	0.29	0.44	0.06	1.41	2.20	0.12	2.08	60	21	0	5	86	108	-23

Leakage (Mt): 0.0 1.0 2.0 3.0 4.0 5.0

GHG (Mt CO_2-eq): −200 0 200 400 600

Supplementary Figure 11. Environmental leakage and GHG emissions of 14 plastics under BAU in 2060. S1, S2, S3 and S4 represent production, T&M, use and disposal, respectively. Total refers to the total amount of environmental leakage. Aq. refers to aquatic environment leakage. Ter. refers to terrestrial environmental leakage. Net. refers to net GHG emissions. Emiss. refers to total GHG emissions. Offset refers to GHG offsets from recycling substitution and incineration for electricity generation.

Reform of the energy structure

The measures for transforming the energy structure throughout the entire lifecycle of plastics are primarily aimed at reducing GHG emissions. Through a holistic strategy and actions, spanning from raw material production to plastic manufacturing, usage, and the recycling and disposal of waste, these measures effectively curtail carbon emissions to confront the challenges posed by climate change. The proportions of associated energy and electricity consumption are supplementary table 29-31.

Supplementary Table 29. Estimated Share of Power system Energy consumption in China (%).

	Hydropower	Coal Power	Nuclear Power	Wind Power	Photovoltaic
2022	0.18	0.67	0.05	0.06	0.03
2023	0.18	0.65	0.06	0.07	0.04
2024	0.19	0.64	0.06	0.07	0.04
2025	0.19	0.63	0.06	0.08	0.05
2026	0.19	0.61	0.06	0.08	0.05
2027	0.19	0.60	0.07	0.09	0.06
2028	0.19	0.58	0.07	0.09	0.06
2029	0.19	0.57	0.07	0.10	0.06
2030	0.20	0.56	0.07	0.10	0.07
2031	0.20	0.54	0.08	0.11	0.07
2032	0.20	0.53	0.08	0.11	0.08
2033	0.20	0.52	0.08	0.12	0.08
2034	0.20	0.50	0.08	0.12	0.09
2035	0.21	0.49	0.09	0.13	0.09
2036	0.21	0.48	0.09	0.13	0.10
2037	0.21	0.46	0.09	0.14	0.10
2038	0.21	0.45	0.09	0.14	0.10
2039	0.21	0.44	0.10	0.15	0.11
2040	0.21	0.42	0.10	0.15	0.11
2041	0.22	0.41	0.10	0.16	0.12
2042	0.22	0.39	0.10	0.16	0.12
2043	0.22	0.38	0.11	0.17	0.13
2044	0.22	0.37	0.11	0.17	0.13
2045	0.22	0.35	0.11	0.18	0.13
2046	0.22	0.34	0.11	0.18	0.14
2047	0.23	0.33	0.12	0.19	0.14
2048	0.23	0.31	0.12	0.19	0.15

2049	0.23	0.30	0.12	0.20	0.15
2050	0.23	0.29	0.12	0.20	0.16
2051	0.23	0.27	0.13	0.21	0.16
2052	0.24	0.26	0.13	0.21	0.17
2053	0.24	0.25	0.13	0.22	0.17
2054	0.24	0.23	0.13	0.22	0.17
2055	0.24	0.22	0.14	0.23	0.18
2056	0.24	0.20	0.14	0.23	0.18
2057	0.24	0.19	0.14	0.24	0.19
2058	0.25	0.18	0.14	0.24	0.19
2059	0.25	0.16	0.15	0.25	0.20
2060	0.25	0.15	0.15	0.25	0.20

Supplementary Table 30. Estimated National Energy Consumption Ratio for Industrial Steam Production (%).

	Coal	Natural gas	Solid waste
2022	0.69	0.30	0.01
2023	0.67	0.30	0.03
2024	0.66	0.30	0.04
2025	0.64	0.30	0.06
2026	0.63	0.30	0.07
2027	0.62	0.30	0.08
2028	0.60	0.30	0.10
2029	0.59	0.30	0.11
2030	0.57	0.30	0.13
2031	0.56	0.30	0.14
2032	0.54	0.30	0.16
2033	0.53	0.30	0.17
2034	0.52	0.30	0.18
2035	0.50	0.30	0.20
2036	0.49	0.30	0.21
2037	0.47	0.30	0.23
2038	0.46	0.30	0.24
2039	0.45	0.30	0.25
2040	0.43	0.30	0.27
2041	0.42	0.30	0.28
2042	0.40	0.30	0.30
2043	0.39	0.30	0.31
2044	0.38	0.30	0.32
2045	0.36	0.30	0.34

2046	0.35	0.30	0.35
2047	0.33	0.30	0.37
2048	0.32	0.30	0.38
2049	0.31	0.30	0.39
2050	0.29	0.30	0.41
2051	0.28	0.30	0.42
2052	0.26	0.30	0.44
2053	0.25	0.30	0.45
2054	0.23	0.30	0.47
2055	0.22	0.30	0.48
2056	0.21	0.30	0.49
2057	0.19	0.30	0.51
2058	0.18	0.30	0.52
2059	0.16	0.30	0.54
2060	0.15	0.30	0.55

Supplementary Table 31. Estimates of China's fuel consumption ratio (%).

	Coal	Natural gas	Solid waste	Primary power and others
2022	0.55	0.18	0.09	0.18
2023	0.54	0.18	0.09	0.19
2024	0.52	0.18	0.09	0.20
2025	0.51	0.18	0.09	0.22
2026	0.50	0.17	0.10	0.23
2027	0.49	0.17	0.10	0.24
2028	0.48	0.17	0.10	0.25
2029	0.46	0.17	0.10	0.27
2030	0.45	0.17	0.10	0.28
2031	0.44	0.16	0.10	0.29
2032	0.43	0.16	0.11	0.30
2033	0.42	0.16	0.11	0.32
2034	0.41	0.16	0.11	0.33
2035	0.39	0.16	0.11	0.34
2036	0.38	0.15	0.11	0.35
2037	0.37	0.15	0.11	0.37
2038	0.36	0.15	0.12	0.38
2039	0.35	0.15	0.12	0.39
2040	0.34	0.14	0.12	0.40
2041	0.32	0.14	0.12	0.41
2042	0.31	0.14	0.12	0.43
2043	0.30	0.14	0.12	0.44

2044	0.29	0.14	0.12	0.45
2045	0.28	0.13	0.13	0.46
2046	0.26	0.13	0.13	0.48
2047	0.25	0.13	0.13	0.49
2048	0.24	0.13	0.13	0.50
2049	0.23	0.12	0.13	0.51
2050	0.22	0.12	0.13	0.53
2051	0.21	0.12	0.14	0.54
2052	0.19	0.12	0.14	0.55
2053	0.18	0.12	0.14	0.56
2054	0.17	0.11	0.14	0.58
2055	0.16	0.11	0.14	0.59
2056	0.15	0.11	0.14	0.60
2057	0.14	0.11	0.15	0.61
2058	0.12	0.10	0.15	0.63
2059	0.11	0.10	0.15	0.64
2060	0.10	0.10	0.15	0.65

	S1	S2	S3	S4	Total	Aq.	Ter.	S1	S2	S3	S4	Net.	Emiss.	Offset
HDPE	0.18	0.30	0.34	0.78	1.60	0.38	1.22	20	3	0	9	32	37	-5
LDPE	0.22	0.37	0.04	1.36	1.99	0.10	1.89	24	6	0	18	48	56	-8
PP	0.36	0.52	0.05	1.62	2.55	0.12	2.43	40	7	0	22	69	77	-8
EPS	0.01	0.02	0.01	0.12	0.16	0.01	0.15	2	0	0	2	4	4	-1
GPPS	0.02	0.03	0.01	0.09	0.15	0.01	0.13	3	0	0	1	4	5	-0
PVC	0.22	0.30	0.01	1.00	1.53	0.05	1.48	28	3	0	15	46	49	-4
ABS	0.05	0.09	0.00	0.30	0.44	0.01	0.42	8	2	0	4	14	16	-2
PET	0.76	1.05	0.05	2.54	4.41	0.18	4.23	139	21	0	10	170	210	-40
PA	0.06	0.09	0.01	0.28	0.45	0.02	0.42	22	2	0	-1	23	27	-4
PC	0.01	0.03	0.00	0.13	0.17	0.01	0.17	2	1	0	2	5	5	-1
POM	0.00	0.01	0.00	0.03	0.04	0.00	0.04	0	0	0	0	1	1	-0
PBT	0.01	0.01	0.00	0.04	0.07	0.00	0.06	2	0	0	0	2	3	-0
PPS	0.01	0.02	0.00	0.05	0.08	0.00	0.08	1	0	0	0	2	3	-0
Others	0.29	0.44	0.06	1.41	2.20	0.12	2.08	33	5	0	17	55	64	-9

Leakage (Mt): 0.0 1.0 2.0 3.0 4.0 5.0
GHG (Mt CO_2-eq): −200 0 200 400 600

Supplementary Figure 12. Environmental leakage and GHG emissions of 14 plastics under Reform of the energy structure in 2060. S1, S2, S3 and S4 represent

production, T&M, use and disposal, respectively. Total refers to the total amount of environmental leakage. Aq. refers to aquatic environment leakage. Ter. refers to terrestrial environmental leakage. Net. refers to net GHG emissions. Emiss. refers to total GHG emissions. Offset refers to GHG offsets from recycling substitution and incineration for electricity generation.

Reduction in demand

In the current policies, the reduction in demand primarily focuses on single-use plastic products. The main entities directly implementing this are operators in commodity retail, e-commerce, catering, accommodation, as well as platform enterprises in e-commerce, food delivery, and express delivery services. We have set a target of a 30% reduction in demand for the packaging industry by 2060.

	Leakage (Mt)							GHG (Mt CO_2-eq)						
	S1	S2	S3	S4	Total	Aq.	Ter.	S1	S2	S3	S4	Net.	Emiss.	Offset
HDPE	0.15	0.26	0.34	0.68	1.43	0.38	1.06	28	12	0	4	43	53	-9
LDPE	0.18	0.32	0.04	1.15	1.69	0.09	1.60	34	23	0	8	64	81	-16
PP	0.31	0.45	0.05	1.40	2.20	0.11	2.09	59	24	0	11	94	111	-17
EPS	0.01	0.02	0.01	0.09	0.13	0.01	0.12	3	1	0	1	5	6	-1
GPPS	0.01	0.02	0.01	0.08	0.13	0.01	0.12	4	2	0	1	7	8	-1
PVC	0.21	0.28	0.01	0.96	1.46	0.04	1.42	50	10	0	9	69	79	-10
ABS	0.05	0.09	0.00	0.30	0.44	0.01	0.42	15	8	0	1	25	29	-5
PET	0.72	0.99	0.05	2.48	4.23	0.18	4.06	306	78	0	-25	359	435	-76
PA	0.06	0.09	0.01	0.28	0.44	0.02	0.42	54	8	0	-8	53	65	-12
PC	0.01	0.02	0.00	0.12	0.15	0.00	0.15	5	2	0	1	7	9	-1
POM	0.00	0.01	0.00	0.03	0.05	0.00	0.04	1	1	0	0	1	2	-0
PBT	0.01	0.01	0.00	0.04	0.07	0.00	0.06	4	1	0	0	5	6	-1
PPS	0.01	0.02	0.00	0.05	0.08	0.00	0.08	3	1	0	0	4	5	-1
Others	0.27	0.41	0.06	1.31	2.04	0.11	1.93	53	20	0	4	77	98	-21

Leakage (Mt) color scale: 0.0, 1.0, 2.0, 3.0, 4.0, 5.0; GHG (Mt CO_2-eq) color scale: −200, 0, 200, 400, 600

Supplementary Figure 13. Environmental leakage and GHG emissions of 14 plastics under Reduction in demand in 2060. S1, S2, S3 and S4 represent production, T&M, use and disposal, respectively. Total refers to the total amount of

environmental leakage. Aq. refers to aquatic environment leakage. Ter. refers to terrestrial environmental leakage. Net. refers to net GHG emissions. Emiss. refers to total GHG emissions. Offset refers to GHG offsets from recycling substitution and incineration for electricity generation.

Design optimization for reduction

The strategy of plastic design reduction aims to decrease the usage of plastic by optimizing product design, thereby achieving the goals of reducing resource consumption and minimizing environmental impact. Lightweight design entails reducing the thickness or volume of plastic components to decrease weight without compromising functionality. Simplified design involves the removal of unnecessary decorations or features, streamlining the product structure to reduce the amount of plastic used. Through these measures, the utilization of plastic can be curtailed at the source, thereby mitigating the environmental impact of plastic, particularly by reducing GHG emissions generated throughout its lifecycle.

In order to estimate the reduction in plastic, we used Unilever's explicit target. Unilever's reduction of 100,000 t of plastic packaging by 2025 over its current volume of 700,000 t translates into a reduction of 14%[3,46]. Unilever's reduction of 100,000 t of plastic packaging by 2025 over its current volume of 700,000 t translates into a reduction of 14%[3,46]. Unilever's target is seen as ambitious among the signatories.

In September 2021, the Chinese government issued the "Fourteenth Five-Year Plan" Action Plan for Plastic Pollution Control[47], focusing on green design, usage reduction, and substitution, with a particular emphasis on single-use plastic products. The production of environmentally and health-hazardous products such as ultra-thin shopping bags, polyethylene agricultural mulch films, and daily chemical products containing plastic microbeads has been prohibited. The resource environmental impact throughout the entire lifecycle of products such as bamboo and wood products, paper products, and biodegradable plastic products has been fully considered. Single-use plastic shopping bags are predominantly used in the packaging industry, plastic microbeads are mainly found in daily chemical products, and agricultural mulch films are concentrated in agriculture. Therefore, in the scenario setting of this paper, we have constructed it based on the aforementioned content.

Design Reduction: By 2060, a 30% reduction in design is targeted for the packaging industry. The production of daily chemical products containing plastic microbeads and polyethylene agricultural mulch films with a thickness less than 0.01 millimeters will be prohibited starting from the year of the scenario setting.

The following are the main contents of source reduction for plastic production and usage as outlined in China's "Fourteenth Five-Year Plan" Action Plan for Plastic Pollution Control.

Actively promote green design for plastic products. Focusing on single-use

plastic products, develop relevant standards for green design, optimize product structural design, reduce the complexity of product material design, and enhance the recyclability of plastic products. Prohibit the production of ultra-thin plastic shopping bags with a thickness less than 0.025 millimeters, polyethylene agricultural mulch films with a thickness less than 0.01 millimeters, and daily chemical products containing plastic microbeads, which are harmful to the environment and human health. Strengthen the implementation and promotion of standards limiting excessive packaging of commodities and enhance law enforcement supervision over excessive packaging.

Continuously promote the reduction in the use of single-use plastic products. Implement national regulations prohibiting, restricting the sale, and use of certain plastic products. Develop the "Management Methods for the Use and Reporting of Single-Use Plastic Products" to establish a comprehensive system for reporting the use and recycling of single-use plastic products, and supervise and guide business operators in retail, e-commerce, catering, and accommodation to fulfill their responsibilities. Urge and guide e-commerce, food delivery platforms, and express delivery companies to formulate rules for reducing the use of single-use plastic products. Publish a recommended directory of green packaging products, promote the integration of products with express packaging, and encourage the direct shipment of e-commerce packages in original packaging, significantly reducing secondary packaging in the delivery process of e-commerce goods. Conduct pilot projects for the large-scale application of recyclable express packaging. Promote the shared use of standardized logistics turnover boxes nationwide. Accelerate the implementation of the green product certification system for express packaging. Play a leading role in public institutions by reducing the use of single-use plastic products. Explore pilot projects replacing plastic bottled water with direct drinking water machines in reception and training venues of government agencies. Strengthen public education and science popularization to guide the public in developing green consumption habits, reducing the consumption of single-use plastic products, and consciously fulfilling the obligation of sorting and disposing of household waste.

Scientifically and prudently promote alternative products to plastic. Fully consider the resource environmental impact throughout the entire lifecycle of bamboo and wood products, paper products, and biodegradable plastic products, and improve the quality and food safety standards for related products. Conduct research on the degradation mechanisms and impacts of different types of biodegradable plastics to scientifically assess their environmental safety and controllability. Perfect the standard system, issue standards for biodegradable plastics, regulate application fields, and clarify degradation conditions and disposal methods. Increase efforts in tackling key technical challenges and transforming achievements in biodegradable plastics to continuously improve product quality and performance and reduce application costs. Promote the orderly development of the biodegradable plastics industry, guide rational industry layout, and prevent the blind expansion of production capacity. Accelerate

scientific research and the promotion and application of fully biodegradable agricultural films. Strengthen the construction of biodegradable plastic testing capabilities, strictly investigate and penalize false labeling and pseudo-labeling of biodegradable plastics, and standardize industry order.

	S1	S2	S3	S4	Total	Aq.	Ter.	S1	S2	S3	S4	Net.	Emiss.	Offset
**HDPE**	0.14	0.06	0.00	0.68	0.88	0.03	0.85	28	12	0	4	43	53	-9
**LDPE**	0.17	0.07	0.00	1.15	1.39	0.04	1.35	34	23	0	8	64	81	-16
**PP**	0.07	0.10	0.01	1.40	1.57	0.05	1.52	59	24	0	11	94	111	-17
**EPS**	0.00	0.00	0.00	0.09	0.10	0.00	0.10	3	1	0	1	5	6	-1
**GPPS**	0.00	0.01	0.00	0.08	0.09	0.00	0.09	4	2	0	1	7	8	-1
**PVC**	0.05	0.06	0.01	0.96	1.08	0.04	1.05	50	10	0	9	69	79	-10
**ABS**	0.01	0.02	0.00	0.30	0.33	0.01	0.32	15	8	0	1	25	29	-5
**PET**	0.16	0.22	0.05	2.48	2.91	0.15	2.76	306	78	0	-25	359	435	-76
**PA**	0.01	0.02	0.00	0.28	0.32	0.01	0.30	54	8	0	-8	53	65	-12
**PC**	0.00	0.01	0.00	0.12	0.12	0.00	0.12	5	2	0	1	7	9	-1
**POM**	0.00	0.00	0.00	0.03	0.04	0.00	0.03	1	1	0	0	1	2	-0
**PBT**	0.00	0.00	0.00	0.04	0.05	0.00	0.04	4	1	0	0	5	6	-1
**PPS**	0.00	0.00	0.00	0.05	0.06	0.00	0.06	3	1	0	0	4	5	-1
**Others**	0.06	0.09	0.01	1.31	1.47	0.05	1.41	53	20	0	4	77	98	-21

Leakage (Mt): 0.0 1.0 2.0 3.0 4.0 5.0

GHG (Mt CO_2-eq): −200 0 200 400 600

Supplementary Figure 14. Environmental leakage and GHG emissions of 14 plastics under Design optimization for reduction in 2060. S1, S2, S3 and S4 represent production, T&M, use and disposal, respectively. Total refers to the total amount of environmental leakage. Aq. refers to aquatic environment leakage. Ter. refers to terrestrial environmental leakage. Net. refers to net GHG emissions. Emiss. refers to total GHG emissions. Offset refers to GHG offsets from recycling substitution and incineration for electricity generation.

Reduction through substitution

Fully consider bamboo and wood products, paper products, glass, and biodegradable plastic products as alternatives. We assume that by 2060, these alternatives will replace 30% of the market share in the packaging industry, with an equal distribution among the four types of substitute products.

Supplementary Table 32. Carbon emission per unit of substitute product (t CO_2 -eq per t)[37].

Substitute	Biodegradable plastics	glass	bamboo	paper cups
GHG emissions	3.85	1.3	0.03	2.53

	S1	S2	S3	S4	Total	Aq.	Ter.	S1	S2	S3	S4	Net.	Emiss.	Offset
HDPE	0.15	0.26	0.34	0.68	1.43	0.38	1.06	28	16	0	4	48	57	-9
LDPE	0.18	0.32	0.04	1.15	1.69	0.09	1.60	34	31	0	8	73	89	-16
PP	0.31	0.45	0.05	1.40	2.20	0.11	2.09	59	34	0	11	104	121	-17
EPS	0.01	0.02	0.01	0.09	0.13	0.01	0.12	3	2	0	1	6	7	-1
GPPS	0.01	0.02	0.01	0.08	0.13	0.01	0.12	4	2	0	1	7	8	-1
PVC	0.21	0.28	0.01	0.96	1.46	0.04	1.42	50	12	0	9	71	80	-10
ABS	0.05	0.09	0.00	0.30	0.44	0.01	0.42	15	8	0	1	25	29	-5
PET	0.72	0.99	0.05	2.48	4.23	0.18	4.06	306	87	0	-25	368	444	-76
PA	0.06	0.09	0.01	0.28	0.44	0.02	0.42	54	8	0	-8	53	65	-12
PC	0.01	0.02	0.00	0.12	0.15	0.00	0.15	5	2	0	1	8	9	-1
POM	0.00	0.01	0.00	0.03	0.05	0.00	0.04	1	1	0	0	1	2	-0
PBT	0.01	0.01	0.00	0.04	0.07	0.00	0.06	4	1	0	0	5	6	-1
PPS	0.01	0.02	0.00	0.05	0.08	0.00	0.08	3	1	0	0	4	5	-1
Others	0.27	0.41	0.06	1.31	2.04	0.11	1.93	53	24	0	4	81	102	-21

Leakage (Mt): 0.0 1.0 2.0 3.0 4.0 5.0

GHG (Mt CO_2-eq): −200 0 200 400 600

Supplementary Figure 15. Environmental leakage and GHG emissions of 14 plastics under Reduction through substitution in 2060. S1, S2, S3 and S4 represent production, T&M, use and disposal, respectively. Total refers to the total amount of

environmental leakage. Aq. refers to aquatic environment leakage. Ter. refers to terrestrial environmental leakage. Net. refers to net GHG emissions. Emiss. refers to total GHG emissions. Offset refers to GHG offsets from recycling substitution and incineration for electricity generation.

Collection

Enhancing the collection efficiency at the plastic disposal stage is a crucial measure for reducing the mismanagement of plastics. China attaches great importance to the control of plastic pollution, from the “Environmental Protection Law of the People’s Republic of China” enacted in 1989, to the “Opinions on Further Strengthening the Control of Plastic Pollution” issued in 2020, and the “Fourteenth Five-Year Plan” Action Plan for Plastic Pollution Control in 2021, the Chinese government has been actively promoting the lifecycle management of plastic pollution. The rate of mismanagement of plastics in China has decreased from 72% in 1992 to approximately 6%. Here, we assume that by 2060, the collection rate for safe end-of-life disposal will reach 99%, with the rate of mismanagement reduced to 1%.

	S1	S2	S3	S4	Total	Aq.	Ter.	S1	S2	S3	S4	Net.	Emiss.	Offset
**HDPE**	0.18	0.30	0.32	0.25	1.05	0.34	0.71	36	13	0	7	55	68	-12
**LDPE**	0.22	0.37	0.01	0.43	1.02	0.04	0.99	44	26	0	14	83	105	-22
**PP**	0.36	0.52	0.02	0.49	1.39	0.05	1.35	72	28	0	18	119	143	-24
**EPS**	0.01	0.02	0.00	0.03	0.07	0.00	0.07	4	1	0	1	6	8	-2
**GPPS**	0.02	0.03	0.01	0.03	0.08	0.01	0.07	5	2	0	1	8	9	-1
**PVC**	0.22	0.30	0.01	0.28	0.81	0.02	0.79	54	11	0	13	78	91	-13
**ABS**	0.05	0.09	0.00	0.09	0.23	0.01	0.22	17	8	0	2	27	33	-6
**PET**	0.76	1.05	0.05	0.86	2.73	0.12	2.60	332	82	0	-27	387	482	-94
**PA**	0.06	0.09	0.01	0.08	0.25	0.02	0.23	55	8	0	-8	54	67	-13
**PC**	0.01	0.03	0.00	0.03	0.08	0.00	0.08	6	2	0	1	9	11	-2
**POM**	0.00	0.01	0.00	0.01	0.02	0.00	0.02	1	1	0	0	2	2	-0
**PBT**	0.01	0.01	0.00	0.01	0.04	0.00	0.04	4	1	0	0	5	6	-1
**PPS**	0.01	0.02	0.00	0.02	0.05	0.00	0.04	3	1	0	0	4	5	-1
**Others**	0.29	0.44	0.01	0.42	1.15	0.03	1.12	60	21	0	9	90	117	-26

Leakage (Mt) 0.0 1.0 2.0 3.0 4.0 5.0

GHG (Mt CO_2-eq) −200 0 200 400 600

Supplementary Figure 16. Environmental leakage and GHG emissions of 14 plastics under Collection in 2060. S1, S2, S3 and S4 represent production, T&M, use and disposal, respectively. Total refers to the total amount of environmental leakage. Aq. refers to aquatic environment leakage. Ter. refers to terrestrial environmental leakage. Net. refers to net GHG emissions. Emiss. refers to total GHG emissions. Offset refers to GHG offsets from recycling substitution and incineration for electricity generation.

Mechanical recycling

The "Research Report on the Current Status of Recycling and Utilization of Low-Value Recyclables in China"[49] indicates that in 2021, the average recycling and utilization rate of major renewable resources in our country reached over 75%. However, the annual generation of various types of low-value recyclables was approximately 95.77 million tons, with a recycling volume of about 25.47 million tons, resulting in an overall recycling rate of about 26.6%. This suggests that the low recycling and utilization rate of waste resources is primarily due to the low recycling and utilization rate of low-value recyclables. Low-value recyclables mainly consist of discarded textiles, waste glass, agricultural mulch films, low-value plastic packaging, and beverage carton composite packaging. These products are widely used in daily life and correspond to a high proportion of the total waste generated. However, due to imperfect recycling systems and the inconvenience of recycling, their overall recycling and utilization rate is extremely low. We assume that by 2060, the mechanical recycling rate will reach 50%.

	S1	S2	S3	S4	Total	Aq.	Ter.	S1	S2	S3	S4	Net.	Emiss.	Offset
HDPE	0.18	0.30	0.34	0.61	1.44	0.38	1.06	36	13	0	1	50	64	-14
LDPE	0.22	0.37	0.04	1.09	1.72	0.09	1.63	44	26	0	5	74	98	-24
PP	0.36	0.52	0.05	1.36	2.29	0.11	2.18	72	28	0	9	110	136	-26
EPS	0.01	0.02	0.01	0.10	0.15	0.01	0.14	4	1	0	1	5	8	-2
GPPS	0.02	0.03	0.01	0.08	0.14	0.01	0.13	5	2	0	1	8	9	-1
PVC	0.22	0.30	0.01	0.92	1.44	0.04	1.40	54	11	0	9	74	88	-14
ABS	0.05	0.09	0.00	0.26	0.39	0.01	0.38	17	8	0	-0	25	31	-7
PET	0.76	1.05	0.05	1.85	3.72	0.16	3.56	332	82	0	-78	337	464	-127
PA	0.06	0.09	0.01	0.24	0.40	0.02	0.38	55	8	0	-15	47	66	-19
PC	0.01	0.03	0.00	0.11	0.15	0.00	0.15	6	2	0	0	8	11	-2
POM	0.00	0.01	0.00	0.03	0.04	0.00	0.04	1	1	0	0	2	2	-0
PBT	0.01	0.01	0.00	0.03	0.06	0.00	0.06	4	1	0	-0	5	6	-1
PPS	0.01	0.02	0.00	0.04	0.07	0.00	0.07	3	1	0	-0	4	5	-1
Others	0.29	0.44	0.06	1.23	2.01	0.11	1.90	60	21	0	-1	80	112	-32

Leakage (Mt): 0.0 1.0 2.0 3.0 4.0 5.0

GHG (Mt CO_2-eq): −200 0 200 400 600

Supplementary Figure 17. Environmental leakage and GHG emissions of 14 plastics under Mechanical recycling in 2060. S1, S2, S3 and S4 represent production, T&M, use and disposal, respectively. Total refers to the total amount of environmental leakage. Aq. refers to aquatic environment leakage. Ter. refers to terrestrial environmental leakage. Net. refers to net GHG emissions. Emiss. refers to total GHG emissions. Offset refers to GHG offsets from recycling substitution and incineration for electricity generation.

Chemical recycling

A primary advantage of chemical recycling is its capability to process low-value, mixed, and contaminated waste plastics, with the resulting products being equivalent in quality to petrochemical-based plastics and suitable for high-value applications such as food and pharmaceuticals. This complements physical recycling, which is more appropriate for high-value, single-category, and relatively clean waste plastics. We assume that by 2060, the rate of chemical recycling will reach 20%.

Supplementary Table 33. Carbon emission per unit of chemical recycling (t CO_2 -eq per t)[50].

Chemical recycling of carbon emissions	Chemical recycling carbon substitution
0.78	0.82

	S1	S2	S3	S4	Total	Aq.	Ter.	S1	S2	S3	S4	Net.	Emiss.	Offset
HDPE	0.18	0.30	0.34	0.61	1.43	0.37	1.06	36	13	0	6	54	67	-13
LDPE	0.22	0.37	0.04	1.06	1.69	0.09	1.60	44	26	1	11	81	104	-22
PP	0.36	0.52	0.05	1.25	2.18	0.10	2.08	72	28	2	14	117	142	-25
EPS	0.01	0.02	0.01	0.09	0.13	0.01	0.12	4	1	3	1	9	11	-2
GPPS	0.02	0.03	0.01	0.07	0.12	0.01	0.11	5	2	4	1	12	13	-1
PVC	0.22	0.30	0.01	0.75	1.28	0.04	1.24	54	11	5	9	79	93	-13
ABS	0.05	0.09	0.00	0.23	0.37	0.01	0.36	17	8	6	2	32	38	-5
PET	0.76	1.05	0.05	2.05	3.92	0.17	3.76	332	82	7	-19	402	487	-85
PA	0.06	0.09	0.01	0.22	0.38	0.02	0.36	55	8	8	-6	64	75	-11
PC	0.01	0.03	0.00	0.09	0.13	0.00	0.13	6	2	9	1	18	20	-2
POM	0.00	0.01	0.00	0.03	0.04	0.00	0.03	1	1	10	0	12	12	-0
PBT	0.01	0.01	0.00	0.03	0.06	0.00	0.05	4	1	11	0	16	17	-1
PPS	0.01	0.02	0.00	0.04	0.07	0.00	0.07	3	1	12	0	16	17	-1
Others	0.29	0.44	0.06	1.07	1.86	0.11	1.75	60	21	13	7	101	126	-25

Leakage (Mt): 0.0 1.0 2.0 3.0 4.0 5.0

GHG (Mt CO_2-eq): −200 0 200 400 600

Supplementary Figure 18. Environmental leakage and GHG emissions of 14 plastics under Chemical recycling in 2060. S1, S2, S3 and S4 represent production, T&M, use and disposal, respectively. Total refers to the total amount of environmental leakage. Aq. refers to aquatic environment leakage. Ter. refers to terrestrial environmental leakage. Net. refers to net GHG emissions. Emiss. refers to total GHG emissions. Offset refers to GHG offsets from recycling substitution and incineration for electricity generation.

Incineration

Since 2000, the Chinese government has sequentially issued a series of policies that encourage the development of the waste incineration industry. These policies have mobilized the enthusiasm of local governments and enterprises, effectively promoting the development of the "entire industrial chain" of waste incineration. For instance, from 2001 to 2010, China's waste-to-energy incineration industry underwent a phase of trial and improvement, benefiting from policies such as grid connection, preferential electricity prices, and a value-added tax refund scheme. From 2011 to the present, the industry has entered a stage of rapid development, with the government promoting incineration power generation technology, particularly in cities with scarce land resources and high population densities. Currently, the total treatment capacity of China's waste incineration power plants reaches approximately 1.035 million tons per day. The construction and operation of these facilities are aimed at addressing the growing issue of urban household waste on one hand, and at improving the efficiency and environmental standards of waste treatment on the other. The significant impact of the proportion of plastic waste on carbon emissions is due to the fact that plastic waste, as a product of fossil petroleum, has a total carbon content of up to 85%, and the carbon is entirely of fossil origin. The CO_2 emitted during combustion is classified as direct carbon emissions. We assume that by 2060, the proportion of incineration will reach 60%.

	S1	S2	S3	S4	Total	Aq.	Ter.	S1	S2	S3	S4	Net.	Emiss.	Offset
**HDPE**	0.18	0.30	0.34	0.61	1.43	0.37	1.06	36	13	0	6	54	67	−13
**LDPE**	0.22	0.37	0.04	1.06	1.69	0.09	1.60	44	26	0	11	81	104	−22
**PP**	0.36	0.52	0.05	1.25	2.18	0.10	2.08	72	28	0	14	117	142	−25
**EPS**	0.01	0.02	0.01	0.09	0.13	0.01	0.12	4	1	0	1	9	11	−2
**GPPS**	0.02	0.03	0.01	0.07	0.12	0.01	0.11	5	2	0	1	12	13	−1
**PVC**	0.22	0.30	0.01	0.75	1.28	0.04	1.24	54	11	0	9	79	93	−13
**ABS**	0.05	0.09	1.76E−4	0.23	0.37	0.01	0.36	17	8	0	2	32	38	−5
**PET**	0.76	1.05	0.05	2.05	3.92	0.17	3.76	332	82	0	−19	402	487	−85
**PA**	0.06	0.09	0.01	0.22	0.38	0.02	0.36	55	8	0	−6	64	75	−11
**PC**	0.01	0.03	7.47E−4	0.09	0.13	0.00	0.13	6	2	0	1	18	20	−2
**POM**	0.00	0.01	0.00	0.03	0.04	0.00	0.03	1	1	0	0	12	12	−0
**PBT**	0.01	0.01	4.46E−4	0.03	0.06	0.00	0.05	4	1	0	0	16	17	−1
**PPS**	0.01	0.02	0.00	0.04	0.07	0.00	0.07	3	1	0	0	16	17	−1
**Others**	0.29	0.44	0.06	1.07	1.86	0.11	1.75	60	21	0	7	101	126	−25

Leakage (Mt) 0.0 1.0 2.0 3.0 4.0 5.0

GHG (Mt CO_2-eq) −200 0 200 400 600

Supplementary Figure 19. Environmental leakage and GHG emissions of 14 plastics under Incineration in 2060. S1, S2, S3 and S4 represent production, T&M, use and disposal, respectively. Total refers to the total amount of environmental leakage. Aq. refers to aquatic environment leakage. Ter. refers to terrestrial environmental leakage. Net. refers to net GHG emissions. Emiss. refers to total GHG emissions. Offset refers to GHG offsets from recycling substitution and incineration for electricity generation.

Landfilling

We assume that by 2060, the proportion of landfilling will reach 30%.

	S1	S2	S3	S4	Total	Aq.	Ter.	S1	S2	S3	S4	Net.	Emiss.	Offset
**HDPE**	0.18	0.30	0.34	0.42	1.25	0.37	0.88	36	13	0	7	55	67	-11
**LDPE**	0.22	0.37	0.04	0.73	1.36	0.08	1.28	44	26	0	13	82	103	-20
**PP**	0.36	0.52	0.05	0.86	1.79	0.09	1.70	72	28	0	17	118	140	-22
**EPS**	0.01	0.02	0.01	0.06	0.11	0.01	0.10	4	1	0	1	6	8	-2
**GPPS**	0.02	0.03	0.01	0.05	0.10	0.01	0.09	5	2	0	1	8	9	-1
**PVC**	0.22	0.30	0.01	0.51	1.04	0.03	1.01	54	11	0	12	77	88	-12
**ABS**	0.05	0.09	0.00	0.16	0.30	0.01	0.29	17	8	0	2	27	32	-5
**PET**	0.76	1.05	0.05	1.42	3.29	0.14	3.14	332	82	0	-24	391	478	-87
**PA**	0.06	0.09	0.01	0.15	0.31	0.02	0.29	55	8	0	-7	55	67	-12
**PC**	0.01	0.03	0.00	0.06	0.11	0.00	0.10	6	2	0	1	9	11	-2
**POM**	0.00	0.01	0.00	0.02	0.03	0.00	0.03	1	1	0	0	2	2	-0
**PBT**	0.01	0.01	0.00	0.02	0.05	0.00	0.05	4	1	0	0	5	6	-1
**PPS**	0.01	0.02	0.00	0.03	0.06	0.00	0.06	3	1	0	0	4	5	-1
**Others**	0.29	0.44	0.06	0.74	1.52	0.09	1.43	60	21	0	9	90	114	-24

Leakage (Mt) — 0.0, 1.0, 2.0, 3.0, 4.0, 5.0

GHG (Mt CO_2-eq) — −200, 0, 200, 400, 600

Supplementary Figure 20. Environmental leakage and GHG emissions of 14 plastics under Landfilling in 2060. S1, S2, S3 and S4 represent production, T&M, use and disposal, respectively. Total refers to the total amount of environmental leakage. Aq. refers to aquatic environment leakage. Ter. refers to terrestrial environmental leakage. Net. refers to net GHG emissions. Emiss. refers to total GHG emissions. Offset refers to GHG offsets from recycling substitution and incineration for electricity generation.

Reduction and substitution

This integrated scenario combines reform of the energy structure, design optimization for reduction, reduction in demand, and reduction through substitution. This scenario focuses on the synergistic emission reduction effects of source reduction and energy transformation at the front end of the supply chain. Under baseline assumptions maintaining constant scenario weightings during individual variable modifications, key targets include: (1) Reform of the energy structure. (2) 90% source reduction in packaging through design optimization alongside prohibitions on plastic-microbead household chemicals and sub-0.01mm PE mulch films.

	S1	S2	S3	S4	Total	Aq.	Ter.	S1	S2	S3	S4	Net.	Emiss.	Offset
**HDPE**	0.08	0.04	0.00	0.47	0.59	0.02	0.57	6	6	0	6	18	21	-3
**LDPE**	0.10	0.05	0.00	0.69	0.84	0.03	0.81	7	12	0	10	29	34	-5
**PP**	0.04	0.06	0.01	0.90	1.01	0.03	0.98	14	13	0	14	42	46	-4
**EPS**	0.00	0.00	0.00	0.04	0.04	0.00	0.04	1	1	0	1	3	3	-0
**GPPS**	0.00	0.00	0.00	0.07	0.07	0.00	0.07	1	1	0	1	3	3	-0
**PVC**	0.04	0.06	0.01	0.85	0.96	0.03	0.93	18	4	0	13	35	38	-3
**ABS**	0.01	0.02	0.00	0.29	0.32	0.01	0.31	6	2	0	4	11	13	-2
**PET**	0.14	0.19	0.05	2.19	2.57	0.14	2.44	92	26	0	22	140	164	-24
**PA**	0.01	0.02	0.00	0.27	0.30	0.01	0.29	19	2	0	-1	21	25	-4
**PC**	0.00	0.00	0.00	0.09	0.09	0.00	0.09	1	1	0	1	3	3	-0
**POM**	0.00	0.00	0.00	0.03	0.03	0.00	0.03	0	0	0	0	1	1	-0
**PBT**	0.00	0.00	0.00	0.04	0.04	0.00	0.04	1	0	0	0	2	2	-0
**PPS**	0.00	0.00	0.00	0.05	0.06	0.00	0.06	1	0	0	0	2	2	-0
**Others**	0.05	0.08	0.01	1.06	1.20	0.04	1.15	18	8	0	13	39	46	-7

Leakage (Mt): 0.0 1.0 2.0 3.0 4.0 5.0
GHG (Mt CO_2-eq): −200 0 200 400 600

Supplementary Figure 21. Environmental leakage and GHG emissions of 14 plastics under Reduction and substitution in 2060. S1, S2, S3 and S4 represent production, T&M, use and disposal, respectively. Total refers to the total amount of environmental leakage. Aq. refers to aquatic environment leakage. Ter. refers to terrestrial environmental leakage. Net. refers to net GHG emissions. Emiss. refers to total GHG emissions. Offset refers to GHG offsets from recycling substitution and incineration for electricity generation.

Recycling

This scenario integrates mechanical recycling and chemical recycling, with a particular emphasis on the synergistic emission reduction benefits that can be achieved by increased recycling rate strategies alone. Under baseline assumptions maintaining constant scenario weightings during individual variable modifications, key targets include: (1) mechanical recycling achieving 35%; and (2) chemical recycling reaching 25%.

	S1	S2	S3	S4	Total	Aq.	Ter.	S1	S2	S3	S4	Net.	Emiss.	Offset
**HDPE**	0.18	0.30	0.34	0.25	1.08	0.36	0.71	36	13	0	6	54	71	-16
**LDPE**	0.22	0.37	0.04	0.43	1.06	0.07	0.99	44	26	0	9	79	106	-28
**PP**	0.36	0.52	0.05	0.50	1.43	0.08	1.35	72	28	0	9	110	138	-28
**EPS**	0.01	0.02	0.01	0.04	0.08	0.01	0.07	4	1	0	0	5	7	-2
**GPPS**	0.02	0.03	0.01	0.03	0.08	0.01	0.07	5	2	0	0	7	8	-1
**PVC**	0.22	0.30	0.01	0.30	0.82	0.02	0.80	54	11	0	2	67	78	-12
**ABS**	0.05	0.09	0.00	0.09	0.23	0.01	0.22	17	8	0	-0	25	31	-6
**PET**	0.76	1.05	0.05	0.84	2.70	0.13	2.58	332	82	0	-33	381	508	-127
**PA**	0.06	0.09	0.01	0.09	0.25	0.02	0.23	55	8	0	-11	51	67	-16
**PC**	0.01	0.03	0.00	0.04	0.08	0.00	0.08	6	2	0	-0	8	9	-2
**POM**	0.00	0.01	0.00	0.01	0.02	0.00	0.02	1	1	0	0	1	2	-0
**PBT**	0.01	0.01	0.00	0.01	0.04	0.00	0.04	4	1	0	-0	5	6	-1
**PPS**	0.01	0.02	0.00	0.02	0.05	0.00	0.04	3	1	0	0	4	5	-1
**Others**	0.29	0.44	0.06	0.43	1.21	0.08	1.13	60	21	0	-3	78	107	-29

Leakage (Mt): 0.0 1.0 2.0 3.0 4.0 5.0

GHG (Mt CO_2-eq): −200 0 200 400 600

Supplementary Figure 22. Environmental leakage and GHG emissions of 14 plastics under Recycling in 2060. S1, S2, S3 and S4 represent production, T&M, use and disposal, respectively. Total refers to the total amount of environmental leakage. Aq. refers to aquatic environment leakage. Ter. refers to terrestrial environmental leakage. Net. refers to net GHG emissions. Emiss. refers to total GHG emissions. Offset refers to GHG offsets from recycling substitution and incineration for electricity generation.

Collect and dispose

The program focuses on waste collection and disposal management and assesses their overall contribution to synergistic abatement potential while prioritizing leakage control. Under baseline assumptions maintaining constant scenario weightings during individual variable modifications, key targets include: (1) mismanaged waste incidence reduced to 1%; (2) incineration achieving 50%; and (3) landfilling limited to 19%.

	Leakage (Mt)							GHG (Mt CO_2-eq)						
	S1	S2	S3	S4	Total	Aq.	Ter.	S1	S2	S3	S4	Net.	Emiss.	Offset
HDPE	0.18	0.30	0.32	0.25	1.05	0.34	0.71	36	13	0	9	57	71	-13
LDPE	0.22	0.37	0.01	0.42	1.02	0.04	0.98	44	26	0	17	87	111	-24
PP	0.36	0.52	0.02	0.49	1.39	0.05	1.34	72	28	0	22	123	150	-27
EPS	0.01	0.02	0.00	0.03	0.07	0.00	0.07	4	1	0	1	6	8	-2
GPPS	0.02	0.03	0.01	0.03	0.08	0.01	0.07	5	2	0	1	8	10	-1
PVC	0.22	0.30	0.01	0.28	0.80	0.02	0.78	54	11	0	15	80	95	-15
ABS	0.05	0.09	0.00	0.09	0.23	0.01	0.22	17	8	0	3	28	34	-6
PET	0.76	1.05	0.05	0.85	2.72	0.12	2.59	332	82	0	-19	396	492	-97
PA	0.06	0.09	0.01	0.08	0.25	0.02	0.23	55	8	0	-7	55	68	-13
PC	0.01	0.03	0.00	0.03	0.08	0.00	0.07	6	2	0	2	10	11	-2
POM	0.00	0.01	0.00	0.01	0.02	0.00	0.02	1	1	0	0	2	2	-0
PBT	0.01	0.01	0.00	0.01	0.04	0.00	0.04	4	1	0	0	5	6	-1
PPS	0.01	0.02	0.00	0.02	0.05	0.00	0.04	3	1	0	0	4	5	-1
Others	0.29	0.44	0.01	0.41	1.15	0.03	1.11	60	21	0	13	94	123	-28

Leakage (Mt): 0.0 1.0 2.0 3.0 4.0 5.0
GHG (Mt CO_2-eq): −200 0 200 400 600

Supplementary Figure 23. Environmental leakage and GHG emissions of 14 plastics under Collect and dispose in 2060. S1, S2, S3 and S4 represent production, T&M, use and disposal, respectively. Total refers to the total amount of environmental leakage. Aq. refers to aquatic environment leakage. Ter. refers to terrestrial environmental leakage. Net. refers to net GHG emissions. Emiss. refers to total GHG emissions. Offset refers to GHG offsets from recycling substitution and incineration for electricity generation.

System change

This scenario integrates all interventions across sectors to achieve structural decarbonization and elimination of plastic leakage. Under baseline assumptions maintaining constant scenario weightings during individual variable modifications, key targets comprise: (1) Energy structure reform; (2) 90% packaging source reduction with prohibitions on plastic-microbead household chemicals and sub-0.01mm PE mulch films; (3) ≤1% mismanaged waste incidence; (4) Mechanical recycling rate at 30%; (5) Chemical recycling rate at 18%; (6) Incineration rate at 30%; and (7) Landfilling capped at 21%.

Supplementary Table 34. Microplastic leakage amounts by pathway in 2021 (Tons)

			HDPE	LDPE	PP	EPS	GPPS	PVC	ABS	PET	PA	PC	POM	PBT	PPS	Others
Aquatic	Production		5112	6248	14298	740	904	10600	2268	28500	2226	650	179	427	339	10729
Aquatic	Manufacturing		506	618	1415	73	89	1049	224	2820	220	64	18	42	34	1062
Aquatic	Personal care products		26766	892	892	0	892	0	0	0	892	0	0	0	0	0
Aquatic	Textile washing		0	48	0	0	0	0	0	40612	70	0	0	348	0	244
Aquatic	Recycle	Packaging	59	76	124	8	2	18	0	452	0	3	0	0	0	15
Aquatic	Recycle	Agriculture	23	62	0	0	0	0	0	0	0	0	0	0	0	25
Aquatic	Recycle	B&C	0	0	0	0	0	0	0	0	0	0	0	0	0	0
Aquatic	Recycle	Medical	8	0	0	0	0	3	0	0	0	0	0	0	0	9
Aquatic	Recycle	Transportation	3	0	13	0	0	11	2	0	4	0	0	1	0	9
Aquatic	Recycle	E&E	3	2	11	0	4	7	23	4	11	1	1	1	1	27
Aquatic	Recycle	Consumer	6	2	4	0	1	14	6	0	0	2	1	0	0	1
Aquatic	Recycle	Electric power communication	0	0	0	0	0	3	0	0	0	0	0	0	0	1
Aquatic	Recycle	Textile	0	0	12	0	0	0	0	362	0	0	0	2	0	2
Aquatic	Recycle	Industry Machinery	0	0	0	0	0	0	0	0	1	0	0	0	0	1

Terrestrial	Transport		2818	3444	5956	462	564	3825	1215	10433	895	484	138	141	136	4632
Terrestrial	Indoor dust		757	733	6275	531	145	8230	136	0	9	579	0	0	0	3852
Terrestrial	Public dust		668	647	5536	469	128	7261	120	0	8	511	0	0	0	3399
Terrestrial	Landfill	Packaging	276	573	581	66	20	124	0	228	3	31	0	0	0	186
Terrestrial	Landfill	Agriculture	20	188	0	0	0	0	0	0	0	0	0	0	0	118
Terrestrial	Landfill	B&C	8	2	16	3	0	8	0	0	0	0	0	0	0	5
Terrestrial	Landfill	Medical	62	2	0	0	1	24	0	0	0	1	0	0	0	74
Terrestrial	Landfill	Transportation	16	1	63	1	0	53	8	0	19	1	1	6	1	69
Terrestrial	Landfill	E&E	13	9	55	1	18	36	115	21	56	4	6	3	4	133
Terrestrial	Landfill	Consumer	107	29	72	1	15	251	110	0	1	31	12	4	0	20
Terrestrial	Landfill	Electric power communication	8	9	0	0	0	59	0	0	0	0	0	1	0	10
Terrestrial	Landfill	Textile	0	2	73	0	0	0	0	2184	2	0	0	14	0	10

Terrestrial	Landfill	Industry Machinery	6	0	0	0	0	0	0	0	20	0	0	0	0	22

Supplementary Table 35. Macroplastic leakage amounts by pathway in 2021 (Ten thousand tons)

			HDPE	LDPE	PP	EPS	GPPS	PVC	ABS	PET	PA	PC	POM	PBT	PPS	other
Aquatic	Aquaculture		2.00	2.00	2.00	0.60	0.00	0.00	0.00	0.00	0.00	0.00	0.00	0.00	0.00	3.00
Aquatic	Fisheries		0.60	1.00	1.00	0.09	0.00	0.00	0.00	0.00	0.00	0.00	0.00	0.00	0.00	2.00
Aquatic	watershed	Packaging	0.46	0.91	0.96	0.10	0.03	0.20	0.00	0.68	0.00	0.05	0.00	0.00	0.00	0.29
Aquatic	watershed	Agriculture	0.05	0.33	0.00	0.00	0.00	0.00	0.00	0.00	0.00	0.00	0.00	0.00	0.00	0.19
Aquatic	watershed	B&C	0.01	0.00	0.02	0.01	0.00	0.01	0.00	0.00	0.00	0.00	0.00	0.00	0.00	0.01
Aquatic	watershed	Medical	0.10	0.00	0.00	0.00	0.00	0.04	0.00	0.00	0.00	0.00	0.00	0.00	0.00	0.12
Aquatic	watershed	Transportation	0.03	0.00	0.10	0.00	0.00	0.09	0.01	0.00	0.03	0.00	0.00	0.01	0.00	0.11
Aquatic	watershed	E&E	0.02	0.01	0.09	0.00	0.03	0.06	0.19	0.03	0.09	0.01	0.01	0.00	0.01	0.22
Aquatic	watershed	Consumer	0.16	0.04	0.11	0.00	0.02	0.38	0.17	0.00	0.00	0.05	0.02	0.01	0.00	0.03
Aquatic	watershed	Electric power communication	0.01	0.01	0.00	0.00	0.00	0.09	0.00	0.00	0.00	0.00	0.00	0.00	0.00	0.02

Aquatic	watershed	Textile	0.00	0.00	0.12	0.00	0.00	0.00	0.00	3.52	0.00	0.00	0.00	0.02	0.00	0.02
Aquatic	watershed	Industry Machinery	0.01	0.00	0.00	0.00	0.00	0.00	0.00	0.00	0.03	0.00	0.00	0.00	0.00	0.03
Aquatic	coastal area	Packaging	0.05	0.10	0.11	0.01	0.00	0.02	0.00	0.08	0.00	0.01	0.00	0.00	0.00	0.03
Aquatic	coastal area	Agriculture	0.01	0.04	0.00	0.00	0.00	0.00	0.00	0.00	0.00	0.00	0.00	0.00	0.00	0.02
Aquatic	coastal area	B&C	0.00	0.00	0.00	0.00	0.00	0.00	0.00	0.00	0.00	0.00	0.00	0.00	0.00	0.00
Aquatic	coastal area	Medical	0.01	0.00	0.00	0.00	0.00	0.00	0.00	0.00	0.00	0.00	0.00	0.00	0.00	0.01
Aquatic	coastal area	Transportation	0.00	0.00	0.01	0.00	0.00	0.01	0.00	0.00	0.00	0.00	0.00	0.00	0.00	0.01
Aquatic	coastal area	E&E	0.00	0.00	0.01	0.00	0.00	0.01	0.02	0.00	0.01	0.00	0.00	0.00	0.00	0.02
Aquatic	coastal area	Consumer	0.02	0.01	0.01	0.00	0.00	0.04	0.02	0.00	0.00	0.01	0.00	0.00	0.00	0.00
Aquatic	coastal area	Electric power communication	0.00	0.00	0.00	0.00	0.00	0.01	0.00	0.00	0.00	0.00	0.00	0.00	0.00	0.00
Aquatic	coastal area	Textile	0.00	0.00	0.01	0.00	0.00	0.00	0.00	0.40	0.00	0.00	0.00	0.00	0.00	0.00

Aquatic	coastal area	Industry Machinery	0.00	0.00	0.00	0.00	0.00	0.00	0.00	0.00	0.00	0.00	0.00	0.00	0.00	0.00
Terrestrial	Production		10.22	12.50	28.60	1.48	1.81	21.20	4.54	57.00	4.45	1.30	0.36	0.85	0.68	21.46
Terrestrial	Manufacturing		7.59	9.27	21.22	1.10	1.34	15.73	3.37	42.30	3.30	0.96	0.27	0.63	0.50	15.93
Terrestrial	Transport		10.57	12.92	22.34	1.73	2.12	14.34	4.56	39.12	3.36	1.81	0.52	0.53	0.51	17.37
Terrestrial	Recycle	Packaging	0.08	0.10	0.17	0.01	0.00	0.02	0.00	0.60	0.00	0.00	0.00	0.00	0.00	0.02
Terrestrial	Recycle	Agriculture	0.03	0.08	0.00	0.00	0.00	0.00	0.00	0.00	0.00	0.00	0.00	0.00	0.00	0.03
Terrestrial	Recycle	B&C	0.00	0.00	0.00	0.00	0.00	0.00	0.00	0.00	0.00	0.00	0.00	0.00	0.00	0.00
Terrestrial	Recycle	Medical	0.01	0.00	0.00	0.00	0.00	0.00	0.00	0.00	0.00	0.00	0.00	0.00	0.00	0.01
Terrestrial	Recycle	Transportation	0.00	0.00	0.02	0.00	0.00	0.02	0.00	0.00	0.01	0.00	0.00	0.00	0.00	0.01
Terrestrial	Recycle	E&E	0.00	0.00	0.01	0.00	0.00	0.01	0.03	0.01	0.02	0.00	0.00	0.00	0.00	0.04
Terrestrial	Recycle	Consumer	0.01	0.00	0.01	0.00	0.00	0.02	0.01	0.00	0.00	0.00	0.00	0.00	0.00	0.00
Terrestrial	Recycle	Electric power communication	0.00	0.00	0.00	0.00	0.00	0.00	0.00	0.00	0.00	0.00	0.00	0.00	0.00	0.00

Terrestrial	Recycle	Textile	0.00	0.00	0.02	0.00	0.00	0.00	0.00	0.48	0.00	0.00	0.00	0.00	0.00	0.00
Terrestrial	Recycle	Industry Machinery	0.00	0.00	0.00	0.00	0.00	0.00	0.00	0.00	0.00	0.00	0.00	0.00	0.00	0.00
Terrestrial	Landfill	Packaging	0.02	0.05	0.05	0.01	0.00	0.01	0.00	0.02	0.00	0.00	0.00	0.00	0.00	0.02
Terrestrial	Landfill	Agriculture	0.00	0.02	0.00	0.00	0.00	0.00	0.00	0.00	0.00	0.00	0.00	0.00	0.00	0.01
Terrestrial	Landfill	B&C	0.00	0.00	0.00	0.00	0.00	0.00	0.00	0.00	0.00	0.00	0.00	0.00	0.00	0.00
Terrestrial	Landfill	Medical	0.01	0.00	0.00	0.00	0.00	0.00	0.00	0.00	0.00	0.00	0.00	0.00	0.00	0.01
Terrestrial	Landfill	Transportation	0.00	0.00	0.01	0.00	0.00	0.00	0.00	0.00	0.00	0.00	0.00	0.00	0.00	0.01
Terrestrial	Landfill	E&E	0.00	0.00	0.00	0.00	0.00	0.00	0.01	0.00	0.00	0.00	0.00	0.00	0.00	0.01
Terrestrial	Landfill	Consumer	0.01	0.00	0.01	0.00	0.00	0.02	0.01	0.00	0.00	0.00	0.00	0.00	0.00	0.00
Terrestrial	Landfill	Electric power communication	0.00	0.00	0.00	0.00	0.00	0.00	0.00	0.00	0.00	0.00	0.00	0.00	0.00	0.00
Terrestrial	Landfill	Textile	0.00	0.00	0.01	0.00	0.00	0.00	0.00	0.18	0.00	0.00	0.00	0.00	0.00	0.00

Terrestrial	Landfill	Industry Machinery	0.00	0.00	0.00	0.00	0.00	0.00	0.00	0.00	0.00	0.00	0.00	0.00	0.00	0.00
Terrestrial	Mismanagement of land leakage	Packaging	14.17	28.29	29.79	3.23	0.97	6.15	0.00	21.07	0.15	1.51	0.00	0.00	0.00	8.95
Terrestrial	Mismanagement of land leakage	Agriculture	1.43	10.16	0.00	0.00	0.00	0.00	0.00	0.00	0.00	0.00	0.00	0.00	0.00	6.03
Terrestrial	Mismanagement of land leakage	B&C	0.37	0.08	0.77	0.16	0.02	0.39	0.01	0.00	0.00	0.01	0.00	0.00	0.00	0.25
Terrestrial	Mismanagement of land leakage	Medical	3.06	0.12	0.00	0.00	0.03	1.20	0.00	0.00	0.00	0.07	0.00	0.00	0.00	3.62
Terrestrial	Mismanagement of land leakage	Transportation	0.80	0.05	3.24	0.05	0.02	2.71	0.42	0.00	0.99	0.07	0.05	0.30	0.03	3.38
Terrestrial	Mismanagement of land leakage	E&E	0.65	0.45	2.82	0.04	0.91	1.81	5.86	1.06	2.85	0.19	0.29	0.14	0.20	6.77

Terrestrial	Mismanagement of land leakage	Consumer	5.11	1.38	3.41	0.07	0.72	11.94	5.26	0.00	0.06	1.48	0.58	0.21	0.01	0.97
Terrestrial	Mismanagement of land leakage	Electric power communication	0.37	0.43	0.00	0.00	0.00	2.79	0.00	0.00	0.00	0.00	0.00	0.06	0.00	0.48
Terrestrial	Mismanagement of land leakage	Textile	0.00	0.09	3.67	0.00	0.00	0.00	0.00	109.58	0.12	0.00	0.00	0.71	0.00	0.50
Terrestrial	Mismanagement of land leakage	Industry Machinery	0.26	0.00	0.00	0.00	0.00	0.00	0.00	0.00	0.95	0.01	0.00	0.00	0.02	1.04

Supplementary Table 36. Microplastic leakage amounts by pathway in 2060 (Tons)

		HDPE	LDPE	PP	EPS	GPPS	PVC	ABS	PET	PA	PC	POM	PBT	PPS	Others
Aquatic	Production	882.79	1078.96	2053.58	35.66	43.59	1978.65	536.43	6565.56	646.00	70.32	34.52	115.29	125.72	2390.95
Aquatic	Manufacturing	88.27	107.88	204.88	3.56	4.35	197.41	53.52	655.04	64.45	7.02	3.44	11.50	12.54	238.54
Aquatic	Textile washing	61.47	52105.00	0.00	0.00	0.00	0.00	0.00	0.00	90.17	0.00	0.00	446.13	0.00	312.53

Aquatic	Recycle	Packaging	18.64	26.37	44.91	3.02	0.65	5.94	0.00	100.17	0.11	0.95	0.00	0.00	0.00	7.91
Aquatic	Recycle	Agriculture	148.06	407.88	0.00	0.00	0.00	0.00	0.00	0.00	0.00	0.00	0.00	0.00	0.00	182.80
Aquatic	Recycle	B&C	2.56	2.27	19.22	1.71	0.44	25.22	0.42	0.00	0.00	1.75	0.00	0.00	0.00	11.98
Aquatic	Recycle	Medical	32.64	1.24	0.00	0.00	0.41	13.39	0.00	0.00	0.00	0.78	0.00	0.00	0.00	42.95
Aquatic	Recycle	Transportation	20.74	1.22	96.07	0.98	0.40	37.28	28.30	0.00	37.71	5.21	4.48	5.63	2.67	51.12
Aquatic	Recycle	E&E	9.43	6.68	41.32	0.73	4.72	19.21	64.82	13.40	51.04	2.15	0.62	1.51	17.34	63.53
Aquatic	Recycle	Consumer	13.59	3.58	16.85	0.25	6.73	31.77	15.36	0.00	0.26	5.39	1.86	0.57	0.37	4.12
Aquatic	Recycle	Electric power communication	4.04	4.23	0.00	0.00	0.00	15.30	0.00	0.00	0.00	0.00	0.00	0.46	0.00	4.57
Aquatic	Recycle	Textile	0.00	1.02	67.37	0.00	0.00	0.00	0.00	868.78	1.50	0.00	0.00	7.44	0.00	5.21
Aquatic	Recycle	Industry Machinery	7.50	0.00	0.00	0.00	0.00	0.00	0.00	0.00	13.67	0.55	0.89	0.00	1.34	2.60
Terrestrial	Transport		633.46	774.22	884.03	44.84	54.80	702.21	287.53	2354.49	259.73	77.45	26.46	38.13	50.56	1053.09
Terrestrial	Indoor dust		519.58	503.16	4305.22	####	99.78	5646.30	93.45	0.00	6.19	#####	0.00	0.00	0.00	2643.12

Terrestrial	Public dust		458.43	443.94	3798.52	####	88.04	4981.77	82.45	0.00	5.46	#####	0.00	0.00	0.00	2332.04
Terrestrial	Landfill	Packaging	9.96	18.99	19.14	2.34	0.73	3.87	0.00	0.68	0.13	1.07	0.00	0.00	0.00	9.83
Terrestrial	Landfill	Agriculture	4.48	103.19	0.00	0.00	0.00	0.00	0.00	0.00	0.00	0.00	0.00	0.00	0.00	77.91
Terrestrial	Landfill	B&C	9.94	8.84	74.78	6.64	1.69	98.14	1.63	0.00	0.11	6.81	0.00	0.00	0.00	46.60
Terrestrial	Landfill	Medical	25.22	0.96	0.00	0.00	0.31	10.35	0.00	0.00	0.00	0.60	0.00	0.00	0.00	33.18
Terrestrial	Landfill	Transportation	8.84	0.52	40.95	0.42	0.17	15.89	12.06	0.00	16.07	2.22	1.91	2.40	1.14	39.50
Terrestrial	Landfill	E&E	4.23	2.99	18.51	0.33	2.12	8.60	29.03	6.00	22.86	0.96	0.28	0.67	7.77	28.45
Terrestrial	Landfill	Consumer	24.63	6.48	30.52	0.45	12.20	57.56	27.83	21.22	0.47	9.77	3.37	1.04	0.67	7.46
Terrestrial	Landfill	Electric power communication	7.31	7.66	0.00	0.00	0.00	27.72	0.00	0.00	0.00	0.00	0.00	0.84	0.00	8.28
Terrestrial	Landfill	Textile	0.00	0.58	38.05	0.00	0.00	0.00	0.00	490.68	0.85	0.00	0.00	4.20	0.00	2.94
Terrestrial	Landfill	Industry Machinery	13.60	0.00	0.00	0.00	0.00	0.00	0.00	0.00	24.78	1.00	1.62	0.00	2.42	4.71

Supplementary Table 37. Macroplastic leakage amounts by pathway in 2060 (Ten thousand tons)

			HDPE	LDPE	PP	EPS	GPPS	PVC	ABS	PET	PA	PC	POM	PBT	PPS	other
Aquatic	watershed	Packaging	0.03	0.07	0.08	0.01	0.00	0.01	0.00	0.04	0.00	0.00	0.00	0.00	0.00	0.03
Aquatic	watershed	Agriculture	0.07	0.49	0.00	0.00	0.00	0.00	0.00	0.00	0.00	0.00	0.00	0.00	0.00	0.32
Aquatic	watershed	B&C	0.03	0.03	0.25	0.02	0.01	0.33	0.01	0.00	0.00	0.02	0.00	0.00	0.00	0.16
Aquatic	watershed	Medical	0.09	0.00	0.00	0.00	0.00	0.04	0.00	0.00	0.00	0.00	0.00	0.00	0.00	0.12
Aquatic	watershed	Transportation	0.04	0.00	0.17	0.00	0.00	0.07	0.05	0.00	0.07	0.01	0.01	0.01	0.00	0.15
Aquatic	watershed	E&E	0.02	0.01	0.08	0.00	0.01	0.04	0.12	0.02	0.09	0.00	0.00	0.00	0.03	0.12
Aquatic	watershed	Consumer	0.08	0.02	0.10	0.00	0.04	0.20	0.10	0.07	0.00	0.03	0.01	0.00	0.00	0.03
Aquatic	watershed	Electric power communication	0.03	0.03	0.00	0.00	0.00	0.10	0.00	0.00	0.00	0.00	0.00	0.00	0.00	0.03
Aquatic	watershed	Textile	0.00	0.00	0.15	0.00	0.00	0.00	0.00	1.91	0.00	0.00	0.00	0.02	0.00	0.01
Aquatic	watershed	Industry Machinery	0.05	0.00	0.00	0.00	0.00	0.00	0.00	0.00	0.09	0.00	0.01	0.00	0.01	0.02
Aquatic	coastal area	Packaging	0.00	0.01	0.01	0.00	0.00	0.00	0.00	0.00	0.00	0.00	0.00	0.00	0.00	0.00

Aquatic	coastal area	Agriculture	0.01	0.07	0.00	0.00	0.00	0.00	0.00	0.00	0.00	0.00	0.00	0.00	0.00	0.04
Aquatic	coastal area	B&C	0.00	0.00	0.03	0.00	0.00	0.04	0.00	0.00	0.00	0.00	0.00	0.00	0.00	0.02
Aquatic	coastal area	Medical	0.01	0.00	0.00	0.00	0.00	0.01	0.00	0.00	0.00	0.00	0.00	0.00	0.00	0.02
Aquatic	coastal area	Transportation	0.00	0.00	0.02	0.00	0.00	0.01	0.01	0.00	0.01	0.00	0.00	0.00	0.00	0.02
Aquatic	coastal area	E&E	0.00	0.00	0.01	0.00	0.00	0.00	0.02	0.00	0.01	0.00	0.00	0.00	0.00	0.02
Aquatic	coastal area	Consumer	0.01	0.00	0.01	0.00	0.01	0.03	0.01	0.01	0.00	0.00	0.00	0.00	0.00	0.00
Aquatic	coastal area	Electric power communication	0.00	0.00	0.00	0.00	0.00	0.01	0.00	0.00	0.00	0.00	0.00	0.00	0.00	0.00
Aquatic	coastal area	Textile	0.00	0.00	0.02	0.00	0.00	0.00	0.00	0.26	0.00	0.00	0.00	0.00	0.00	0.00
Aquatic	coastal area	Industry Machinery	0.01	0.00	0.00	0.00	0.00	0.00	0.00	0.00	0.01	0.00	0.00	0.00	0.00	0.00
Terrestrial	Production		7.84	9.59	4.11	0.07	0.09	3.96	1.07	13.13	1.29	0.14	0.07	0.23	0.25	4.78
Terrestrial	Manufacturing		1.32	1.62	3.07	0.05	0.07	2.96	0.80	9.83	0.97	0.11	0.05	0.17	0.19	3.58
Terrestrial	Transport		2.38	2.90	3.32	0.17	0.21	2.63	1.08	8.83	0.97	0.29	0.10	0.14	0.19	3.95

Terrestrial	Recycle	Packaging	0.02	0.04	0.06	0.00	0.00	0.01	0.00	0.13	0.00	0.00	0.00	0.00	0.00	0.01
Terrestrial	Recycle	Agriculture	0.20	0.54	0.00	0.00	0.00	0.00	0.00	0.00	0.00	0.00	0.00	0.00	0.00	0.24
Terrestrial	Recycle	B&C	0.00	0.00	0.03	0.00	0.00	0.03	0.00	0.00	0.00	0.00	0.00	0.00	0.00	0.02
Terrestrial	Recycle	Medical	0.04	0.00	0.00	0.00	0.00	0.02	0.00	0.00	0.00	0.00	0.00	0.00	0.00	0.06
Terrestrial	Recycle	Transportation	0.03	0.00	0.13	0.00	0.00	0.05	0.04	0.00	0.05	0.01	0.01	0.01	0.00	0.07
Terrestrial	Recycle	E&E	0.01	0.01	0.06	0.00	0.01	0.03	0.09	0.02	0.07	0.00	0.00	0.00	0.02	0.08
Terrestrial	Recycle	Consumer	0.02	0.00	0.02	0.00	0.01	0.04	0.02	0.00	0.00	0.01	0.00	0.00	0.00	0.01
Terrestrial	Recycle	Electric power communication	0.01	0.01	0.00	0.00	0.00	0.02	0.00	0.00	0.00	0.00	0.00	0.00	0.00	0.01
Terrestrial	Recycle	Textile	0.00	0.00	0.09	0.00	0.00	0.00	0.00	1.16	0.00	0.00	0.00	0.01	0.00	0.01
Terrestrial	Recycle	Industry Machinery	0.01	0.00	0.00	0.00	0.00	0.00	0.00	0.00	0.02	0.00	0.00	0.00	0.00	0.00
Terrestrial	Landfill	Packaging	0.00	0.00	0.00	0.00	0.00	0.00	0.00	0.00	0.00	0.00	0.00	0.00	0.00	0.00
Terrestrial	Landfill	Agriculture	0.00	0.01	0.00	0.00	0.00	0.00	0.00	0.00	0.00	0.00	0.00	0.00	0.00	0.01

Terrestrial	Landfill	B&C	0.00	0.00	0.01	0.00	0.00	0.01	0.00	0.00	0.00	0.00	0.00	0.00	0.00	0.00
Terrestrial	Landfill	Medical	0.00	0.00	0.00	0.00	0.00	0.00	0.00	0.00	0.00	0.00	0.00	0.00	0.00	0.00
Terrestrial	Landfill	Transportation	0.00	0.00	0.00	0.00	0.00	0.00	0.00	0.00	0.00	0.00	0.00	0.00	0.00	0.00
Terrestrial	Landfill	E&E	0.00	0.00	0.00	0.00	0.00	0.00	0.00	0.00	0.00	0.00	0.00	0.00	0.00	0.00
Terrestrial	Landfill	Consumer	0.00	0.00	0.00	0.00	0.00	0.00	0.00	0.00	0.00	0.00	0.00	0.00	0.00	0.00
Terrestrial	Landfill	Electric power communication	0.00	0.00	0.00	0.00	0.00	0.00	0.00	0.00	0.00	0.00	0.00	0.00	0.00	0.00
Terrestrial	Landfill	Textile	0.00	0.00	0.00	0.00	0.00	0.00	0.00	0.04	0.00	0.00	0.00	0.00	0.00	0.00
Terrestrial	Landfill	Industry Machinery	0.00	0.00	0.00	0.00	0.00	0.00	0.00	0.00	0.00	0.00	0.00	0.00	0.00	0.00
Terrestrial	Mismanagement of land leakage	Packaging	1.08	2.22	2.45	0.27	0.08	0.46	0.00	1.12	0.01	0.12	0.00	0.00	0.00	1.08
Terrestrial	Mismanagement of land leakage	Agriculture	2.20	15.19	0.00	0.00	0.00	0.00	0.00	0.00	0.00	0.00	0.00	0.00	0.00	9.99

Terrestrial	Mismanagement of land leakage	B&C	1.03	0.92	7.74	0.69	0.18	10.16	0.17	0.00	0.01	0.70	0.00	0.00	0.00	4.83
Terrestrial	Mismanagement of land leakage	Medical	2.92	0.11	0.00	0.00	0.04	1.20	0.00	0.00	0.00	0.07	0.00	0.00	0.00	3.84
Terrestrial	Mismanagement of land leakage	Transportation	1.13	0.07	5.25	0.05	0.02	2.04	1.55	0.00	2.06	0.28	0.24	0.31	0.15	4.57
Terrestrial	Mismanagement of land leakage	E&E	0.54	0.38	2.35	0.04	0.27	1.09	3.68	0.76	2.90	0.12	0.04	0.09	0.99	3.61
Terrestrial	Mismanagement of land leakage	Consumer	2.64	0.69	3.27	0.05	1.31	6.16	2.98	2.13	0.05	1.05	0.36	0.11	0.07	0.80
Terrestrial	Mismanagement of land leakage	Electric power communication	0.78	0.82	0.00	0.00	0.00	2.97	0.00	0.00	0.00	0.00	0.00	0.09	0.00	0.89

Terrestrial	Mismanagement of land leakage	Textile	0.00	0.07	4.62	0.00	0.00	0.00	0.00	59.59	0.10	0.00	0.00	0.51	0.00	0.36
Terrestrial	Mismanagement of land leakage	Industry Machinery	1.46	0.00	0.00	0.00	0.00	0.00	0.00	0.00	2.65	0.11	0.17	0.00	0.26	0.50

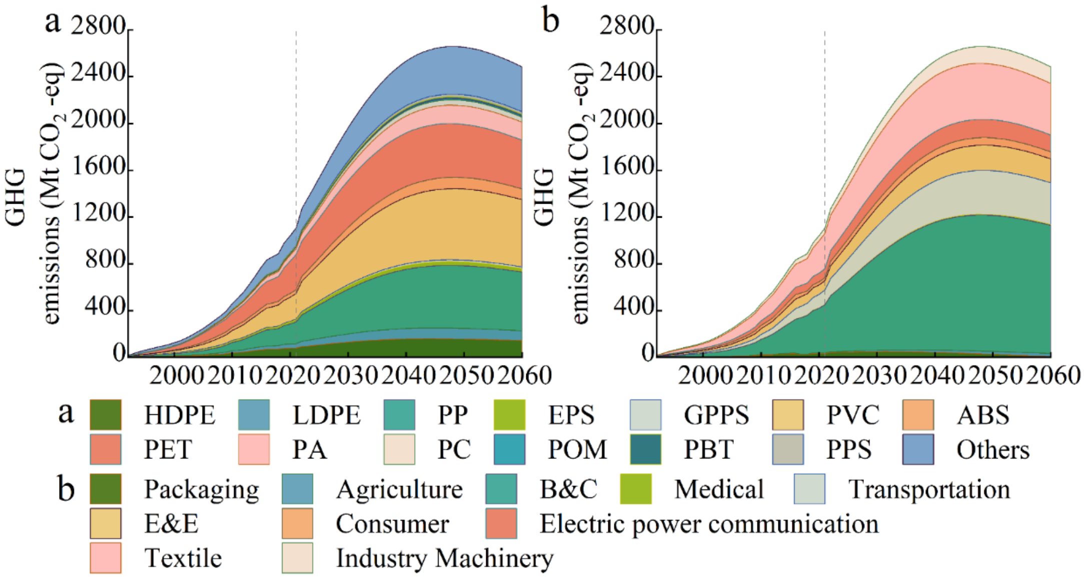

Supplementary Figure 24. Use carbon inventory generated by plastic types from 1992 to 2060 (a), and use carbon inventory generated by consumption sectors (b).

Supplementary Method 7. Cost and benefits

Expenditures and revenues associated with plastics pollution abatement are the main influencing factors in predicting the net economic cost of plastics synergistic abatement in China. In order to project the costs of different measures from 2022 to 2060, this study collected data on unit costs and unit revenues for plastic waste management activities[51]. Costs were calculated after mass flow calculations in the model to allow for rough order of magnitude comparisons of costs across scenarios. We modeled the waste management costs associated with each scenario through two primary cost considerations: projected annual capital expenditures (capex) and operating expenditures (opex)[3]. Capex is associated with major purchases or upgrades, such as industrial plant, equipment, vehicles, or land. Opex is the ongoing cost of producing and converting new materials, running recycling plants, salaries and administrative costs. We calculated the total net costs (operating costs plus capital expenditures minus revenues) for each year under different measures between 2022 and 2060 using a discount rate of 3.5% (0-7%)[3].

Supplementary Table 38. Fossil raw material costs ($ per Ton)

Types of fossil raw materials	Prices
Crude oil	608
Coal	120
Natural Gas	870

Supplementary Table 39. Power system costs ($ per kW·h)

	Prices
Thermal Power Generation	0.06192
Hydroelectricity	0.00774
Nuclear Power Generation	0.03792
Wind Power	0.02709
Solar Power	0.03328

Supplementary Table 40. Steam Energy Costs ($ per Ton)

	Prices
Coal	22.8
Natural Gas	9.75

Supplementary Table 41. Costs of various aspects of waste management ($ per Ton)

Segment	Prices
Collection	115
Sorting	156

Mechanical Recycling	397
Chemical Recycling	249
Mechanical recycling benefits	950
Chemical recycling benefits	637
Incineration	49
Incineration benefits	34
Landfill	30

Supplementary Table 42. Costs and benefits of different alternatives ($ per Ton)

	Collection	Recycling	Recycling benefits	Incineration	Landfill
Paper	113	852	600	15	30
Biodegradable Plastics	75	20		15	30
Glass	75	35	57	15	30
Bamboo	75			15	30

Supplementary Method 8. Uncertainty analysis

In material flow analysis, Monte Carlo analysis is an effective tool for assessing the impact of uncertainty in model input parameters on the model's predictive outcomes. In plastic material flow analysis, the uncertainty range for each input variable is determined based on the quality and source of the data (i.e., the data pedigree). These uncertainties naturally propagate throughout the Monte Carlo simulation process, thereby affecting the predicted outcomes of plastic flow and quality changes. To obtain more precise predictions, we conducted 1000 Monte Carlo simulations of the plastic flow process.

Supplementary Table 43. Data Pedigree Scoring Matrix.

Indicator	Reliability	Completeness	Temporal relevance	Geographic relevance	Other relevance
Definition	Focus on data sources: documentation of data generation, such as sampling methods, validation methods, and assessment of the review process.	Composition of all relevant quality flow rates by date. Assess potential overestimation or underestimation.	Consistency of available dates and ideal dates relative to the temporal reference.	Consistency of available dates and ideal dates in terms of geographic reference.	Consistency between available dates and ideal dates in terms of technology, product, etc.
Score: 1	The data generation methods are well-documented and subject to consistent peer-reviewed data.	The value includes all relevant processes/pathways.	The value is associated with the correct time period.	The value is relevant to the study area.	The value is associated with the same product, same technology, etc.
Score: 2	The data generation methods are described but not entirely transparent; no	The value quantitatively encompasses the main processes/pathways under	Numerical deviation for a period of 1 to 5 years.	The value is relevant to similar socio-economic regions (gross	The value associated with similar technologies, products, etc.

	verification is conducted.	discussion.		domestic product, consumption patterns).	
Score: 3	The methods are not fully described, but the principles of data generation are clear; no validation has been performed.	The value includes some important processes/pathways and the certainty of data gaps.	Value deviation over a period of 5 to 10 years.	Regions with slightly different socio-economic conditions.	The value set differs from the technology/product of interest but can establish a rough correlation based on experience or data.
Score: 4	The methods of data generation are unknown, with no available documentation.	Only scattered data is available; key processes/large-scale flows are missing.	Deviation exceeds 10 years.	Regions with significantly different socio-economic conditions.	The value is significantly deviant from the technology/product of interest, with ambiguous and speculative correlations.

Supplementary Table 44. Assignment of data pedigree levels for model inputs.

Score	Reliability	Completeness	Temporal relevance	Geographic relevance	Other relevance
Resin-intermediate (tracing back)	2	2	2	1	1
Plastic recycling rate	2	2	1	1	1
Plastic mismanagement rate	2	2	1	1	1
Plastic leakage rate	2	2	1	1	1
Population	2	2	3	1	2
Per capita plastic consumption	2	2	3	1	2
Energy structure	1	1	3	1	2
Design optimization for reduction	2	3	3	3	2
Reduction in demand	2	3	3	3	2
Reduction through substitution	3	3	3	3	2
Collection					
Plastic mechanical recycling rate	3	3	3	2	3
Plastic mismanagement rate	3	3	3	2	3
Closed-loop loss share	2	2	2	2	2

Open-loop loss share	2	2	2	2	2
Proportion of safe control at landfills	2	2	2	2	2
Proportion of dumping sites/unhygienic landfills contributing to aquatic pollution	3	3	4	3	3
Proportion of dumping sites/unhygienic landfills contributing to terrestrial pollution	3	3	4	3	3

Supplementary Table 45. Uncertainty scores.

	Reliability	Completeness	Temporal relevance	Geographic Relevance	Other Relevance	coefficients of variation (CV, standard deviation divided by mean)
**Historical data**						
Chemical raw materials	2	2	2	1	1	8.21
recycling rate	2	2	1	1	1	8.21
mismanagement rate	2	2	1	1	1	8.21
Plastic Leakage rate	2	2	1	1	1	8.21
**projection**						
**Basic information**						
population	2	2	3	1	2	10.41
Plastic consumption per capita	2	2	3	1	2	10.41
**carbon reduction**						
Optimisation of the energy structure	1	1	3	1	2	8.21
**Reduce**						
Reduction potential - design reduction	2	3	3	3	2	13.79
Reduced potential - reduced demand	2	3	3	3	2	13.79
substitution	3	3	3	3	2	23.84
**collection**						

Mechanical recycling rate	3	3	3	2	3	23.84
Plastic mismanagement rate	3	3	3	2	3	23.84
**recycle**						
Share of closed-loop losses	2	2	2	2	2	8.21
Share of open-loop losses	2	2	2	2	2	8.21
**disposal**						
Safety control of post-collection waste	2	2	2	2	2	8.21
landfill rate	2	2	2	2	2	8.21
**mismanagement**						
Proportion of open burning not collected	3	3	4	3	3	31.42
Leakage rate in the terrestrial environment	3	3	4	3	3	31.42
Leakage rate in the aquatic environment	3	3	4	3	3	31.42
Proportion of dumpsites/unsanitary landfills with open burning	3	3	4	3	3	31.42
Proportion of aquatic pollution from dumpsites/unsanitary landfills	3	3	4	3	3	31.42
Proportion of land-based pollution from dumpsites/unsanitary landfills	3	3	4	3	3	31.42

Supplementary References

1. Jambeck, J. R. *et al.* Plastic waste inputs from land into the ocean. *Science* **347**, 768–771 (2015).

2. Lebreton, L. C. M. *et al.* River plastic emissions to the world's oceans. *Nat. Commun.* **8**, 15611 (2017).

3. Lau, W. W. Y. *et al.* Evaluating scenarios toward zero plastic pollution. *Science* **369**, 1455–1461 (2020).

4. Schmidt, C., Krauth, T. & Wagner, S. Export of Plastic Debris by Rivers into the Sea. *Environ. Sci. Technol.* **51**, 12246–12253 (2017).

5. Mai, L. *et al.* Global Riverine Plastic Outflows. *Environ. Sci. Technol.* **54**, 10049–10056 (2020).

6. Meijer, L. J. J., van Emmerik, T., van der Ent, R., Schmidt, C. & Lebreton, L. More than 1000 rivers account for 80% of global riverine plastic emissions into the ocean. *Sci. Adv.* **7**, eaaz5803 (2021).

7. Cottom, J. W., Cook, E. & Velis, C. A. A local-to-global emissions inventory of macroplastic pollution. *Nature* **633**, 101–108 (2024).

8. Anshassi, M. & Townsend, T. G. Improving waste systems in the global south to tackle international environmental impacts. *Nat. Sustain.* 1–11 (2025) doi:10.1038/s41893-025-01607-8.

9. Luan, X. *et al.* Estimation and prediction of plastic losses to the environment in China from 1950 to 2050. *Resour. Conserv. Recycl.* **184**, 106386 (2022).

10. Strokal, M. *et al.* River export of macro- and microplastics to seas by sources worldwide. *Nat. Commun.* **14**, 4842 (2023).

11. Zheng, J. & Suh, S. Strategies to reduce the global carbon footprint of

plastics. *Nat. Clim. Change* **9**, 374–378 (2019).

12. Cabernard, L., Pfister, S., Oberschelp, C. & Hellweg, S. Growing environmental footprint of plastics driven by coal combustion. *Nat. Sustain.* **5**, 139–148 (2022).

13. Stegmann, P., Daioglou, V., Londo, M., van Vuuren, D. P. & Junginger, M. Plastic futures and their CO2 emissions. *Nature* **612**, 272–276 (2022).

14. Luan, X. *et al.* Greenhouse gas emissions associated with plastics in China from 1950 to 2060. *Resour. Conserv. Recycl.* **197**, 107089 (2023).

15. Pottinger, A. S. *et al.* Pathways to reduce global plastic waste mismanagement and greenhouse gas emissions by 2050. *Science* **386**, 1168–1173 (2024).

16. China Plastics Processing Industry Association, 2001-2021. China Plastics Industry Yearbook. China Plastics Processing Industry Association, Beijing.

17. Luan, X. *et al.* Dynamic material flow analysis of plastics in China from 1950 to 2050. *J. Clean. Prod.* **327**, 129492 (2021).

18. Chen, L., Liu, W., Yang, T. & Nowack, B. Probabilistic material flow analysis of eight commodity plastics in China: Comparison between 2017 and 2020. *Resour. Conserv. Recycl.* **191**, 106880 (2023).

19. An, J., Wu, F., Wang, D. & You, J. Estimated material metabolism and life cycle greenhouse gas emission of major plastics in China: A commercial sector-scale perspective. *Resour. Conserv. Recycl.* **180**, 106161 (2022).

20. Chu, J. *et al.* Flows and waste reduction strategies of PE, PP, and PET plastics under plastic limit order in China. *Resour. Conserv. Recycl.* **188**, 106668 (2023).

21. Jiang, X. *et al.* Assessment of Plastic Stocks and Flows in China: 1978-2017.

Resour. Conserv. Recycl. **161**, 104969 (2020).

22. Chu, J. *et al.* Life-cycle greenhouse gas emissions and the associated carbon-peak strategies for PS, PVC, and ABS plastics in China. *Resour. Conserv. Recycl.* **182**, 106295 (2022).

23. Zhou, Y., Yang, N. & Hu, S. Industrial metabolism of PVC in China: A dynamic material flow analysis. *Resour. Conserv. Recycl.* **73**, 33–40 (2013).

24. Liu, Y., Zhou, C., Li, F., Liu, H. & Yang, J. Stocks and flows of polyvinyl chloride (PVC) in China: 1980-2050. *Resour. Conserv. Recycl.* **154**, 104584 (2020).

25. Chu, J. *et al.* Dynamic flows of polyethylene terephthalate (PET) plastic in China. *Waste Manag.* **124**, 273–282 (2021).

26. Sun, N. *et al.* Material Flow analysis of plastics from provincial household appliances in China: 1978–2016. *Waste Manag.* **153**, 156–166 (2022).

27. Yi, Y., Wang, Z., Wennersten, R. & Sun, Q. Life Cycle Assessment of Delivery Packages in China. *Energy Procedia* **105**, 3711–3719 (2017).

28. Nakatani, J., Fujii, M., Moriguchi, Y. & Hirao, M. Life-cycle assessment of domestic and transboundary recycling of post-consumer PET bottles. *Int. J. Life Cycle Assess.* **15**, 590–597 (2010).

29. Drewniok, M. P., Gao, Y., Cullen, J. M. & Cabrera Serrenho, A. What to Do about Plastics? Lessons from a Study of United Kingdom Plastics Flows. *Environ. Sci. Technol.* **57**, 4513–4521 (2023).

30. Geyer, R., Jambeck, J. R. & Law, K. L. Production, use, and fate of all plastics ever made. *Sci. Adv.* **3**, e1700782 (2017).

31. Drewniok, M. P., Gao, Y., Cullen, J. M. & Cabrera Serrenho, A. What to Do about Plastics? Lessons from a Study of United Kingdom Plastics Flows. *Environ.*

Sci. Technol. https://doi.org/10.1021/acs.est.3c00263 (2023) doi:10.1021/acs.est.3c00263.

32. Tramoy, R. *et al.* Assessment of the Plastic Inputs From the Seine Basin to the Sea Using Statistical and Field Approaches. *Front. Mar. Sci.* **6**, (2019).

33. van Emmerik, T., Strady, E., Kieu-Le, T.-C., Nguyen, L. & Gratiot, N. Seasonality of riverine macroplastic transport. *Sci. Rep.* **9**, 13549 (2019).

34. Luo, Z. *et al.* Environmental occurrence, fate, impact, and potential solution of tire microplastics: Similarities and differences with tire wear particles. *Sci. Total Environ.* **795**, 148902 (2021).

35. Li, K., Su, H., Xiu, X., Liu, C. & Hao, W. Tire wear particles in different water environments: occurrence, behavior, and biological effects—a review and perspectives. *Environ. Sci. Pollut. Res.* **30**, 90574–90594 (2023).

36. Kole, P. J., Löhr, A. J., Van Belleghem, F. G. A. J. & Ragas, A. M. J. Wear and Tear of Tyres: A Stealthy Source of Microplastics in the Environment. *Int. J. Environ. Res. Public. Health* **14**, 1265 (2017).

37. Bai, M., Zhu, L., An, L., Peng, G. & Li, D. Estimation and prediction of plastic waste annual input into the sea from China. *Acta Oceanol. Sin.* **37**, 26–39 (2018).

38. National Data. https://data.stats.gov.cn/english/.

39. Yuelu Zhao. Environmental Impact Analysis of Ethylene Production Based on Life Cycle Approach. https://doi.org/10.26991/d.cnki.gdllu.2021.002463 (2021) doi:10.26991/d.cnki.gdllu.2021.002463.

40. Liang, H. *et al.* Combining LCA-MFA models to identify China's plastic value chain environmental impact mitigation pathways. *iScience* **26**, 107701 (2023).

41. Kaijun Zhang. China caprolactam production status and production process technology and economic analysis comparison. *Chem. Manag.* 121–122 (2021) doi:10.19900/j.cnki.ISSN1008-4800.2021.18.060.

42. Kan Wang. Life cycle assessment of brake friction materials. (2015).

43. Zhou, X. *et al.* Life cycle assessment of polycarbonate production: Proposed optimization toward sustainability. *Resour. Conserv. Recycl.* **189**, 106765 (2023).

44. Ming Feng. A study of the medium- and long-term potential growth rate of the Chinese economy: progress, consensus and divergence. *Financ. Think Tank* 29-57+140-141 (2020) doi:10.20032/j.cnki.cn10-1359/f.2020.05.003.

45. Projections of medium- and long-term trends in population change in China（2021-2050）. *UNFPA China* https://china.unfpa.org/zh-Hans/publications/22070101 (2023).

46. The Global Commitment Five Years In Learnings to Accelerate Towards a Future Without Plastic Waste or Pollution.pdf.

47. China Government Network. “Action Program on Plastic Pollution Control in the 14th Five-Year Plan. https://www.gov.cn/zhengce/zhengceku/2021-09/16/content_5637606.htm.

48. Chinese Academy of Environmental Planning, Beijing Normal University, Sun Yat-Sen University, China City Greenhouse Gas Working Group. China Products Carbon Footprint Factors Database(2022)[R]. Beijing, 2022. https://lca.cityghg.com/.

49. National Think Tank. Survey Report on the Current Situation of Recycling of Low-Value Recyclables in China（2023）.

50. van der Hulst, M. K. *et al.* Greenhouse gas benefits from direct chemical recycling of mixed plastic waste. *Resour. Conserv. Recycl.* **186**, 106582 (2022).

51. Sun, Y. *et al.* China's roadmap to plastic waste management and associated economic costs. *J. Environ. Manage.* **309**, 114686 (2022).
