# Supplementary material for: Strategies for synergistic reduction of plastic leakage and greenhouse gas emissions in China
Source: Nat Commun. 2026 Feb 25;17:3178. doi: 10.1038/s41467-026-69893-0 (PMC13046867; doi:10.1038/s41467-026-69893-0)
Supplement: Supplementary file 2 — Reporting Summary [file 41467_2026_69893_MOESM2_ESM.pdf]

nature portfolio

Corresponding author(s): Wenchao Ma

Last updated by author(s): Feb 6, 2026

Reporting Summary

Nature Portfolio wishes to improve the reproducibility of the work that we publish. This form provides structure for consistency and transparency in reporting. For further information on Nature Portfolio policies, see our Editorial Policies and the Editorial Policy Checklist.

Statistics

For all statistical analyses, confirm that the following items are present in the figure legend, table legend, main text, or Methods section.

n/a	Confirmed	
☐	☒	The exact sample size (n) for each experimental group/condition, given as a discrete number and unit of measurement
☒	☐	A statement on whether measurements were taken from distinct samples or whether the same sample was measured repeatedly
☒	☐	The statistical test(s) used AND whether they are one- or two-sided<br>*Only common tests should be described solely by name; describe more complex techniques in the Methods section.*
☒	☐	A description of all covariates tested
☒	☐	A description of any assumptions or corrections, such as tests of normality and adjustment for multiple comparisons
☐	☒	A full description of the statistical parameters including central tendency (e.g. means) or other basic estimates (e.g. regression coefficient) AND variation (e.g. standard deviation) or associated estimates of uncertainty (e.g. confidence intervals)
☒	☐	For null hypothesis testing, the test statistic (e.g. F, t, r) with confidence intervals, effect sizes, degrees of freedom and P value noted<br>*Give P values as exact values whenever suitable.*
☒	☐	For Bayesian analysis, information on the choice of priors and Markov chain Monte Carlo settings
☒	☐	For hierarchical and complex designs, identification of the appropriate level for tests and full reporting of outcomes
☒	☐	Estimates of effect sizes (e.g. Cohen's d, Pearson's r), indicating how they were calculated

Our web collection on statistics for biologists contains articles on many of the points above.

Software and code

Policy information about availability of computer code

Data collection	We do not use specific software to collect data.
Data analysis	Plastic substance flows, leakage and GHG emission flows were mapped using e!Sankey (v5.12). Scenario analysis and cost–benefit analysis maps were plotted using OriginPro 2024 (OriginLab). Plastic inventory curve fitting and projections to 2060 were performed in Python (v3.11).Custom code used for Monte Carlo simulations, scenario analysis and optimisation is available at figshare: [https://doi.org/10.6084/m9.figshare.29875109].

For manuscripts utilizing custom algorithms or software that are central to the research but not yet described in published literature, software must be made available to editors and reviewers. We strongly encourage code deposition in a community repository (e.g. GitHub). See the Nature Portfolio guidelines for submitting code & software for further information.

Data

Policy information about availability of data

All manuscripts must include a data availability statement. This statement should provide the following information, where applicable:

- Accession codes, unique identifiers, or web links for publicly available datasets
- A description of any restrictions on data availability
- For clinical datasets or third party data, please ensure that the statement adheres to our policy

Input data used in this study are available from the following third-party sources: (1) Data on the production, consumption and trade of primary plastics in China (1992–2021) can be accessed through the China Plastics Industry Yearbook. (2) Trade data for primary plastic products, finished plastic products and plastic waste are available from the online statistical data service platform of the General Administration of Customs of the People's Republic of China and from the United Nations Comtrade database. (3) Parameters used for environmental leakage and GHG emissions are compiled from published literature; all primary sources and parameter values used in this study are documented in the Supplementary Information. (4) Population projections are available from the "Forecast of Medium and Long Term Change Trend of Chinese Population" compiled by the China Population and Development Research Center and the United Nations Population Fund China Representative Office. (5) Future per-capita GDP projections are derived from published literature; the primary sources are documented in the Supplementary Information. The processed datasets generated in this study (including the national time series of plastic substance flows, environmental leakage and associated GHG emissions for 1992–2021, and scenario outputs for 2022–2060) are available in a public repository at figshare (DOI: [https://doi.org/10.6084/m9.figshare.29875109]).

Research involving human participants, their data, or biological material

Policy information about studies with human participants or human data. See also policy information about sex, gender (identity/presentation), and sexual orientation and race, ethnicity and racism.

Reporting on sex and gender	n/a, since this research does not involve human participants, their data, or biological material.
Reporting on race, ethnicity, or other socially relevant groupings	n/a, since this research does not involve human participants, their data, or biological material.
Population characteristics	n/a, since this research does not involve human participants, their data, or biological material.
Recruitment	n/a, since this research does not involve human participants, their data, or biological material.
Ethics oversight	n/a, since this research does not involve human participants, their data, or biological material.

Note that full information on the approval of the study protocol must also be provided in the manuscript.

Field-specific reporting

Please select the one below that is the best fit for your research. If you are not sure, read the appropriate sections before making your selection.

☐ Life sciences ☐ Behavioural & social sciences ☒ Ecological, evolutionary & environmental sciences

For a reference copy of the document with all sections, see nature.com/documents/nr-reporting-summary-flat.pdf

Ecological, evolutionary & environmental sciences study design

All studies must disclose on these points even when the disclosure is negative.

Study description	We analyzed material flows, environmental leakage, and GHG emissions of 14 plastic polymers in China (1992–2021), projecting co-benefit potentials through 2060 under 14 policy scenarios with cost-benefit dynamics.
Research sample	14 plastic types in China.
Sampling strategy	The 14 plastic types are determined by consumption and data availability.
Data collection	Input data used in this study are available from the following third-party sources: (1) Data on the production, consumption and trade of primary plastics in China (1992–2021) can be accessed through the China Plastics Industry Yearbook. (2) Trade data for primary plastic products, finished plastic products and plastic waste are available from the online statistical data service platform of the General Administration of Customs of the People's Republic of China and from the United Nations Comtrade database. (3) Parameters used for environmental leakage and GHG emissions are compiled from published literature; all primary sources and parameter values used in this study are documented in the Supplementary Information. (4) Population projections are available from the "Forecast of Medium and Long Term Change Trend of Chinese Population" compiled by the China Population and Development Research Center and the United Nations Population Fund China Representative Office. (5) Future per-capita GDP projections are derived from published literature; the primary sources are documented in the Supplementary Information. The processed datasets generated in this study (including the national time series of plastic substance flows, environmental leakage and associated GHG

emissions for 1992–2021, and scenario outputs for 2022–2060) are available in a public repository at figshare (DOI: [https://doi.org/10.6084/m9.figshare.29875109]).

Timing and spatial scale	The timing scale is from 1992 to 2060, and the spatial scale is China.
Data exclusions	No data is excluded.
Reproducibility	This study can be reproduced using the data and equations reported in the main text and supplementary information.
Randomization	n/a, since this study does not need to randomize the data
Blinding	n/a, since this study does not involve experiments to other agents.

Did the study involve field work? ☐ Yes ☒ No

Reporting for specific materials, systems and methods

We require information from authors about some types of materials, experimental systems and methods used in many studies. Here, indicate whether each material, system or method listed is relevant to your study. If you are not sure if a list item applies to your research, read the appropriate section before selecting a response.

Materials & experimental systems

n/a	Involved in the study	
☒	☐	Antibodies
☒	☐	Eukaryotic cell lines
☒	☐	Palaeontology and archaeology
☒	☐	Animals and other organisms
☒	☐	Clinical data
☒	☐	Dual use research of concern
☒	☐	Plants

Methods

n/a	Involved in the study	
☒	☐	ChIP-seq
☒	☐	Flow cytometry
☒	☐	MRI-based neuroimaging

Plants

Seed stocks	n/a,since since this study does not involve plants.
Novel plant genotypes	n/a,since since this study does not involve plants.
Authentication	n/a,since since this study does not involve plants.
